# The *Osgin* Gene Family: Underexplored Yet Essential Mediators of Oxidative Stress

**DOI:** 10.3390/biom15030409

**Published:** 2025-03-13

**Authors:** Grace Hussey, Marcus Royster, Nivedha Vaidy, Michael Culkin, Margaret S. Saha

**Affiliations:** Biology Department, William & Mary, Williamsburg, VA 23185, USA; gehussey@wm.edu (G.H.); moroyster@wm.edu (M.R.); nvaidy@wm.edu (N.V.); maculkin@wm.edu (M.C.)

**Keywords:** *Osgin1*, *Osgin2*, oxidative stress, flavin-dependent monooxygenase, tumorigenesis

## Abstract

The *Osgin* gene family consists of two members, *Osgin1* and *Osgin2*, involved in the cellular oxidative stress response. While many members of this essential cellular pathway have been extensively characterized, the *Osgin* gene family, despite its broad phylogenetic distribution, has received far less attention. Here, we review published articles and open-source databases to synthesize the current research on the evolutionary history, structure, biochemical and physiological functions, expression patterns, and role in disease of the *Osgin* gene family. Although *Osgin* displays broad spatiotemporal expression during development and adulthood, there is ambiguity regarding the cellular functions of the OSGIN proteins. A recent study identified OSGIN-1 as a flavin-dependent monooxygenase, but the biochemical role of OSGIN-2 has not yet been defined. Moreover, while the *Osgin* genes are implicated as mediators of cell proliferation, apoptosis, and autophagy, these functions have not been connected to the enzymatic classification of OSGIN. Misregulation of *Osgin* expression has long been associated with various disease states, yet recent analyses highlight the mechanistic role of OSGIN in pathogenesis and disease progression, underscoring the therapeutic potential of targeting OSGIN. In light of these findings, we suggest further avenues of research to advance our understanding of this essential, yet underexplored, gene family.

## 1. Introduction

To provide the proper context for understanding the importance of *Osgin* (oxidative stress-induced growth inhibitor), it is necessary to review the ancient and highly conserved reactive oxygen species (ROS) pathway. The addition of oxygen to the Earth’s atmosphere ~2.45 billion years ago and the evolution of aerobic respiration placed selective pressure on organisms to develop mechanisms to both utilize ROS and survive ROS-related toxicity.

This led to the development of a highly complex system of oxidative stress genes and pathways that are designed to mitigate or utilize oxidative stress as needed [1]. Largely conserved across organisms employing aerobic metabolism, ROS are chemically active free radical and non-radical oxygen species created from the partial reduction of oxygen. ROS are produced endogenously in cellular processes including oxidative metabolism, endoplasmic reticulum (ER) stress, peroxisomal enzyme activity, activity of redox enzymes, as well as interactions with exogenous agents [2,3,4,5].

At physiological levels, ROS are referred to as oxidative eustress or “good stress” and are vital to the regulation of biochemical transformations [6], cellular growth, differentiation, protection, apoptosis, immune response, transcription factor regulation, adhesion, DNA damage response, iron regulation [7], wound healing [8], and stress adaptation due to their involvement in intracellular signaling [2,4,6,9]. However, when ROS levels increase and an imbalance between free radical formation and mitigation is created, oxidative stress—a disruption of redox homeostasis that occurs when the prooxidant–antioxidant system shifts in favor of the prooxidants—is produced. This can result in macromolecular damage and disruption of cell signaling pathways, which have been implicated in pathologies including neurodegenerative diseases and cancer [7,9,10,11,12,13].

A system of enzymatic—which break down ROS into less reactive byproducts—and non-enzymatic antioxidants—which serve as free radical absorbers—function to regulate intracellular ROS homeostasis and prevent accumulation of ROS and oxidative stress [9,13,14,15,16,17,18]. Additionally, cells are often capable of repairing damage caused by ROS utilizing reductase enzymes and base excision repair to reverse the harmful effects of oxidative stress-related modifications [13]. Accumulation of ROS can also stimulate cellular pathways that promote the expression of genes that allow for adaptation to oxidative stress by regulating cell proliferation, differentiation, autophagy, and apoptosis [9].

One such gene involved in this oxidative stress pathway, *Osgin1*, also known as OKL38 (ovary, kidney, and liver protein 38) and BDGI (bone marrow stromal cell-derived growth inhibitor), was first identified by Huynh et al. (2001) in an experiment to isolate upregulated genes in rat mammary epithelial cells during pregnancy and lactation [19]. Although *Osgin1* was expressed ubiquitously in all examined tissues, the mRNA transcripts were most abundant in ovary, kidney, and liver tissues [19]. Thus, Huynh et al. named the gene OKL38.

However, the gene became known as *Osgin1* once its expression was stimulated by superoxide production following oxidized 1-palmitoyl-2-arachidonoyl-sn-glycero3-phosphorylcholine (OxPAPC) treatment and it was implicated in the oxidative stress response [20]. The novel *Osgin2* gene, originally known as C8orf1 (chromosome 8 open reading frame 1) or hT41 (human testis 4.1-kb transcript), was identified by Tauchi et al. (1999) while characterizing the neighboring *NBS1* gene due to its implication in Nijmegen breakage syndrome (NBS). While *Osgin2* was also expressed ubiquitously, its expression was highest in ovary and testis tissues [21]. For the purposes of this review, these genes will solely be referred to as *Osgin1* and *Osgin2* and their proteins as OSGIN-1 and OSGIN-2, respectively, unless otherwise specified. Since their initial discovery, the *Osgin* genes have been implicated in the cellular response to oxidative stress. However, while the mechanisms of the oxidative stress pathway—as reviewed in Hong et al. (2024)—are well characterized, the role of the *Osgin* gene family in this process remains poorly understood [9]. The goal of this review is to provide a comprehensive overview of the current state of *Osgin* research, and due to their largely conserved presence across aerobic species, we suggest future research directions for the essential, yet understudied, *Osgin* gene family.

## 2. Methods

A systematic and intensive review of the current literature was performed using PubMed as a database and Google Scholar as an academic search engine. Gene conservation and expression data were curated from the OrthoDB and National Center for Biotechnology Information (NCBI) databases, as well as the ExpressionAtlas, Bgee, Mouse Genome Informatics, GeneCards, Rat Genome Database, Allen Mouse Brain Atlas, Xenbase, VectorBase, Echinobase, and WormBase databases. Key search terms used were oxidative stress-induced growth inhibitor, *Osgin*, *Osgin1*, *Osgin2*, OKL38, BDGI, c8orf1, and hT41. No limitations were placed on article publication date. Identified articles and data were reviewed in their entirety. Articles were excluded if they did not meet the criteria of being: 1. peer-reviewed; 2. written in or translated to English; or 3. not withdrawn. We organized this review to address every characteristic traditionally associated with examining a gene family, including phylogeny and evolutionary history, biochemical and physiological functions, expression throughout development and adulthood, and role in disease. However, the length and depth of the sections were determined by the availability of the literature. 

Phylogenetic trees were constructed from protein amino acid sequences retrieved from the OrthoDB and NCBI databases, as well as NCBI BLAST v.2.16.0 search results [22,23]. Sequences were aligned using CLUSTALW v.2.1 software, and the best-fit amino acid substitution model was selected using MEGA11 v.11.0.13 (Molecular Evolutionary Genetics Analysis) [24,25]. Maximum likelihood (ML) trees were assembled using RAxML-NG v.1.2.2 accessed through the CIPRES Science Gateway v.3.3 [26,27]. Robustness of topologies was assessed using the bootstrap method. Trees were visualized using FigTree v.1.4.4 [22].

## 3. Phylogeny and Evolution of *Osgin* Genes

The current available genome data support that *Osgin1* and *Osgin2* represent a rich and phylogenetically diverse gene family. The *Osgin* genes are paralogs, related through a gene duplication event. While data suggest that these genes are confined to the domain Eukarya, within this group, they are highly conserved throughout vertebrate species and are found widely within a number of invertebrate phyla, and fungal and protist species. Despite this intriguing evolutionary history, little phylogenetic analysis of these genes has been performed. Ong et al. (2004b) and Goupil et al. (2024) provide the most current in-depth analyses of conservation of the *Osgin* gene family, performing amino acid alignment demonstrating conservation in the coding regions of *Osgin 1* orthologs among rats, mice, *C. elegans*, and humans [23,28]. These studies also demonstrated conservation of function across these orthologous proteins. Beyond this analysis and the use of BLASTp comparing similarity between *Osgin* paralogs by Satta et al. (2023) and Hu et al. (2014), no complete investigation into the phylogenetics of *Osgin* has been performed in the period of over 20 years since its initial discovery [29,30].

In the first analysis of *Osgin* inter-species conservation, Ong et al. (2004b) analyzed three novel rat *Osgin1* cDNAs and compared the sequences with *H. sapiens* and *M. musculus* [28]. Ong et al. (2004b) employed BLAST to determine that the 52 kDa OSGIN-1 isoform in *R. norvegicus* shares 85% and 90% nucleotide sequence homology with its 52 kDa isoform orthologs in *Homo sapiens* and *Mus musculus* (GenBank: BC022135.1), respectively. Multiple sequence alignment further showed that rat and mouse (GenBank: BC006032.1) OSGIN-1 orthologs share 91% amino acid and 93% nucleotide sequence identity; while rat and human OSGIN-1 orthologs share 85% nucleotide identity and 80% amino acid identity. When investigating conservation between the human and rat 52 kDa isoforms, Ong et al. (2004b) noted conservation in the coding region but not in the 5′ UTRs, prompting the authors to suggest that recent evolution may have led to the variation in the regulation of these genes [28].

Moreover, Ong. et al. (2004b) postulated that the high degree of similarity between the coding regions of these OSGIN-1 orthologs may indicate a conserved function, a theory supported by additional experimental evidence [28]. Further BLAST searches against the non-redundant database revealed the similarity of OSGIN-1 to several proteins of unknown function, including human C8ORF1 protein (GenBank accession no. NP_004328), now known as OSGIN-2, with an identity of 49% and a similarity of 62%; and the *Bacillus halodurans* protein BH1623 (GenBank accession no. NP_242489), now referred to as a YpdA family putative bacillithiol disulfide reductase, with an identity of 39% and a similarity of 44%. These results prompted Ong et al. (2004b) to suggest that OSGIN-1, C8ORF1, and BH1623 may belong to a larger family of proteins with similar functions [28].

With the goal of assessing the conservation between the human *Osgin1* and *Osgin2* paralogs, Hu et al. (2014), in a brief review of OSGIN, performed an alignment of the amino acid sequences of the human *Osgin* genes using CLUSTALW [30]. The results demonstrated that the two paralogs share 49% sequence identity, predominantly in the C-terminal region. In a study investigating the role of *Osgin* in Nrf2-regulated endothelial detachment, Satta et al. (2023) analyzed the amino acid and structural similarities between the OSGIN proteins [29]. The authors used CLUSTALO to determine that human OSGIN-1 and OSGIN-2 share a 40.8% amino acid identity and further displayed a large degree of conservation across species. Furthermore, Satta et al. (2023) found a high degree of similarity between the tertiary protein structures of human OSGIN-1 and OSGIN-2 [29].

While earlier conservation analysis of OSGIN was limited to vertebrate species, specifically mammals, in the more recent literature, Goupil et al. (2024) used comparative sequence analysis to determine that the F30B5.4 protein (now named OSGN-1) in *C. elegans* is an ortholog of the mammalian OSGIN-1 [23]. Goupil et al. (2024) further investigated the conservation of the FAD- and NAD(P)H-binding domains in the *C. elegans Osgn1* protein with OSGIN-1 and OSGIN-2 homologs from various vertebrate species (*H. sapiens*, *Xenopus tropicalis*, *M. Musculus*, and *Danio rerio*), identifying a high degree of similarity. This suggested that protein function may be conserved across OSGIN orthologs. Goupil et al. (2024) provided experimental evidence to support the conservation of function between *H. sapiens Osgin1* and *C. elegans Osgn1* as expression of a catalytically active *Osgn1* in *Osgin1*-depleted HeLa cells rescued the binucleated phenotype during cytokinesis, suggesting that these proteins are functional orthologs [23]. It is important to note that this result does not necessarily dictate a conserved physiological function, simply sufficient conservation of biochemical function. A deeper understanding of homology in other invertebrates, as shown in Goupil et al., offers an important avenue for phylogenetic understanding of these genes.

Available phylogenetic analyses of *Osgin* are extremely limited, with no complete phylogenetic analysis currently available despite available sequence data. Recent advances in genome sequence analysis have revealed potential *Osgin* orthologs in numerous species, including fungi and protists. OrthoDB, a database that sorts publicly available genome data to identify orthologous genes, identified *Osgin1* and *Osgin2* orthologs in 1866 different species, with 505 being identified in species of fungi across the Ascomycota and Mucoromycota phyla, most prominently among Penicillium and Aspergillus [31]. Several fungal species, predominantly among Mucromycota, were identified as containing *Osgin2* orthologs. OrthoDB also identified *Osgin1* orthologs in seven species of Alveolata protists, including the protist dinoflagellate marine plankton *Symbiodinium microadriaticum;* as well as a species of Stramenopile, the unicellular algae *Tetraparma gracilis*. The conservation of *Osgin1* among fungal species is supported by other databases, including the Orthologous Matrix (OMA) browser, which has identified 57 fungal *Osgin1* orthologs and several *Osgin2* orthologs [32]. This widespread cross-kingdom conservation of *Osgin* suggests a need for a more thorough investigation of the presence and role of these genes in fungi and all non-Animalia.

**Figure 1:**
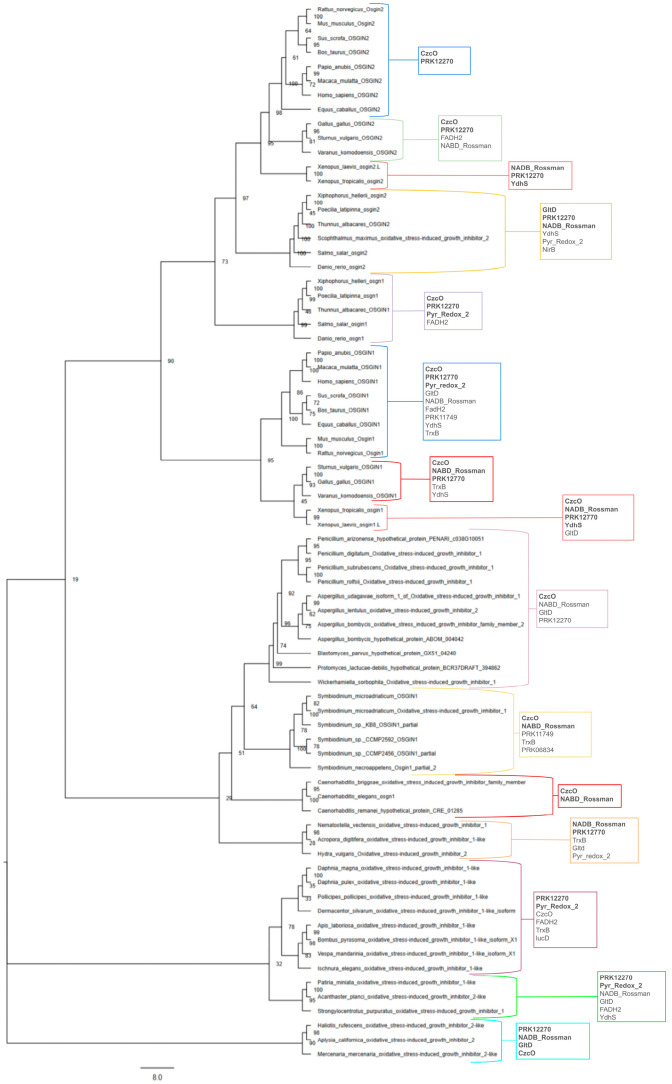
Maximum likelihood phylogenetic tree depicting evolutionary conservation of *Osgin*. The evolutionary history was inferred by using the maximum likelihood method and the JTT + G model. The tree with the highest log likelihood (−41,949.196805) is shown. The percentage of trees in which the associated taxa clustered together is shown next to the branches. The initial tree(s) for the heuristic search was obtained by generating 10 random trees and 10 parsimony trees and then selecting the topology with superior log likelihood. The tree is drawn to scale, with branches measured in the number of substitutions per site. This analysis involved 74 amino acid sequences. There were a total of 1243 sites in the final dataset. Evolutionary analyses were conducted in RAxML-NG [26]. Conserved domains within clades are shown, with those conserved in more than half of the studied species being written in bold. This diagram does not attempt any phylogenetic analyses between the homologs and is only a summary of the findings by past literature and potential homologs identified through database searches. Protein accession numbers not included in the diagram are provided in the Supplementary Materials file.

In an effort to visualize the evolutionary history of this gene family for this review using available sequence data, a phylogenetic tree was generated using publicly available genome data (Figure 1). The phylogenetic tree was created using protein sequences recorded from the OrthoDB database and NCBI BLAST search results from a diverse range of phyla and species to provide an informative picture of the evolution of the *Osgin* genes (Supplementary Accession Numbers) [31,33]. The labeling of *Osgin1* occasionally as *Osgn1* (*Danio rerio*, *Salmo salar*, *Thunnus albacares*, and *Caenorhabditis elegans*, etc.) follows a naming convention for *Osgin* genes within certain taxa rather than an indication of a separate paralogous gene [29]. Figure 1 supports that *Osgin* evolved in a common protist ancestor of animals and fungi some time prior to the divergence of the fungal and animal kingdoms ~1 BYA. The *Osgin* gene family is isolated within the eukaryotic supergroups Opisthokonta and SAR (Stramenopiles, Alveolates, and Rhizaria), which include only animals and fungi, as well as a number of protist species, respectively.

The putative evolutionary history of *Osgin1* predicted by Figure 1 mainly follows common patterns in vertebrate and invertebrate evolutionary history. However, surprisingly, *Osgin1* in bony fish separates into its own clade from other *Osgin1* orthologs, appearing to bear a closer evolutionary relationship to *Osgin2* paralogs. Protist orthologs group in a clade with fungi, indicating a greater relationship than to animal orthologs. Interestingly, *C. elegans* and other nematode *Osgin1* orthologs form a monophyletic clade with the fungal and protist orthologs, suggesting that *Caenorhabditis Osgn1* orthologs bear a closer evolutionary relationship with fungi than with invertebrate arthropods. This is a trend also seen in Parkinson and Blaxter (2002), who identified that several alcohol dehydrogenase proteins and an FAD-dependent oxidoreductase in *C. elegans* bore closer similarity to genes in *S. cerevisiae* than to *D. melanogaster* and *H. sapiens* proteins (interestingly, no *Osgin* orthologs have currently been identified in *D. melanogaster*) [34]. Parkinson and Blaxter (2002) found that in a neighbor-joining tree, these *C. elegans* proteins separated into a clade with several fungal species, with no closely related metazoan species; this led them to suggest the gain of these genes in *C. elegans* through interdomain horizontal gene transfer [34,35]. Evolution in the *Osgin2* clade more closely follows the expected vertebrate evolutionary patterns.

Information regarding phylogenetic relationships among *Osgin* genes can also be gleaned from analysis of conserved domains between OSGIN orthologs. NCBI’s Conserved Domain Database (CDD) was used to identify conserved domains between orthologs. The trends of conserved domains among OSGIN orthologs are shown in Figure 1, with the conserved domain superfamilies identified in more than half of the studied species in each group being in bold. Overall, OSGIN-1 proteins were called as containing a wider range and greater number of conserved domains, while OSGIN-2 proteins were generally called as containing only a CzcO domain and a prk12270 domain, with exceptions in bony fish and amphibians. Invertebrate orthologs appeared to follow the same trends as the OSGIN-1 orthologs, regardless of whether they were called OSGIN-1-like or OSGIN-2-like. Fungi and protists were generally called as containing only conserved domains in the CzcO superfamily, as well as the NABD_Rossman (Rossmann-fold NADPH/NADP+-binding domain) superfamily for protists. Interestingly, *Caenorhabditis* orthologs follow the same trends as fungi and protists [33,36].

SUPFAM, a superfamily database of structural and functional annotation, identified these OSGIN proteins as containing conserved domains in the FAD/NAD(P)-binding domain superfamily. Furthermore, SUPFAM classifies many of the OSGIN proteins as members of the FAD/NAD-linked reductases, N-terminal and central domains family, or the C-terminal domain of adrenodoxin reductase-like family [37]. The InterPro Protein Families and Domains Database also called these OSGIN proteins as being members of the FAD/NAD(P)-binding domain superfamily and identified them as being in the OKL38 family of proteins, a family containing many fungal and animal proteins [38]. Intriguingly, the InterPro database also putatively identified 81 bacterial proteins encoding a number of oxidoreductases, monooxygenases, and FAD/NAD(P)-binding domain-containing proteins as being members of the OKL38 family. While no database has identified *Osgin* orthologs in any prokaryotic species, this similarity serves to showcase the ubiquitous nature and conservation of oxidative stress genes across the domains of life.

In summary, the *Osgin* gene family displays a rich and diverse evolutionary history paralleling the protist origins of animals and fungi. While limited phylogenetic analysis of the *Osgin* gene family is currently available in the literature, available genomic data show that *Osgin genes* are highly conserved among a diverse array of species. Furthermore, several studies demonstrate functional and coding sequence conservation among common model organisms. While conservation in the regulation of these genes appears to be limited, studies have confirmed that there is immense evolutionary pressure for the conservation of OSGIN protein structure and function in vertebrates and invertebrates, suggesting it as a gene product essential for cellular activity. Further use of sequence comparison software such as BLASTn and BLASPp as well as conserved domain-identifying software such as NCBI CDD v.3.21 is necessary to identify further OSGIN orthologs across the kingdoms of life. Functional studies as well as phylogenetic analyses of these orthologs using multiple sequence alignment software and diverse phylogenetic tree estimation models are crucial for growing our understanding of the conservation and evolutionary history of this gene family.

## 4. Structure and Biochemical Function of OSGIN Proteins

### 4.1. Genomic Structure

While there are multiple studies investigating the genomic structure of both *Osgin* genes, there is no current consensus. Ong et al. performed the initial genomic characterization of *Osgin1*, using NCBI BLAST to localize *Osgin1* to *H. sapiens* chromosome 16q23 (2004a) and *R. norvegicus* chromosome 19q12 (2004b) [28,39]. While the localization assignments are consistent with the current *Osgin1* literature [30,40,41], the length of *Osgin1* and its exon structure are contested. To characterize the genomic structure of *Osgin1*, Ong et al. (2004a; 2004b) used dideoxy chain termination to sequence *Osgin1* cosmid clones and compared the clones with full-length *Osgin1* cDNA amplifications. In *H. sapiens*, Ong et al. (2004a) determined that *Osgin1* spans approximately 18,000 base pairs with eight exons varying from 92 to 1270 base pairs in length [39]. This conflicts with the latest NCBI RefSeq *H. sapiens* annotation (GRCh38.p14), which estimates that *Osgin1* spans approximately 13,000 base pairs with only six exons [41]. Notably, there are consistencies between the models: exons 4–8 of the eight-exon model correspond to exons 2–6 of the six-exon model. However, while the latter model describes the first exon as a 149 base pair segment followed by a 4269 base pair intron, the eight-exon model localizes exons 2 and 3 within this intron [39]. Likewise, Ong et al. (2004b) reported that *Osgin1* in *R. norvegicus* spans approximately 15,000 base pairs with eight exons, yet the current NCBI RefSeq annotation of *R. norvegicus* proposes that *Osgin1* has fifteen exons across 28,830 base pairs [28].

Ong et al. (2004a) also identified two putative promoter regions—P1 and P2—in the human *Osgin1* genome using a rapid amplification of cDNA ends (RACE) analysis and ClustalW multiple sequence alignment [39]. Using the MatInspector program, Ong et al. (2004a) identified potential transcription factor binding sites for Ikaros factor 2 (IK2) and upstream stimulating factors in both P1 and P2. However, each promoter had distinct transcription factor binding sites as well, prompting Ong et al. (2004a) to suggest that P1 and P2 are differentially regulated. Additionally, while Ong et al. (2004a) identified a core promoter motif known as an initiator (Inr) within P1, neither promoter includes a TATA or CAAT box [39]. Furthermore, using the CpG Island Searcher program, Ong et al. (2004a) identified putative CpG islands approximately 746 bp and 2500 bp upstream from the putative *Osgin1* transcriptional start sites in P1 and P2, respectively.

The genomic structure of *Osgin2* in *H. sapiens* is also inconsistently described in the current literature. Tauchi et al. (1999) conducted shotgun sequencing of five human bacterial artificial chromosome (BAC) clones and aligned these sequences with the human testis cDNA library to identify coding regions. They concluded that human *Osgin2* encompasses 25,552 base pairs on chromosome 8q21 and contains six exons between 92 and 3365 base pairs in length [21]. However, Tauchi et al. (1999) supplemented their sequence alignment data with the GENSCAN and GRAIL 2 exon prediction programs, and these results yielded different lengths for all exons except exon 5 (92 base pairs). The NCBI RefSeq *H. sapiens* annotation further complicates this by proposing that *Osgin2* comprises nine exons [41]. To date, no characterization of the *Osgin2* promoter region has been published.

### 4.2. Differential Splicing

Although the genomic structure of the *Osgin* genes is contested, several studies have identified different protein isoforms that arise from alternative splicing of the *Osgin* genomes. Building upon their analysis of *Osgin1* genomic structure in *H. sapiens* and *R. norvegicus* [28,39], Ong et al. used a 5′ RACE analysis, an in vitro transcription and translation study, and a Western blot assay to characterize different OSGIN-1 isoform in both species. In *H. sapiens*, Ong et al. (2004a) identified three OSGIN-1 isoforms with molecular weights of 52 kDa, 59 kDa, and 61 kDa from four *Osgin1* protein-coding cDNA transcripts [39]. Two of these sequences encode an OSGIN-1 isoform with a molecular weight of 52 kDa and an ORF of 477 amino acids; however, these sequences were transcribed from different promoters—P1 and P2—then underwent alternative splicing to produce identical isoforms [39]. Ong et al. (2004a) concluded that alternative splicing of the 5′ UTR and differential promoter usage contribute to the generation of various OSGIN-1 isoforms, a claim supported by Ong and Huynh (2008), who associated promoter P1 with the expression of the 52 kDa OSGIN-1 isoform [42]. The first *Osgin1* cDNA sequence isolated by Huynh et al. (2001) encoded a 34.5 kDa OSGIN-1 isoform with an open reading frame (ORF) of 317 amino acids [19]. Ong et al. (2004a) observed low levels of a similar 38 kDa OSGIN-1 isoform in their Western blot assay; however, as the 38 kDa band was in the same lane as the 52 kDa OSGIN-1 isoforms in the blot, Ong et al. (2004a) suggested this isoform may be the result of an internal start codon. In *R. norvegicus*, Ong et al. (2004b) analyzed three *Osgin1* cDNA sequences—one spanning 2.0 kb and two spanning 2.3 kb—but all proteins on the Western blot exhibited an approximate molecular weight of 52 kDa. Although post-translational modifications of the cDNA sequences were not examined in the Western blot, multiple sequence alignment revealed that the three cDNA sequences share the same open reading frame [28], suggesting that alternative splicing of *Osgin1* may be conserved across species.

Two OSGIN-2 isoforms have been preliminarily characterized in the current literature. In the first experiment to characterize *Osgin2*, Tauchi et al. (1999) used a Northern blot assay to identify three *Osgin2* cDNA sequence variants spanning 2.9, 3.8, and 4.3 kb [21]. Using NCBI BLASTx, they determined that the 4.3 kb cDNA sequence—considered to be the full-length *Osgin2* cDNA with an open reading frame of 4199 bp—is translated into a 505 amino acid protein with a molecular weight of 56.7 kDa [21,43]. An additional OSGIN-2 isoform with an open reading frame of 549 amino acids has been identified [44] but is not characterized in the literature.

### 4.3. Protein Structure

In terms of protein structure, no nuclear magnetic resonance (NMR), crystallography, or mutagenesis studies have been conducted to date to elucidate the physical structure of OSGIN-1 or OSGIN-2. However, the predicted structures of the OSGIN proteins are available in the AlphaFold Protein Structure Database. Additionally, several protein prediction software programs have identified domains within the *H. sapiens* OSGIN-1 protein. These domains include Rossman folds [23] and putative N-terminus TrkA and C-terminus Pyr-redox domains [45]. The TrkA domain, a binding site for nerve growth factor (NGF), is associated with superoxide production as NGF-induced neuronal differentiation requires generation of ROS [46], but the specific function of the TrkA domain in OSGIN-1 remains elusive. The Pyr-redox domain has been implicated in redox-dependent processes such as antioxidant defense and regulation of cellular redox state, and it is believed to be conserved in the final exon of all isoforms of *H. sapiens* OSGIN-1 [39]. Additionally, OSGIN-1 contains a bacillithiol oxidoreductase domain of the YpdA family between amino acid residues 177–471 [30]. The YpdA protein is an FAD-containing NAD(P)H-dependent oxidoreductase buffering compound involved in maintaining cellular redox homeostasis [47]. While these domains are an incomplete representation of the OSGIN proteins, their functions are consistent with the function of OSGIN proteins as mediators of oxidative stress.

### 4.4. Biochemical Function

A single study, that of Goupil et al. (2024), has identified the biochemical function of OSGIN-1, namely, as a flavin-containing monooxygenase (FMO) [23]. Prior to this publication, there was no literature classifying the biochemical function of either OSGIN protein. Based on previous work by Goupil et al. (2017)—which identified Rossman fold domains and putative FAD- and NAD(P)H-binding domains surrounding a non-canonical monooxygenase (MO) domain in OSGN-1 [48], a structure homologous to the FMO enzyme family—Goupil et al. (2024) proceeded with a DTNB-methimazole biochemical assay to verify the enzymatic activity of OSGN-1. Goupil et al. (2024) observed OSGN-1 oxidize methimazole in vitro and directly oxidized the conversion of nitro-5-thiobenzoate (TNB) into 5,5′-dithiobis(2-nitrobenzoate) (DTNB) [23]. However, in the absence of the NAD(P)H cofactor, this oxidation did not occur. When the assay was repeated with the human OSGIN-1 ortholog, Goupil et al. (2024) observed the oxidation of methimazole and binding of OSGIN-1 to the FAD cofactor. This catalytic activity suggests that both OSGN-1 and OSGIN-1 are FMOs, but the substrates of these enzymes remain unknown. This method of observing methimazole binding in a putative FMO with NADPH- and FAD-binding sites is well supported by the literature. For example, a study by Eswaramoorthy et al. (2006) used crystallography to characterize the mechanism of action of SPBP16F5.08c, a putative FMO in *Schizosaccharomyces pombe* [49]. As this FMO was classified using the same DTNB-methimazole biochemical assay, it may serve as a future point of comparison to better understand the biochemical function of OSGIN-1. Additionally, FMOs are divided into eight subclasses by functional properties, structural motifs, and the specific oxidation reaction they catalyze [50,51]. Further comparison between OSGIN-1 and other FMOs will be possible once OSGIN-1 is categorized into a subclass and its specific mechanism of action and substrate have been elucidated. This can be accomplished by performing a spectrophotometric analysis of the catalytic activity of OSGIN-1. Since the publication of Goupil et al. (2024), no further work has supported or refuted the classification of OSGIN-1 as an FMO, nor has a functional analysis of the biochemical function of OSGIN-2 been published in the literature.

Prior to the characterization of the biochemical function of OSGIN-1 in Goupil et al. (2024), the function of the OSGIN proteins was primarily explored by determining their subcellular localization and interacting partners. Upon the initial discovery of OSGIN-1, Huynh et al. (2001) determined that the protein lacks both a transmembrane domain and a nuclear localization signal peptide, prompting their hypothesis that the protein is cytosolic [19]. Goupil et al. (2024) support this assertion, as the authors used confocal time-lapse imaging and immunofluorescence to observe a cytosolic localization of OSGIN-1 in dividing HeLa cells until the late telophase, when it colocalized with RhoA to the midbody [23]. Liu et al. (2014) expanded the characterization of OSGIN-1 localization to human hepatocellular carcinoma tissue samples; they observed nuclear localization of OSGIN-1 in six of twenty-eight HCC tissues, whereas all remaining HCC tissues and non-cancerous controls had cytosolic localization [52]. Yao et al. (2008) and Hu et al. (2012) observed translocation of OSGIN-1 from the cytosol to the mitochondria and nucleoplasm, respectively, following the induction of DNA damage [45,53]. However, it is unknown how OSGIN-1 was imported into the nucleus as no canonical NLS has been identified to date. Although the subcellular localization of OSGIN-2 is less explored, Tauchi et al. (1999) used the Protein Subcellular Localization Prediction Tool (PSORT) to suggest that OSGIN-2 also has a cytosolic localization [21].

Relatively few studies have characterized the interacting partners of the OSGIN proteins. A table of putative interacting partners identified by the STRING protein–protein interaction database in *H. sapiens*, *R. norvegicus*, and *M. musculus* for OSGIN-1 and OSGIN-2 are listed in Supplementary Tables S1 and S2, respectively. As for experimental in vitro studies, Zhang et al. (2020) used an immunoprecipitation assay and a luciferase assay to suggest that OSGIN-1 regulates the activity of TFEB, a transcription factor that regulates autophagy- and lysosome-related genes [54]. Additionally, Xie et al. (2023) used an in vitro kinase assay and an in vitro binding assay to support the conclusion that OSGIN-1 increases the binding affinity between tubulin beta 3 class III (TUBB3), a mediator of microtubule dynamics, and dual specificity tyrosine phosphorylation regulated kinase 1A (DYRK1A), a kinase known to phosphorylate TUBB3 at serine 172 [55]. Enhanced OSGIN-1-mediated binding between DYRK1A and TUBB3 induces phosphorylation of TUBB3 serine 172, which effectively reduces tubulin polymerization, a mechanism of tumor migration and invasiveness [55]. Furthermore, Jia et al. (2024) supplemented bioinformatic predictions with co-immunoprecipitation experiments to identify glutamate-cysteine ligase modifier subunit (GCLM), the rate-limiting enzyme in glutathione (GSH) synthesis, as OSGIN-1-binding proteins [56]. Most recently, Deng et al. (2025) performed a Western blot assay in OSGIN-1 overexpression cells to identify OSGIN-1 as a putative modulator of the interaction between ataxia-telangiectasia mutated (ATM) and AMP-activated protein kinase (AMPK) to regulate the AMPK signaling pathway [57]. Yet, some studies provide conflicting evidence regarding the mechanism of these putative OSGIN-1 interactions. Yao et al. (2008) used a ChIP assay to identify PADI4 as a negative regulator of OSGIN-1 expression in breast cancer MCF-7 cells [53]. However, Brennan et al. (2017) challenged this claim as they determined that PADI4 knockdown does not affect OSGIN-1 expression in human astrocytes [58]. Instead, Brennen et al. (2017) proposed that OSGIN-1 regulates PADI4 transcription. To date, no in vitro studies have characterized interacting partners of OSGIN-2.

## 5. Physiological Function of OSGIN Proteins

The classification of OSGIN-1 as a flavin-containing monooxygenase (FMO) by Goupil et al. (2024) represents a significant advancement in elucidating the role of the OSGIN proteins. However, further functional experiments are necessary to confirm this classification and to clarify the relationship between the FMO activity of OSGIN-1 and its role as a mediator of oxidative stress. The current literature provides limited experimental characterization of the OSGIN proteins, with a notable gap in the literature on OSGIN-2. Nevertheless, the functional studies that have been conducted, summarized in Table 1 and Table 2 with more extensive information available in Supplementary Tables S3 and S4, have implicated the OSGIN proteins in regulating key cellular processes, including cell proliferation, autophagy, and apoptosis.

### 5.1. Role of OSGIN in Detecting and Countering Oxidative Stress

Several functional studies have highlighted the role of the OSGIN proteins in detecting and countering oxidative stress by modulating molecular responses to phospholipid oxidation, apoptosis, and autophagy. For example, Li et al. (2007) reported that OxPAPC, an oxidized modified LDL, stimulates *Osgin1* expression through NADPH oxidase (Nox) signaling [20]. Nox signaling, however, induces superoxide production [20]. Treatment with NAC, a superoxide scavenger, negates OxPAPC stimulation of *Osgin1*, thereby suggesting that *Osgin1* responds to oxidative stress produced by oxidized LDLs [20]. Hammad et al. (2009) further supported this assertion when they treated human U937 cells with oxLDL-containing immune complexes and observed an upregulation of *Osgin1* expression following oxidized LDL treatment in human U937 cells [59]. These results implicate *Osgin1* in the anti-inflammatory response to phospholipid oxidation. Of note, siRNA knockdown studies have implicated *Osgin1* as a regulator of several inflammatory and anti-inflammatory genes, as well as the inflammatory response molecules IL-8, ATF4, and KLF4 [71]. Given that the accumulation of oxidized phospholipids is linked to inflammatory diseases like atherosclerosis, OSGIN-1 is positioned as a key mediator of the anti-inflammatory response to OxPAPC-induced oxidative stress.

Likewise, OSGIN-2 has been implicated in countering ROS production and oxidative stress. Defamie et al. (2008) performed RNA-sequencing in patients with initial poor graft function following orthotopic liver transplantation and observed upregulation of *Osgin2* [66]. As ROS accumulation is associated with ischemia-reperfusion injury of transplanted tissues, the upregulation of *Osgin2* suggests that *Osgin2* is a sensor of oxidative stress and ROS accumulation. Keßler et al. (2016) also identified *Osgin2* as a downstream target of miR-199a, a microRNA that is downregulated in hypoxic conditions, reinforcing the assertion that *Osgin2* expression is linked to oxidative stress conditions [67].

### 5.2. Role of OSGIN in Regulating Cell Proliferation

Although neither Goupil et al. (2024) nor subsequent studies directly relate the FMO activity of OSGIN-1 to its physiological role in mediating oxidative stress, Goupil et al. proposes a cellular function for OSGIN-1 as a cytokinetic regulator. In a previous study, Goupil et al. (2017) investigated regulators of cytoplasmic bridge stability in primordial germ cells (PGCs), necessary for recruiting the cellular machinery for abscission during late cytokinesis [48]. Using RNAi-mediated depletion, they found that OSGN-1, the *C. elegans* ortholog of OSGIN-1, is essential for the accumulation of ANI-2, a short anillin protein involved in actomyosin scaffolding at the PGC cytoplasmic bridge [48]. Based on this result, Goupil et al. (2017) hypothesized that OSGN-1 acts upstream of RhoA, a GTPase that recruits and activates actomyosin contractility regulators, to promote localization of ANI-2 to the intracellular cytoplasmic bridge. Goupil et al. (2024) observed a similar phenotype between OSGN-1-depleted and RhoA-depleted *C. elegans* PGCs and surmised that OSGN-1 is a cytokinetic regulator. To determine if OSGN-1 regulates RhoA activity, Goupil et al. (2024) employed live cell imaging using a GFP-bound anillin probe (GFP::AHPH) and performed RNAi-mediated depletion of OSGN-1, resulting in a significant 50% reduction of GFP::AHPH fluorescence during cell division and a lack of detectable fluorescence at the PGC intracellular bridge in late cytokinesis [23]. Goupil et al. (2024) further supported their hypothesis that OSGN-1 regulates RhoA binding at the PGC intercellular bridge by using confocal time-lapse imaging and immunofluorescence of GFP-bound OSGN-1 in HeLa cells to reveal its colocalization with RhoA at the cell midbody during late telophase. While Goupil et al. (2024) does not directly implicate the role of OSGN-1—or its OSGIN-1 functional ortholog—in regulating cytokinesis to ROS production, ROS accumulation is associated with the activation of RhoA. However, the mechanism, and whether it is direct or indirect, is not yet understood [72,73,74].

As Goupil et al. (2024) was unable to conclude that OSGIN-2 is a functional ortholog of OSGIN-1, it could not be concluded that OSGIN-2 regulates RhoA activity [23]. However, OSGIN-2 has been implicated in regulating cell proliferation in other functional studies. For instance, in gastric cancer cells, knockdown of *Osgin2* inhibited tumor cell proliferation and increased the number of cells stuck between the G2/M phases of the cell cycle [68]. Furthermore, Shuai et al. (2022) identified *Osgin2* as a negative regulator of osteogenesis in osteoporotic jawbone bone marrow mesenchymal stem cells (BMSCs) subjected to oxidative stress by regulating the retinoic acid-related orphan receptor alpha (RORα)/bone sialoprotein-osteocalcin (BSP-OCN) signaling pathway [69].

### 5.3. Role of OSGIN in Mediating Apoptosis

Another proposed physiological function of the OSGIN proteins is a mediator of apoptosis. This function was first suggested when overexpression of *Osgin1* in Chang liver cells resulted in higher rates of cell death [60]. Interestingly, the 38 kDa OSGIN-1 isoform originally isolated by Huynh et al. (2001) was more potent at inducing cell death than the 52 kDa isoform, leading Ong et al. (2007) to suggest that the cell death function of OSGIN-1 may be encoded within its N-terminal region. *Osgin1* overexpression also resulted in apoptosis of U2OS cells [53]. Yao et al. performed immunostaining of OSGIN-1 after apoptosis-inducing Cl-amidine treatment and observed localization to the mitochondria, resulting in altered mitochondria morphology and the release of cytochrome c, an activator of the cellular apoptotic function. Later work by Liu et al. (2014) supports the role of OSGIN-1 in apoptosis as OSGIN-1 knockdown resulted in a significant decrease in the apoptotic index of cisplatin-treated cells [52]. Finally, Yuan et al. (2021) used an RNA binding protein immunoprecipitation assay to identify *Osgin1* as a downstream target gene of methyltransferase-like 3 (METTL3), an RNA methylase that performs the M^6^A mRNA methylation modification [61]. METTL3 was upregulated by PM2.5, a component of air pollution capable of inducing genetic or epigenetic variations in airway epithelial cells including upregulation of apoptosis. Thus, Yuan et al. (2021) concluded that M^6^A modification of OSGIN-1 by METTL3 was a response to PM2.5-induced airway epithelial cell injury by inducing apoptosis.

While the connection between *Osgin2* and apoptosis has not been elucidated to date, expression of *Osgin2* was upregulated following activation of PGC-1, a protein involved in maintaining mitochondrial biogenesis [70]. Thus, while direct interaction between OSGIN-2 and the mitochondria has not yet been elucidated, Raharijaona et al. (2009) suggest *Osgin2* may be involved in regulating mitochondrial function similar to its *Osgin1* homolog.

### 5.4. Role of OSGIN in Mediating Ferroptosis

Similar to its proposed physiological function of regulating apoptosis, OSGIN-1 has also been implicated in ferroptosis. Ferroptosis is a form of regulated, nonapoptotic cell death driven by iron-dependent phospholipid peroxidation [75]. Ferroptotic death occurs when phospholipid peroxidases (PLOOHs), a subcategory of ROS, accumulate and disrupt the integrity of the plasma membrane. To prevent the accumulation of PLOOHs, enzymes involved in mitigating ROS and lipid peroxidation are expressed. OSGIN-1 was first implicated in this ferroptosis response by Jia et al. (2024) as they measured upregulation of OSGIN-1 after treating human pancreatic ductal adenocarcinoma cells (PDACs) with ferroptosis inducers [56]. Subsequently, Jia et al. (2024) observed that knockout of either OSGIN-1 or NFE2-like BZIP transcription factor 2 (NFE2L2) promoted ferroptosis in PDAC, but re-expression of OSGIN-1 in NFE2L2-deficient cells rescued cellular ferroptosis resistance. This suggested that OSGIN-1 upregulation in ferroptosis is mediated by the NFE2L2 transcription factor, a conclusion that was supported by a ChIP assay that showed direct binding of NFE2L2 to the OSGIN-1 promoter following treatment with ferroptosis inducers, but not the doxorubicin tumor-suppressive positive control. The association between OSGIN-1 and ferroptosis is further supported by Deng et al. (2025), who observed upregulation of *Osgin1* mRNA and OSGIN-1 protein expression in ovarian cancer cells treated with erastin, a treatment known to induce ferroptosis [57]. However, cells treated with Fer-1, an antioxidant that inhibits ferroptosis, before erastin demonstrated no change in OSGIN-1 expression. These studies further support the role of OSGIN-1 in regulating accumulation of ROS and thereby mediating ferroptosis, although the exact mechanism by which OSGIN-1 mediates ferroptosis remains unclear.

### 5.5. Role of OSGIN in Mediating Autophagy

OSGIN-1 also plays a multifaceted role in regulating autophagy across a variety of cellular stress conditions. *Osgin1* was upregulated in vitro in response to an autophagy-inducing cigarette smoke extract treatment; however, expression of two autophagy markers—MAP1LC3B and SQSTM1—was enhanced following *Osgin1* knockdown in smoking/stress conditions [63]. Of note, two gene markers for autophagy induction—microtubule-associated protein 1 light chain 3 beta (MAP1LC3B) and sequestosome 1 (SQSTM1)—expression were not enhanced following *Osgin1* knockdown in normal conditions. Zhang et al. (2020) reported that overexpression and siRNA knockdown of *Osgin1* increased and impaired the autophagic response in human breast cancer MCF-7 cells, respectively. Furthermore, fractionation and immunoprecipitation assays performed by Zhang et al. (2020) revealed significant interaction between OSGIN-1 and transcription factor EB (also known as TFEB), a regulator of autophagy and lysosome-related genes [54]. Khoi et al. (2022) propose that *Osgin1* expression is regulated by X-box binding protein 1 (XBP1) as part of the palmitic acid (PA)-induced lipotoxicity response in the endoplasmic reticulum of human endothelial cells [64]. Upregulation of *Osgin1* counteracts the effects of lipotoxicity by promoting cell migration to maintain the autophagy response [64]. Zheng et al. (2022) identified *Osgin1* as a downstream target of FXR—a ligand-activated transcription factor involved in inflammatory responses—in a pathway to induce autophagy in exocrine pancreas cells [65]. Satta et al. (2023) implicated both *Osgin1* and *Osgin2* in the autophagic activity of human coronary artery endothelial cells (HCAECs) [29]. Overexpression of each *Osgin* gene induced upregulation of proteostasis genes HSP70 and BAG3, resulting in the accumulation of autophagic vesicles and upregulation of genes in the HSP70/BAG3-controlled chaperone-mediated autophagy pathway [29]. Collectively, these findings indicate that *Osgin1* is an essential mediator linking cellular stress to the autophagy response.

### 5.6. Regulation of Osgin Expression

How *Osgin* gene expression is regulated remains an area of active investigation. Virtually nothing is known regarding the regulation of *Osgin2* expression. For *Osgin1* expression, the p53 and nuclear factor E2-related factor (Nrf2) transcription factors have been identified as mediators of *Osgin* expression in a variety of tissues, stress conditions, and physiological contexts. However, there is disagreement among various studies as to whether the regulation of *Osgin* is p53- or Nrf2-dependent.

Several studies have concluded that OSGIN-1 activity is mediated by p53. For instance, Yao et al. (2008) postulated that *Osgin1* mediates apoptosis in a p53-dependent mechanism [53]. First, Yao et al. (2008) used RT-PCR to compare *Osgin1* expression in cell lines with the wild-type and a mutant, inactive form of p53 and only observed upregulation of *Osgin1* in cells with functional p53. Subsequently, Yao et al. (2008) used annexin V staining to observe an increase in apoptotic cells following overexpression of *Osgin1*, correlating the apoptotic function of *Osgin1* to its regulation by p53. Furthermore, Yao et al. (2008) identified a putative p53 binding site at −720 bp in the *Osgin1* promoter and observed increased p53 binding at this site following doxorubicin treatment, suggesting that p53 regulates the apoptotic response of *Osgin1* to DNA damage [53]. This work was supported by Hu et al. (2012), who used immunostaining of U2OS cells to observe colocalization of p53 and OSGIN-1 to the mitochondria following DNA damage [45]. Additionally, Hu et al. (2012) performed co-immunoprecipitation and pull-down assays to determine that p53 and OSGIN-1 undergo direct protein–protein interactions. Zhang et al. (2020) also implicated p53 in mediating *Osgin1* expression in autophagy as a decrease in autophagy was observed after *Osgin1* knockdown following DNA damage by etoposide treatment [54]. Zhang et al. only observed increased *Osgin1* expression following etoposide treatment in cells with wild-type p53, not in p53-deficient cells, prompting their conclusion that *Osgin1* is activated in response to cellular stress or DNA damage in a p53-dependent manner.

Despite the conclusions drawn by Yao et al. (2008), Hu et al. (2012), and Zhang et al. (2020), other studies suggest that OSGIN is regulated in a p53-independent manner. Liu et al. (2014), for example, observed localization of OSGIN-1 to the mitochondria of PLC-8024 cells with a p53 loss-of-function mutation [52]. Additionally, Jia et al. (2024) explored whether the TP53 pathway controls the upregulation of OSGIN-1 during ferroptosis [56]. The researchers did not observe a change in OSGIN-1 upregulation in p53-knockout PDAC following treatment with ferroptosis inducers, promoting the conclusion that p53 does not regulate OSGIN-1 in ferroptosis. However, knockout of the NFE2L2 transcription factor in PDAC cells inhibited OSGIN-1 upregulation after ferroptosis induction, suggesting that NFE2L2, not p53, regulates OSGIN-1 expression in ferroptosis. Further exploring whether p53 regulates the physiological functions of OSGIN-1, Liu et al. (2014) did not observe a significant correlation between p53 mutations and OSGIN-1 expression in hepatocellular carcinoma tissue samples, indicating that p53 is involved in, but not necessary for, the pro-apoptotic function of OSGIN-1 [52]. Brennan et al. (2017) also challenged the p53-dependent mechanism of OSGIN-1 in apoptosis, as siRNA knockdown of p53 in human astrocytes had no effect on the expression of *Osgin1* mRNA transcripts [58]. Instead, Brennan et al. (2017) proposed that *Osgin1* transcription is regulated by Nrf2 in human astrocytes, whereas p53 is a downstream target of Nrf2-regulated *Osgin1*. They supported this assertion with immunofluorescence microscopy experiments in which monomethyl fumarate (MMF), an activator of the Nrf2 signaling pathway, significantly induced p53 translocation to the nucleus [58]. However, p53 was unable to translocate to the nucleus following siRNA knockdown of *Osgin1*, prompting Brennan et al. (2017) to suggest that OSGIN-1 may regulate p53-mediated transcription of oxidative stress response genes through the Nrf2 signaling pathway.

Nrf2 is a redox-sensitive transcription factor involved in the oxidative stress response. In a recent review, Baird and Yamamoto (2020) characterized the Nrf2–KEAP1 (Kelch-like ECH-associated protein 1) pathway that provides a protective response to oxidative stress: In homeostatic conditions, KEAP1 represses the expression of cytosolic Nrf2 by forming a KEAP1/CUL3/RBX1 E3 ubiquitin ligase complex, ubiquitinating Nrf2 and targeting it for proteasomal degradation. However, in response to oxidative stress, KEAP1 is oxidized by Nrf2 inducers on cysteine residue 151, inactivating its E3 ubiquitin ligase activity [76]. Once it has escaped ubiquitination, Nrf2 can translocate to the nucleus and promote transcription of antioxidant genes. Many antioxidant genes contain an antioxidant response element (ARE) sequence in their promoter to which Nrf2 binds to promote transcription [61]. Yet, the only study to characterize the promoter of *Osgin1*, Ong et al. (2004a), did not identify this sequence [34]. Experimental evidence has demonstrated that the *Osgin* genes are transcriptionally regulated by Nrf2 [27,29,58,77,78], but the molecular mechanism of this modulation, and whether the mechanism is ARE-dependent, remains a matter of continued investigation.

The Nrf2 signaling pathway has been implicated in the response of OSGIN-1 to phospholipid oxidation. For example, Li et al. (2007) observed translocation of Nrf2 to the nucleus of HCAECs following OxPAPC induction, a treatment known to induce *Osgin1* expression [20]. They also observed upregulation of OSGIN-1 expression following treatment with MG115, a proteasome inhibitor that enhances Nrf2 nuclear accumulation [20]. Conversely, Li et al. observed an 86% decrease in OSGIN-1 expression following siRNA-mediated knockdown of Nrf2 in MG115-treated cells. Overall, these results suggest that nuclear translocation of Nrf2 promotes *Osgin1* transcription. Li et al. (2007) identified PEIPC, one of three primary active components of OxPAPC, as the stimulator of *Osgin1* expression. Further supporting this finding, Yan et al. (2014) observed increased *Osgin1* expression in a dose-dependent manner following epoxyisoprostane E2 treatment, a fatty acid at the *sn-2* position of PEIPC, until siRNA knockdown of Nrf2 nullified this upregulation [70]. Collectively, these findings underscore Nrf2 as a central regulator of OSGIN-1 expression in response to phospholipid oxidation, illustrating its essential role in cellular stress responses.

Conflicting findings between Yamashita et al. (2024) and Brennan et al. (2017) underscore the ambiguity regarding the role of Nrf2 in mediating the apoptotic effects of *Osgin1*. Yamashita et al. (2024) investigated the impact of methylmercury, a known neurotoxin, on apoptosis in C17.2 mouse neural stem cells and found that methylmercury treatment led to significant upregulation of *Osgin1* [58,62]. This increase in *Osgin1* mRNA and OSGIN-1 protein levels was countered by actinomycin D, a transcription inhibitor, prompting Yamashita et al. (2024) to conclude that methylmercury induces *Osgin1* transcription. Yamashita et al. (2024) also eliminated the increase in *Osgin1* expression following siRNA knockdown of Nrf2 in methylmercury-treated cells and concluded that the Nrf2 signaling pathway regulates *Osgin1* expression. Additionally, transfecting C17.2 cells with an *Osgin1* overexpression plasmid resulted in a significant increase in cleaved caspase-3, an enzyme implicated in apoptotic induction, implying that *Osgin1* upregulation promotes apoptosis [62]. As a result, Yamashita et al. (2024) concluded that methylmercury activates the Nrf2 pathway to upregulate *Osgin1*, enhancing apoptosis in C17.2 cells. The results from Brennan et al. (2017) contradict this by showing that, while Nrf2 regulates *Osgin1* expression, Nrf2-mediated *Osgin1* did not influence apoptosis in human astrocytes [53]. In the presence of MMF, siRNA knockdown of p53 and Nrf2 significantly induced apoptosis, whereas *Osgin1* knockdown had no effect on apoptosis [58]. This discrepancy suggests that, despite the agreement on Nrf2’s role in regulating *Osgin1*, the effect of Nrf2-mediated *Osgin1* expression on apoptosis remains unresolved.

In summary, while there is a clear role of the OSGIN-1 protein in apoptosis, autophagy, and cell proliferation in response to oxidative stress, the underlying mechanisms are complex and involve multiple regulatory pathways. While some studies, namely Yamashita et al. (2024) and Brennan et al. (2017) or Hu et al. (2012) and Liu et al. (2014), present seemingly contradictory findings, further research is needed to elucidate the specific mechanisms of OSGIN regulation across different cell types and stress conditions. Additionally, Brennan et al. (2017) suggested that distinct OSGIN isoforms might be differentially regulated, highlighting a potential area for further investigation [58]. Given the established roles of p53 and Nrf2 in oxidative stress responses and their apparent involvement in OSGIN regulation, additional studies are required to clarify the contexts in which each regulatory mechanism operates.

## 6. Expression of *Osgin* Genes

Analysis of the spatiotemporal expression patterns of the *Osgin* genes in invertebrate and vertebrate species offers an important avenue to understanding their physiological role. Unfortunately, there are minimal data detailing the expression of *Osgin* genes in the current literature. The majority of available data have been provided through RNA-sequencing (RNA-seq), with only a limited number of spatial in situ hybridization (ISH) or immunocytochemistry (ICC) analyses that explore tissue-specific *Osgin* expression. Below, we review the current *Osgin* expression data using available sources of mRNA and protein expression data in published articles, as well as gene expression and organism-specific expression databases. RNA-seq expression levels will be quantified using the following terminology: low (0.5–10 TPM), moderate (11–1000 TPM), or high (>1000 TPM), when appropriate. More detailed expression data are available in Supplementary Tables S6–S10.

### 6.1. Osgin Gene Expression in Invertebrates

The current analysis of *Osgin* expression in invertebrates is limited to few organisms, including *C. elegans*, *Strongylocentrotus purpuratus* (purple sea urchin), *Culex quinquefasciatus* (southern house mosquito), *Aedes aegypti* (yellow fever mosquito), and *Biomphalaria glabrata* (freshwater snail). To date, the *Osgin* genes have not been identified in *D. melanogaster* and *Musca domestica* (the common house fly). Although the only studies to characterize *Osgin* expression in invertebrates have been from RNA-seq analyses, these studies show that *Osgin* genes display broad temporal and spatial expression.

In *C. elegans*, RNA-seq studies have detected *Osgn1* expression from early embryonic stages throughout adulthood (Supplementary Table S5). Low levels of *Osgn1* expression have been observed in the pharyngeal muscle, intestines, germline cells, and hypodermal seam of *C. elegans* embryos and continue into larvae stages [79]. The highest level of *Osgn1* expression in *C. elegans* occurs in the early larvae stages, particularly in the developing nervous system. Notably, *Osgn1* is also expressed in the dauer larval stage, an alternative developmental stage in which larvae arrest to resist oxidative stress and anoxia [80]. Similar expression patterns continue into the young adult and adult stages.

While expression data of *Osgin1* are less available in other invertebrate species, it maintains similar spatiotemporal expression patterns. In *S. purpuratu (sea urchin)*, RNA-seq data show that *Osgin1* is expressed from cleavage into the early pluteus stage after the larva has hatched [81]. In *C. quinquefasciatus (mosquito), Osgin1* expression is detected from the larval stage, post-eclosion, and into adulthood. Specifically, expression is observed in the antenna and hindlegs of adults. Tissue-specific RNA-seq data in adult *I. scapularis* shows low *Osgin1* expression in the synganglion [82].

*Osgin2* displays a broad temporal expression pattern, predominantly localized to the sex tissues, sensory organs, and digestive system [82]. In *Leptotromibidium pallidum* (mite)*, Osgin2* expression is detected in both the larval and adult stages. Similarly, *A. stephensi* shows *Osgin2* expression from embryo to adult stages, with moderate expression observed in the ovaries, fat body, and digestive and excretory systems of adult females. Observed expression patterns in *A. arabiensis*, *A. albiminus*, and *A. aegypti* (mosquito species) further demonstrate expression of *Osgin2* in sex tissues. Broader *Osgin2* expression has been detected in adult *B. glabrata* (snail), particularly in the albumin gland, kidney, heart, CNS, ovaries, testis, and digestive system.

### 6.2. Osgin Gene Expression During Vertebrate Development

*Osgin1* expression data in early vertebrate development, from oocytes to blastula stages, consist solely of RNA-seq and microarray studies in *D. rerio*, *X. laevis*, *X. tropicalis*, and *M. musculus* (Supplementary Table S5). Prior to fertilization, moderate expression of *Osgin1* has been detected in *M. musculus* oocytes [83]. Following fertilization, expression of *Osgin1* and the ortholog *Osgn1* was detected in *M. musculus* and *D. rerio*, respectively, during the cleavage and blastula stages. However, *Osgin1* expression was not observed during the early development of *Xenopus* embryos [84]. 

In later development, from gastrulation to the postnatal period, more extensive *Osgin1* expression data are available in *H. sapiens*, *M. musculus*, *O. cuniculus*, *D. rerio*, *X. laevis*, and *X. tropicalis* using RNA-seq (Supplementary Table S5). In *M. musculus*, *D. rerio*, and *H. sapiens*, additional spatial in situ hybridization (ISH) and microarray data are available. *Osgin1* expression data are limited in non-mammalian vertebrates as tissue-specific in situ data are only available in *D. rerio*. Employing ISH, *D. rerio* displays expression of *Osgn1* from gastrulation through larval stages. During gastrulation and organogenesis, *Osgn1* is primarily expressed in the CNS, epidermis, periderm, eye, and pronephric duct, while during the postnatal stage, expression is localized to mouth, anus, and intestine [85]. Global RNA-seq data collected in *D. rerio* show *Osgn1* expression from cleavage onward, displaying earlier expression than identified in the ISH data (Supplementary Table S5). In *X. laevis* and *X. tropicalis*, RNA-seq data reveal that *Osgin1* is expressed from early gastrulation through organogenesis [84]. However, tissue-specific in situ expression data are currently unavailable for *Xenopus* during development, offering limited spatial comparison between the non-mammalian vertebrates. 

In mammalian vertebrates, RNA-seq data do not reveal a conserved *Osgin1* expression pattern during later development. However, comparing spatial data across species demonstrates stronger similarity in *Osgin1* expression across species in the developing CNS, liver, kidneys, and reproductive organs. In *M. musculus*, RNA-seq studies have detected moderate *Osgin1* expression in the CNS, liver, intestine, forelimb and hindlimb buds, heart, and reproductive organs between gastrulation and postnatal stages (Supplementary Table S5). While spatial expression analyses predominantly support this pattern of expression, there is some discrepancy between the ISH and microarray data. Of note, while the ISH studies in *M. musculus* detect moderate *Osgin1* expression in the CNS, expression is not observed in the liver, unlike RNA-seq and microarray data [86]. In *H. sapiens*, RNA-seq data detect low-to-moderate *Osgin1* expression in the lungs and ganglionic eminence of developing embryos. While these data alone do not indicate much similarity in expression with *M. musculus*, additional microarray studies detect *Osgin1* expression in the liver, amniotic fluid, metanephros, and testis of *H. sapiens* embryos [83]. To date, no *Osgin1* expression data have been collected in *R. norvegicus* during development. While further work is needed to address the inconsistency between RNA-seq and spatial expression analyses in *H. sapiens* and *M. musculus*, the available data support some commonalities in *Osgin1* expression across mammalian vertebrates. 

*Osgin2* expression data in the early development of vertebrates are available for *X. laevis*, *X. tropicalis*, *M. musculus*, *S. scrofa*, and *D. rerio* using RNA-seq and microarray studies (Supplementary Table S6). Pre-fertilization, low expression of *Osgin2* has been detected in *X. laevis* and *S. scrofa* oocytes but has not been detected in *X. tropicalis* oocytes [83]. Following fertilization, expression of *Osgin2* has been detected in *X. laevis*, *X. tropicalis*, and *M. musculus* from the cleavage through blastula stages. 

In later development, *Osgin2* expression data are widely available through RNA-seq analyses in *D. rerio*, *H. sapiens*, *M. musculus*, *O. cuniculus*, *X. laevis*, and *X. tropicalis*. In situ hybridization has been performed in *D. rerio* and *M. musculus*. In non-mammalian vertebrates, *Osgin2* is expressed in the eye, although temporal expression patterns conflict between fish and amphibians. In *D. rerio*, ISH and RNA-seq reveal *Osgin2* expression in the eye at the pharyngula stage during organogenesis and the hatching stage. However, RNA-seq data of *X. laevis* and *X. tropicalis* show that *Osgin2* is expressed earlier from gastrula to organogenesis, although no spatial data are available for comparison. 

Unlike *Osgin1*, which exhibits variable expression patterns between mammalian vertebrates, *Osgin2* shows consistent patterns of expression, predominantly in the CNS, heart, and sex tissues. During gastrulation, tissue-specific RNA-seq data localize *Osgin2* expression to the mesodermal cells, epiblast, and ectoplacental cone in *M. musculus* embryos. During early organogenesis, both ISH and RNA-seq analyses highlight emerging expression in the heart, CNS, limb buds, and yolk sac. Additional microarray data reveal low-level expression in the respiratory primordium and developing sensory systems, such as the otic placode, otic vesicle, and optic fissure. In *H. sapiens* and *O. cuniculus,* RNA-seq data support primary *Osgin2* expression in the heart and CNS. These patterns continue into the middle and late stages of organogenesis, with ISH and RNA-seq data showing expression in the brain, spinal cord, and ganglia of mammalian species. In *M. musculus*, *Osgin2* continues to be expressed in sensory systems such as the cochlea, membranous labyrinth, olfactory bulb, retina, and lens of the eye. RNA-seq and microarray data further detect *Osgin2* expression in the developing mammalian sex tissues, as well as the kidneys and digestive system of *M. musculus*. To date, no *Osgin2* expression data have been collected in *R. norvegicus* during development (Supplementary Table S6).

### 6.3. Osgin Gene Expression in Adult Vertebrate Organisms

Similar to the late stages of embryonic development, *Osgin1* expression in adult vertebrates is primarily localized to the kidney, liver, and testes, with some species showing expression in additional tissues (Supplementary Table S5). Although spatial microarray expression data are available in a few adult organisms, the majority of *Osgin1* expression data in adult vertebrates is derived from RNA-seq studies. In non-mammalian vertebrates, RNA-seq data in *X. laevis* and *X. tropicalis* both detect low levels of *Osgin1* expression in the liver, but otherwise, the data differ between species. Whereas moderate *Osgin1* expression is observed in the mesonephros of adult *X. tropicalis*, expression in *X. laevis* is identified in the intestine, stomach, kidney, pancreas, spleen, and testis [83,84]. RNA-seq data in *D. rerio* display a similar *Osgin1* expression pattern to *X. laevis*, with moderate expression detected in the spleen, intestine, testis, and kidney as well as the heart, granulocytes, pharyngeal gill, and brain [83].

Similar to non-mammalian vertebrates, expression of *Osgin1* in mammalian vertebrates, namely *M. musculus*, *R. norvegicus*, and *H. sapiens*, is consistently detected at moderate levels in the liver, kidneys, intestine, muscle tissue, and genitourinary tissues (Supplementary Table S5). In murine expression studies, microarray data support this pattern of expression, with additional *Osgin1* expression being observed in the granulocytes, adrenal gland, lung, bone marrow, and esophagus [83]. Tissue-specific RNA-seq conducted in adult *R. norvegicus* and *H. sapiens* displays a fairly consistent pattern of *Osgin1* expression. Notably, both *R. norvegicus* and *H. sapiens* demonstrate low levels of *Osgin1* expression in the CNS—specifically, the cerebellum and frontal cortex of the former and the spinal cord of the latter—unlike *M. musculus* [83]. 

*Osgin2* expression in adult vertebrates is primarily expressed in the brain, heart, and sex tissues, as seen in the late stages of embryonic development (Supplementary Table S6). In non-mammalian vertebrates, RNA-seq in *X. laevis* and *X. tropicalis* both show *Osgin2* expression in the brain, heart, testes, liver, and ovary tissues. Similarly, RNA-seq data in *D. rerio* also show *Osgin2* localized to the brain and the head. In mammalian vertebrates, namely *H. sapiens*, *M. musculus*, and *R. norvegicus*, *Osgin2* expression data are primarily available in RNA-seq analyses and show broad expression. Like non-mammalians, mammalian vertebrates show expression in the CNS and sex tissues. Additional RNA-seq and microarray studies in *M. musculus, R. norvegicus,* and *H. sapiens* show further *Osgin2* expression in the digestive system, visual and reproductive systems, immune tissue, and the peripheral nervous systems.

## 7. The Role of *Osgin* in Disease

Since its discovery, the *Osgin* gene family has been implicated in tumorigenesis and disease-induced apoptosis (Table 3) [19,28,39,52,55,60,68,78,87,88]. From RNA-seq and microarray differential expression studies, *Osgin* expression is seen across a wide range of cancer types including brain glioblastomas, epithelial carcinomas, and breast cancer (Supplementary Table S9). However, research has increasingly showcased the roles of *Osgin1* and *Osgin2* in a wider range of pathologies, including neurological conditions and other varied disorders including arthritis, relapsing-remitting multiple sclerosis, sepsis, and COVID-19 infection (Supplementary Table S10). Furthermore, while the current literature primarily compares the baseline expression of *Osgin* between healthy and diseased tissues, there is some, albeit limited, work investigating the underlying pathological mechanisms of the *Osgin* gene family. While there have been some experimental studies that have investigated these pathological mechanisms, most research, as detailed below, involving differential *Osgin* expression is correlational, most often as RNA-seq and microarray studies.

### 7.1. The Role of Osgin in Breast Cancer

In the initial identification of *Osgin1* by Huynh et al. (2001), researchers established the link of the *Osgin* family to tumorigenesis [19]. In an experiment to identify abnormal expression of growth factors in rat mammary epithelial cells, upregulation of *Osgin1* was seen throughout the progression of pregnancy and lactation. Also, in situ hybridization showed *Osgin1* mRNA transcripts localized in mammary gland tissue, and *Osgin1* expression was correlated with a reduction in new cell formation. Building on previous studies linking augmented breast cancer risk to older age pregnancies [89], Huynh et al. (2001) extended their investigation to human breast cancer cell lines to determine if dysregulation of *Osgin1* expression was critical for the proliferation of breast cancer cells. Differential expression between human breast cancer cell lines and healthy breast tissue was assessed with the Northern blot analysis of cell line poly(A) RNA [19]. While some *Osgin1* expression was observed in the cells transfected with human breast cancer cell lines, these cells exhibited much lower expression compared with healthy tissue. Finally, increased expression was examined through transfection of *Osgin1* cDNA into MCF-7 breast cancer cells, with reduced tumor proliferation being measured in both in vitro and in vivo lactating rat models. As the results from Huynh et al. (2001) suggest that *Osgin1* may be implicated in growth inhibition of cancerous cells in breast tissue, subsequent research has continued to characterize the regulatory role played by *Osgin1* in tumorigenesis, especially in breast cancer.

The mechanistic pathway of *Osgin1* in breast cancer was further explored with chorionic gonadotropin (CG)-mediated reduction in mammary carcinogenesis. Ong et al. (2004b) isolated three mRNA isoforms of the *R. norvegicus* homolog of *Osgin1* [28]. CG treatment had previously been associated with protection against mammary carcinoma during pregnancy [90]. Through in situ hybridization of CG treated differentiated secretory epithelial cells, Ong et al. (2004b) showed upregulation of these isoforms following CG treatment in mammary glands compared with untreated cells [28]. In addition, transfection of the overexpressed *Osgin1* homolog protein in rat liver cells induced apoptosis, suggesting a possible link from CG treatment to mammary carcinoma destruction via upregulation of *Osgin1*. While this does not demonstrate that *Osgin1* is the downstream agent of CG, it suggests that *Osgin1* is involved in the pathway of CG’s protection of breast tissue.

*Osgin1* has also been implicated in PI3K/Akt/Nrf2 pathways for responding to oxidative stress in breast cancer tumor reduction. Tsai et al. (2017) found that docosahexaenoic acid (DHA) induces the expression of *Osgin1* in MCF-7 breast cancer cells, which in turn induces apoptosis through the release of cytochrome C from the mitochondria with increased co-expression of p53 [78]. Downregulation of PI3K/Akt/Nrf2 pathways all downregulated *Osgin1* expression and its induction of apoptotic cell markers, overall supporting the potential use of DHA in inducing apoptosis in breast cancer through induction of *Osgin1* in these pathways. Tsai et al. (2021) later built on this by extending DHA cellular effects via induction of *Osgin1* in AMPK-mediated autophagy pathways [91]. Separate DHA treatment and overexpression of *Osgin1* in MCF-7 cells resulted in similar upregulation of p-AMPKαT172, supporting another possible pathway for *Osgin1*’s impact within breast cancer tissue.

Contrary to the Nrf2-mediated tumor suppression pathway of *Osgin1* presented in Tsai et al. (2017), Bottoni et al. (2024) instead found that triple-negative breast cancer (TNBC) cells can exploit this pathway in order to prevent cell death via cytoprotective autophagy [87]. While exploring the overexpression of the cystine/glutamate antiporter SLC7A11/xCT in mesenchymal stem-like subtype (MSL) TNBC cells, the researchers found that the increased cytosolic concentration of cystine induces expression of the Nrf2 pathway through cysteinylation and inactivation of KEAP1, a suppressor of Nrf2. In turn, upregulation of SLC7A11/xCT was positively correlated with upregulation of Nrf2’s downstream targets, including *Osgin1*, and negatively correlated with tumor cell apoptosis. Based on past studies, Bottoni et al. proposed that *Osgin1* expression in TNBC cells contributes to tumor promotion by triggering autophagy [87]. The resulting autophagy helps maintain cellular homeostasis in response to stressful conditions, though this conclusion is highly speculative. Even then, this study highlights how *Osgin1* may not be strictly tumor-suppressive as suggested by other papers, with tumors being able to take advantage of the protein’s autophagy pathway for cytoprotective effects.

*Osgin1* also appears to be a part of wider physiological disruptions caused by breast cancer. Cancerous cells often release cytokines and hormones that can result in broader changes throughout an organism, and Hojo et al. (2017) investigated this idea with mouse models of triple-negative breast cancer (4T1). RNA-seq showed changes in the expression of hepatic circadian genes in the liver of mouse cell lines with transplanted mammary tumors [92]. Interestingly, while many genes follow rhythmic expression, only a small set of genes were compromised under 4T1 conditions, including *Osgin1*. Furthermore, qRT-PCR showed that oxidative stress genes coding for ETC subunits were downregulated upon 4T1 transplantation, suggesting a correlation between increased oxidative stress and abnormal *Osgin1* expression. Therefore, Hojo et al. illustrated that *Osgin1* is involved in the broader biological impacts of breast cancer in the liver [92], but the relationship between *Osgin1* and oxidative stress in this pathway was not elucidated.

### 7.2. The Role of Osgin in Hepatocellular Cancer

In addition to breast cancer, the expression of *Osgin1* and its mechanism and hepatocellular cancers have been studied in several papers. Liu et al. (2014) showed that not only is *Osgin1* differentially expressed in hepatocellular carcinomas, but allele variants of the transcript are associated with tumorigenesis [52]. In a comparison of 400 tumor samples from human patients to non-cancerous hepatic tissue, the full transcriptome of tissue samples was assessed to find allele imbalances, and the researchers found a specific increase in an *Osgin1* variant (nt 1494: G-A, codon 438: Arg-His). This mutation was solely seen in the hepatocellular carcinoma samples, so functional assays were performed in tumor xenografts into mice to understand the effect of this mutation. While *Osgin1* normally induces apoptosis following chemotherapy administration, this *Osgin1* variant was less effective at apoptosis induction. This may be due to decreased translocation of the *Osgin1* variant from the nucleus to mitochondria in comparison with normal *Osgin1* transcripts. Liu et al. (2014) also found general downregulation of *Osgin1* in hepatocellular carcinomas, with a reduction of 24.7% in comparison with surrounding healthy tissue, and this was correlated to lower survival times of the donor patients [52]. Furthermore, patients with high expression of the *Osgin1* variant had on average shorter survival spans and the lowest apoptotic rate [52]. Therefore, the role of *Osgin1* in hepatocellular carcinomas may stem from both downregulation and increased expression of less effective mutated transcripts.

The role of 5′ UTRs has also been shown to be essential in the downregulation of *Osgin1* in hepatocellular cancers. Building on their previous work in mammary gland cancers, Ong et al. (2007) extended *Osgin1* analysis to hepatocellular carcinomas. Through immunohistochemistry, OSGIN-1 was found to be at reduced or even undetectable levels in 74 of 92 hepatocellular carcinoma samples included in the study, matching the prior work with mammary gland carcinoma [60]. The amount of OSGIN-1 expression was also inversely correlated to liver cancer stage/progression, indicating *Osgin1* expression as a potential staging biomarker; induced overexpression in transfected Chang liver cells resulted in significantly higher cell death rates, proposing a potential novel cancer treatment method. More interestingly, translational suppression in 5′ UTRs appeared to be key in the reduced expression of *Osgin1* in the live tumor samples. Deletion of the 5′ UTRs significantly rescued *Osgin1* expression in transfected Chang liver cells, with increased expression being seen across all three *Osgin1* cDNA transcripts isolated in Ong et al. (2004a) [39]. From this, the researchers theorized that increased expression of translational suppression proteins under cancerous conditions may potentially be binding to the 5′UTRs to reduce translation of *Osgin1* mRNA; in turn, this limits the role of *Osgin1* in the oxidative stress-induced apoptosis pathway.

### 7.3. The Role of Osgin in Ovarian Cancer

*Osgin1* has been specifically linked to ovarian cancer suppression and the efficacy of chemotherapy-induced ferroptosis. Deng et al. (2025) supplemented previous RNA-seq studies with qRT-PCR and immunohistochemistry (IHC) studies to conclude that OSGIN-1 expression is significantly lower in ovarian cancer tissue compared with non-cancerous ovarian tissue [57]. To elucidate the role of OSGIN-1 in ovarian cancer growth, Deng et al. (2025) overexpressed OSGIN-1 in mice injected with OVCAR3 tumor cells and observed decreased tumor volume compared with controls, suggesting that OSGIN-1 has a tumor-suppressive effect in ovarian tissue. Deng et al. (2025) also implicated OSGIN-1 in the ferroptosis of ovarian cancer cells; while ovarian cancer cells treated with erastin, a molecule known to induce ferroptosis, demonstrated decreased levels of ROS, malondialdehyde (MDA), and iron, knockdown of OSGIN-1 restored these ferroptosis biomarkers to near-baseline levels [57]. To investigate the signaling pathway through which OSGIN-1 regulates ferroptosis in ovarian cancer cells, Deng et al. (2025) searched the BioGRID protein–protein interaction database to identify ataxia-telangiectasia mutated (ATM), a serine/threonine kinase that phosphorylates AMPK to activate the AMPK signaling pathway, as a putative interacting partner of OSGIN-1. Subsequent qRT-PCR and Western blot assays revealed that OSGIN-1 acts as a negative regulator of solute carrier family 2 member 3 (SLC2A3) via the AMPK signaling pathway. Using flow cytometry to identify the expression of markers of cell death, Deng et al. (2025) concluded that, while OSGIN-1 promotes ferroptosis, SLC2A3 inhibits the ferroptosis cell death response in ovarian cancer cells. Thus, Deng et al. (2025) identified the OSGIN-1/SLC2A3/AMPK axis as a putative clinical target for ovarian cancer therapeutics.

One chemotherapy agent that has been reported to induce apoptosis in some liver cancer cell cultures is sorafenib. Sorafenib, a multitarget, multikinase inhibitor, has shown promise in ovarian cancer treatment in phase II clinical trials, but it has failed phase III. This result prompted Deng et al. (2025) to explore the role of the OSGIN-1/SLC2A3/AMPK axis in the effectiveness of sorafenib [57]. First, in comparing the tumor size of mice treated with sorafenib and with and without overexpression of either OSGIN-1 or SLC2A3, Deng et al. (2025) observed the smallest tumor size in OSGIN-1 overexpression mice treated with sorafenib. This prompted the researchers to conclude that OSGIN-1 promotes the antitumor effects of sorafenib. Furthermore, treating mice with sorafenib and an SLC2A3-neutralizing antibody showed a greater antitumor effect than the antibody alone, suggesting that the SLC2A3-neutralizing antibody improved the ferroptosis-inducing effect of sorafenib. Ultimately, the work by Deng et al. (2025) presents future avenues for exploring the role of OSGIN-1 in ovarian cancer tumorigenesis, particularly as a potential therapeutic for enhancing regulated mechanisms of cell death in tumor cells.

### 7.4. The Role of Osgin in Other Cancers

The *Osgin* genes have also been implicated in a variety of other cancer types. Ong et al. (2004a) also investigated the role of *Osgin1* in renal cell carcinoma. Similar to their findings with mammary gland and hepatocellular carcinomas, Western blot and immunohistochemical studies showed low expression or even undetectable levels of *Osgin1* in kidney tumors in comparison with surrounding non-cancerous tissue [39]. The role of *Osgin1* as a tumor suppressor in kidney carcinoma was confirmed, with transfected cells having a high rate of apoptosis. Ong et al. (2004a) proposed several hypotheses for the cause of this downregulation, including point mutations in regulatory sequences, hypermethylation in the promoter region, or loss of heterozygosity (a common trend among other genes on chromosome 16q23.3) [39]. However, their results could not confirm any of these ideas, only suggesting that the disruption of *Osgin1* leads to a lessening of regulation over cell proliferation and differentiation.

Building on the role of *Osgin1* in tumor suppression, additional evidence is found in DNA methylation arrays of World Trade Center terrorist attack survivors. Environmental exposure to a variety of toxic substances, including heavy metals, asbestos, glass fibers, and combusted jet fuel has been linked to pervasive health effects in the survivors of the attacks, and Arslan et al. (2020) genetically investigated the impacts of this in their study. DNA methylation analyses of blood samples from survivors and non-survivors from 2001 showed that *Osgin1* had one of the top two highest methylation differences out of 24 suspected tumor suppressor genes [88]. The methylation rate of CpG sites had an average increase of 54.4%, supporting the link between the downregulation of *Osgin1* and increased risk for tumor growth. While the study does not describe a mechanistic impact, it supports an epigenetic link to carcinogenic environmental exposure to cancer development and potentially other exposure-related disease pathways via reduced expression of *Osgin1* [88].

In contrast to the prior studies highlighting tumor suppression, Xie et al. (2023) instead found evidence of *Osgin1* acting in tumor promotion in non-small cell lung cancer (NSCLC) [55]. Using Western blots and immunohistochemistry to assess expression of *Osgin1* and TUBB3, a protein vital to tubulin depolymerization, Xie et al. (2023) observed high upregulation of *Osgin1* in NSCLC tumors. Higher expression levels also correlated with increased tumor size and reduced survival rates. Furthermore, knockdown of *Osgin1* and TUBB3 reversed NSCLC tumor progression, and *Osgin1* knockdown additionally restored the effect of the anti-cancer drug Gefitinib in tumor suppression [55]. Mechanistically, Xie et al. (2023) found that *Osgin1* promotes the phosphorylation of TUBB3, activating a tubulin depolymerization pathway that may explain this unique role in tumor progression. This suggests that *Osgin1* is involved in multiple distinct cellular pathways, with its role in tumor suppression or progression being dependent upon these pathways across cancer types.

In comparison with *Osgin1*, much less research has been performed elucidating the mechanistic role of *Osgin2* in cancer. However, *Osgin2* genes have been utilized in creating prognostic models for colorectal cancer survival spans [93]. Oxidative stress is a widely known risk factor related to cancer progression, with ROS-induced DNA damage acting as one model for tumorigenesis. Chen et al. (2022) therefore decided to utilize the expression patterns of oxidative stress-related genes (OXEGs) to create a prognostic genetic tool for patients with colorectal carcinomas. Following the sequencing of 177 colorectal cancer tumor samples from current patients, Chen et al. (2022) found fourteen OXEGs that were consistently differentially expressed in cancerous and non-cancerous tissues [93]. A risk model was constructed using a combination of multivariate and univariate Cox regression analysis as well as LASSO regression analysis, relating differential expression of OXEGs to the potential for tumor progression. *Osgin2* was one of these OXEGs used in the prognostic model, with consistent upregulation in many of the colorectal cancer tumors. While Chen et al. (2022) admitted that the underlying function of *Osgin2* is still relatively unknown, its consistent expression patterning could be used in developing more nuanced cancer prognoses.

Similar upregulation of *Osgin2* was observed bioinformatically in other cancers. Using The Cancer Genome Atlas Stomach Adenocarcinoma (TCGA-STAD) data collection, Wang et al. (2023) observed upregulation of *Osgin2* in glioblastomas, pancreatic cancers, and all stages of gastric cancer [68]. Further characterization of the role of *Osgin2* in gastric cancer revealed that gastric carcinoma cells and tissues contain high levels of OSGIN-2 [66]. Moreover, higher levels of *Osgin2* expression are correlated with poorer prognosis. Knockout of *Osgin2* in gastric cancer cells via siRNA transfection led to cell cycle arrest and inhibited tumor proliferation [68].

Another study correlated the expression of *Osgin2* with the prognostic outcome of patients with soft tissue sarcomas [67]. Keßler et al. (2016) analyzed expression of hypoxia-associated miR-199a-5p, a micro-RNA, in ninety-six soft tissue sarcoma tissue samples. Using a luciferase reporter assay, Keßler et al. (2016) concluded that co-transfection of SAOS-2 cells with miR-199a-5p induces 26.2% downregulation of *Osgin2* due to an miR-199a-5p binding site in the 3′ UTR [67]. miR-199a-5p transfection also decreased the expression of OSGIN-2 proteins under hypoxic conditions [67]. Moreover, a Kaplan–Meier analysis associated low miR-199a-5p expression with decreased patient survival time, although this association was not statistically significant [67]. Thus, while this study provides insight into the link between miRNA regulation of stress genes implicated in tumorigenesis in a hypoxic environment, the mechanism relating *Osgin2* to tumor hypoxia is not yet understood.

### 7.5. The Role of Osgin in Other Conditions and Diseases

While the majority of research implicating the *Osgin* genes in disease focuses mainly on their roles in cancers, *Osgin1* and *Osgin2* have also been abnormally expressed in a wide variety of conditions and disorders. For example, Yildrim, Tola, and Dağ (2020) have implicated underexpression of *Osgin1* in polycystic ovary syndrome (PCOS), an endocrine disorder that can lead to infertility and widespread metabolic disorder [94]. Serum levels of *Osgin1* were correlated with a variety of anthropometric measurements in patients suffering from PCOS. *Osgin1* was found to be consistently downregulated across patients, with Yildrim, Tola, and Dağ, positioning *Osgin1* expression as a negative diagnostic indicator for PCOS [94]. Increased expression was often found to reduce the risk of cardiovascular disease and metabolic syndrome disorders, both common secondary effects of PCOS. While they admit that the small sample size of the study limits any conclusions they can make, the researchers hypothesize that *Osgin1* is vital in maintaining homeostasis in metabolic processes for patients diagnosed with the disorder.

Multiple studies have also investigated the upregulation of *Osgin1* in response to smoking. Wang et al. (2017) obtained airway epithelial cells from healthy smokers and nonsmokers, testing the expression levels of *Osgin1*. *Osgin1* expression was upregulated in smokers in both the large and small airway epithelium, and this was confirmed across PCR microarray data [63]. Also, to determine if the *Osgin1* overexpression was due to oxidative stress or DNA damage, airway epithelial cells were treated directly with H_2_O_2_, a common source of ROS. Following treatment, the observed *Osgin1* upregulation indicated that overexpression was due to the oxidative stress processes. When exposed to cigarette smoke, human airway epithelium cells also showed upregulation of *Osgin1* in microarray and RNA-seq data [63]. These results indicate that oxidative stress caused by exposure to cigarette smoke causes the overexpression of the functional *Osgin1* gene and protein. Building on this idea, Satta et al. (2023) similarly showed that both *Osgin1* and *Osgin2* were upregulated in in vitro human coronary artery endothelial cells and in vivo mouse aortas following exposure to cigarette smoke [29]. When *Osgin1* and *Osgin2* were overexpressed, Satta et al. found increased endothelial cell detachment and cell cycle arrest at the S-phase; furthermore, detachment was found to result in *Osgin*-mediated dysregulation of the molecular chaperone Hsp70, demonstrating novel treatment targets for long-term smokers.

Cigarette smoke exposure’s upregulation of *Osgin1* has also been used to investigate fibrosis in COPD. Tang et al. (2024) exposed human bronchial epithelial cells (HBECs) to cigarette smoke extract in their exploration of fine (2.5 μm or less in diameter) particulate matter’s (PM_2.5_) link to COPD pathology [95]. *Osgin1* upregulation was first confirmed in HBECs treated with PM_2.5_, and this was also associated with increased fibrosis biomarkers within the cells. Mechanistically, an experimental knockdown of *Osgin1* resulted in lower fibrosis biomarkers, indicating that *Osgin1* acts as an upstream agent within the COPD fibrosis pathway. In addition, by using miRNA targeting software, Tang et al. were able to identify a miRNA, miR-654–5p, that inhibits translation of *Osgin1*, blocking its downstream effects related to fibrosis [95]. A Western blot analysis supported this targeting as well as showed *Osgin1*’s role in inducing autophagy in HBECs that contributes to overall lung fibrosis. Therefore, Tang et al. showcase a novel pathway for treatment of COPD fibrosis, outlining a new regulatory axis in which PM_2.5_ disinhibits *Osgin1* expression by blocking the actions of miR-654–5p, resulting in dysregulated autophagy and increased fibrosis.

*Osgin2* has also been implicated in the development of osteoporosis in rat jawbone models. The loss of bone marrow stromal cells (BMSCs) that make bone cells is associated with aging and loss of estrogen levels, resulting in less protection against oxidative stress [69]. *Osgin2* is associated with bone marrow loss, specifically in Nijmegen breakage syndrome and retinitis pigmentosa type 62. Under normal conditions, the receptor RORα is able to regulate the differentiation of BMSCs to promote osteogenesis via transcriptional upregulation of osteogenic genes OCN and BSP. Shuai et al. (2022) hoped to elucidate the relation of *Osgin2* expression on RORα and ultimately give a better understanding of the pathophysiology of osteoporosis. OCN and BSP levels were first compared via qRT-PCR in rat osteoporitic jawbone BMSCs under the addition of hydrogen peroxide (a common reactive oxygen species). Higher oxidative stress was negatively correlated with the osteopromotive genes, and the effect was lessened with the addition of antioxidants [69]. Shuai et al. (2022) noted that *Osgin2* transcription was augmented at these sites following the addition of hydrogen peroxide [69]. Following the knockdown of *Osgin2* in the rat models, a reversal was seen towards osteogenesis, with higher levels of OCN and BSP following hydrogen peroxide addition in comparison with the first trial. Therefore, upregulation of *Osgin2* measured in osteoporosis appears to be implicated in the destructive properties of reactive oxygen species; Shuai et al. (2022) hypothesized that *Osgin2* is a negative indicator of osteogenesis when BMSCs are exposed to increased oxidative stress [69].

**Table 3:** *Osgin* pathological expression in correlational studies. Differential *Osgin* expression comparing pathological with nonpathological cells and tissues in various cancers and conditions. Pathologies were selected to highlight based on comparable studies focusing on the same pathology in the same tissue type. See Supplementary Materials for a full list of pathologies. An up-facing arrow signifies upregulation in comparison with nonpathological tissue, while a down-facing arrow signifies downregulation. Expression data sourced from [96,97,98,99,100,101,102].

Pathology	Method	*Osgin1* Expression	*Osgin2* Expression
Breast Cancer	RNA-seq	↓	↑
Cholangiocarcinoma	RNA-seq	↓	↑
Hepatocellular Carcinoma	RNA-seq	↑	↑
Lung Carcinoma	RNA-seq/ microarray	↑	↓
Osteosarcoma	RNA-seq/ microarray	↓	↑
Glioma	RNA-seq/ microarray	↓	↑
Nevus Sebaceous of Jadassohn	RNA-seq	↑	↓
Sepsis	RNA-seq	↑	↓
Graft vs. Host Disease	RNA-seq	↓	↑

The majority of research into the *Osgin* gene family’s role in disease has been performed in correlational studies, largely through RNA-seq or microarrays that compare expression in pathological with nonpathological tissues. Overall, correlational experiments illustrate that *Osgin1* and *Osgin2* are differentially expressed across a wide range of pathologies, including many types of cancer [91,92,95], infections [98,99], chronic conditions [100,101,102], and various other disorders [97,103,104]. Some general trends regarding expression can be found through these studies. *Osgin1* and *Osgin2* tend to have opposite expression patterns, and *Osgin1* is found to be more often downregulated in cancerous tissues while *Osgin2* is more often upregulated. However, the results of differential expression studies can be inconsistent, indicating that expression patterns may be more dependent on the specific biological pathway the gene is involved in as well as variations among different cancer and organism types. This emphasizes the need for a mechanistic understanding of *Osgin1* and *Osgin2* so that the reasoning behind upregulation or downregulation can be better understood. Differential expression studies currently dominate research on *Osgin* expression in disease because the gene is often implicated in large transcriptomic or proteomic studies, so there needs to be a heightened motivation in disease research on the specific mechanistic actions of these seemingly ubiquitous genes.

## 8. Conclusions

While there has been significant progress in understanding the role of the *Osgin* genes, overall, this remains an understudied gene family, particularly in light of its physiological importance. Following the discoveries of *Osgin1* and *Osgin2* by Huynh et al. (2001) and Tauchi et al. (1999), respectively, ongoing research has augmented our understanding of these genes with regard to evolutionary history, structure, and function [19,21]. On a larger scale, analysis of RNA-seq studies that identified differentially expressed *Osgin* genes have provided greater insight into the expression of *Osgin* in pathological and nonpathological tissues, leading to advances in elucidating the underlying roles of the *Osgin* genes in disease-promoting or -suppressing pathways. Despite a growing body of research characterizing the *Osgin* genes in recent years, there are still many unanswered questions surrounding the *Osgin* genes, particularly *Osgin2.* In this review of the current literature on the *Osgin* gene family, we identify several areas that require further investigation for fully understanding these genes: analysis of the evolutionary history of the genes, elucidation of the OSGIN protein structure, perturbation studies to better elucidate function, positioning of *Osgin* genes in signaling and gene networks, in-depth and diverse analysis of expression patterns across vertebrate and invertebrate eukarya, and a mechanistic understanding of *Osgin* expression under normal and pathological cellular conditions.

The current research is still lacking detailed or complete investigation into the phylogenetic and evolutionary history of the *Osgin* gene family. Although some initial BLASTn analyses were performed in the seminal set of papers by Huynh et al. (2001) and Ong et al. (2004b), as well as more recently in Satta et al. (2023), the characterization of *Osgin* conservation is limited to extensive conservation of the genes within vertebrate eukarya and between the *Osgin1* and *Osgin2* paralogs [19,28,29]. More recently, Goupil et al. (2024), although not a dedicated phylogenetic analysis, represents a promising example of an attempt to probe this gene family’s evolutionary conservation in an invertebrate organism, revealing a high degree of conservation within a non-chordate species [23]. Available genome data also indicate conservation of OSGIN proteins across animals, fungi, and protists, an exciting avenue of investigation. The current state of *Osgin* literature calls for a need for greater inquiry into the phylogenetic history and conservation of this gene family as a whole, with an emphasis on invertebrate, fungal, and protist species. Further functional analyses and more extensive estimations of the phylogenetic history of OSGIN using multiple sequence alignment software and diverse tree construction models will give greater insight into the conservation of this ancient gene family.

With respect to the biochemical functions of the OSGIN proteins, OSGIN-1 was recently identified as a flavin-containing monooxygenase (FMO) that requires the binding of FAD and NAD(P)H cofactors [23]. However, FMOs are a broad class of enzymes with a range of structural and catalytic properties. Further categorization of OSGIN into its FMO subclass can be accomplished with spectrophotometric analysis of the FAD and NAD(P)H cofactors while OSGIN-1 is enzymatically active. This categorization will provide insight into the type of reactions OSGIN-1 can catalyze, modulation of OSGIN-1 activity, and perhaps the substrate of the OSGIN-1 enzyme. Additionally, bioinformatic analysis of the *Osgin1* gene have pinpointed several functional domains within the OSGIN-1 structure, such as the Pyr_redox_2 and TrkA flavoprotein domains and the YpdA buffering compound [40,45]. Techniques to elucidate the structure of OSGIN-1 such as NMR, X-ray crystallography, cryo-electron microscopy, and mutagenesis studies are necessary to verify these domains and identify other potential domains or interacting partners. Moreover, RNAi- or CRISPR/Cas9-mediated knockout experiments can be used to evaluate the functionality of each domain within OSGIN-1.

However, despite the advances in characterizing OSGIN-1, the structure and biochemical function of OSGIN-2 is drastically understudied. Although Goupil et al. (2024) concluded that OSGIN-2 is not a functional ortholog of *C. elegans* OSGN-1, the DTNB-methimazole assay will determine if OSGIN-2, like OSGIN-1, is an FMO [23]. Furthermore, bioinformatic and in vitro characterization of the structure of OSGIN-2 is necessary to elucidate the functionality of OSGIN-2 and its conservation with its OSGIN-1 homolog.

The localization of *Osgin* in metabolic gene and signaling pathways, including numerous oxidative stress response pathways, remains unclear. In particular, *Osgin1* has been characterized as a transcriptional target of Nrf2 and p53 across varying tissues and stress conditions, but the mechanism of these interactions is unclear. Putative binding sites have been identified for both p53 [53] and Nrf2 [62] upstream from the *Osgin1* transcriptional start site, but further functional analysis, such as an electrophoretic mobility shift assay, must be performed to establish the function of these binding sites. Furthermore, RNAi- or CRISPR/Cas9-mediated deletion of the p53 and Nrf2 binding sites may elucidate the necessity of each transcription factor in regulating the physiological function of the OSGIN proteins. Finally, additional co-immunoprecipitation and experiments and binding studies should be performed to identify the downstream targets of the OSGIN proteins in oxidative stress response pathways.

The regulation of *Osgin* gene expression is not well understood. While two promoter regions—P1 and P2—have been identified for *Osgin1* [39], further characterization of promoter and enhancer regions via ChIP-Seq experiments for both *Osgin* genes could provide critical information regarding protein binding sites and epigenetic modification at the transcriptional level. For example, many oxidative stress response genes have an ARE sequence identified in their promoter [76], and the only paper to characterize the promoter of *Osgin1*, Ong et al. (2004a), did not identify this element. More extensive analysis of the promoters of both *Osgin* genes to identify whether ARE is present will provide further insight into the role and regulation of *Osgin* in the oxidative stress pathway.

Surprisingly, at the RNA processing level, there is still no consensus in the current literature regarding the number of introns and exons in *Osgin1* or *Osgin2*. Moreover, while both genes undergo alternative splicing to produce multiple isoforms of each *Osgin* homolog in vitro, the functional differences and differential expression of each isoform have not been characterized. Although the current literature has primarily characterized the 52 kDa and 56.7 kDa isoforms of OSGIN-1 and OSGIN-2, respectively, the experimentation of Brennan et al. (2017) suggested that different OSGIN isoforms may have different physiological functions [58]. Therefore, identifying which OSGIN isoforms regulate each in vitro physiological function and, once identified, performing overexpression and knockout studies of each isoform is necessary to determine the functional differences between each OSGIN isoform. Finally, at the post-translational level, few putative protein–protein interacting partners have been characterized in vivo or in vitro for OSGIN-1, and none have been characterized for OSGIN-2. To supplement the existing bioinformatic proposed interacting partners, crosslinking, pull-down, co-immunoprecipitation assays are needed to elucidate the intracellular interactions of the OSGIN proteins.

RNA-sequencing has been the primary technique used to analyze expression patterns during organismal development. These experiments show that *Osgin1* primarily localizes to the CNS, kidney, testes, and liver and *Osgin2* localizes to the brain, heart, and sex tissues in vertebrates and invertebrates. While RNA-sequencing data are available in many vertebrates during embryogenesis, there are currently only two in situ hybridization analyses of *Osgin1* and *Osgin2* in *M. musculus* and *D. rerio* that characterize expression during development, limiting spatial comparison between organisms and other techniques. Even in *M. musculus*, there are discrepancies between the ISH, RNA-seq, and microarray data that cannot be resolved without additional in situ analyses. Notably, the *M. musculus* RNA-seq study quantifies *Osgin1* expression in the CNS, kidney, liver, and testis during organogenesis, whereas the ISH study by Diez-Roux et al. (2011) characterizes expression only in the CNS [86]. Similarly, expression patterns cannot be compared between non-mammalian vertebrates because ISH data are not available in *X. laevis* and *X. tropicalis*. Invertebrate expression data of *Osgin* are limited because there are few RNA-seq studies. Thus, the use of additional experimental techniques such as in situ hybridization, immunocytochemistry, qRT-PCR, single-cell RNA sequencing, or spatial transcriptomics can provide higher specificity and a more comprehensive overview of baseline expression patterns.

In a majority of the current *Osgin* literature, *Osgin* has been studied in disease states. However, the aim of these studies was to identify up- or downregulated genes in disease, so little is known regarding the mechanism of *Osgin* in pathogenesis. While some studies have partially elucidated *Osgin*’s mechanistic role in pathways like p-AMPKαT172 autophagy [91] or protein–protein interactions with TUBB3 [55], a large share of research related to *Osgin1* or *Osgin2* are wide-reaching cancer expression analyses, reporting differential expression across a wide variety of genes with varied and potentially unrelated physiological function. While the existing set of differential expression studies allows for initial patterns of expression to emerge across diseases and emphasizes how *Osgin1* and *Osgin2* are highly relevant in many pathologies, a mechanistic understanding of *Osgin* in disease states is lacking in the current literature. Therefore, greater emphasis must be placed on deciphering the roles of *Osgin1* and *Osgin2* within larger biological pathways—both in normal development and disease states—to understand the role of *Osgin* in pathogenesis. This can be accomplished by performing *Osgin* overexpression and knockdown experiments to further understand the understudied functions of the OSGIN proteins and connect this function to the expression of *Osgin* in disease. Finally, in addition to exploring the use of *Osgin* in possible breakthroughs in cancerous or chronic conditions, greater emphasis should be placed on understanding other aspects of *Osgin1* and *Osgin2* beyond their roles in disease.

Due to the apparent link between inappropriate *Osgin* expression and pathogenesis, the *Osgin* gene family exhibits potential for therapeutic applications. The current literature shows that *Osgin1* and *Osgin2* display differential expression in a variety of cancers and diseases, though this is, in most cases, the extent of clinical knowledge. To increase the biomedical relevance of *Osgin* research, effort must be focused on extending exploration into tangible therapeutic targets and constructs. This potential for therapeutic benefit has already been shown with the use of SCL2A3-neutralizing antibodies for ovarian cancer treatment in Deng et al. (2025) [57]. With a better understanding of the role of OSGIN proteins in the pathogenicity of signaling pathways, rather than simply an assessment of differential expression, viable therapeutic targets could be selected for effective use in a medical context. However, barriers to treatment development should also be addressed. The role of *Osgin* as a tumor suppressor or tumor promoter appears to potentially be organism- and pathology-specific, suggesting corroboration of findings in animal models to human cell lines is necessary to ensure cross-species effectiveness for new therapeutic approaches. Finally, *Osgin* genes have also been investigated as prognostic markers for cancer [36,60,67,93]. Additional prognostic data can allow clinicians to create more nuanced treatment plans for their patients; however, further research is necessary to determine how *Osgin* expression may change throughout the different stages of cancer development.

In conclusion, the current research on the *Osgin* gene family provides promising future directions yet has many areas in need of further characterization. Most notably, the literature on *Osgin2* is far less extensive than *Osgin1* as *Osgin2* has primarily been identified as a putative inducer of cell proliferation in disease [68]. As expression data reveal differing spatiotemporal expression patterns between *Osgin1* and *Osgin2*, it is likely that the genes are functionally different and require independent investigation. Further research to distinguish the structure, function, and conservation of *Osgin2* from *Osgin1* are necessary to lapse the disproportionate amount of research between the genes. However, more research is clearly needed on the *Osgin* gene family as a whole. While research heavily focuses on disease states, there needs to be a more comprehensive analysis of expression, structure–function relationships, phylogeny, regulatory mechanisms, and functional perturbation experiments to understand the role of this ancient and physiologically important gene family during development as well as in homeostasis of the mature organism.

## 9. Limitations

While this review aims to provide a comprehensive summary of the current *Osgin* literature, several limitations must be acknowledged. Firstly, the review is inherently limited by the availability of *Osgin* data and the literature. Secondly, our methodology and exclusion criteria dictate that we did not include studies not available in English, which may omit valuable information and introduce the potential for selection bias. Finally, our review of *Osgin* expression data is limited to secondary data on major databases, restricting the scope of the review. These limitations should be considered when interpreting the results of this review.

## Figures and Tables

**Table 1:** Physiological function of *Osgin1*. Functional assays characterizing the physiological function of *Osgin1*. Studies selected to highlight the cell/tissue cultures or in vivo studies in which *Osgin1* physiological function was investigated. See Supplementary Materials for the complete list of functional assays.

Physiological Function	Cell, Tissue, or Organism	Source
Countering Oxidative Stress	Human aortic endothelial cells	[20]
	Human myeloid leukemia U937 cell line	[59]
Regulating Cytokinesis	*C. elegans*	[23]
Mediating Apoptosis	Human osteosarcoma U2OS cells Human breast cancer MCF-7 cells Human hepatocellular carcinoma tissue samples Human spinal cord astrocytes Human A549 lung adenocarcinoma cells Human bronchial epithelial cells Mouse C17.2 neural stem cells	[60] [53] [52] [58] [61] [62]
Mediating Ferroptosis	Human pancreatic ductal adenocarcinoma cells	[56]
	Human SKOV3 and ES-2 ovarian cancer cell lines	[57]
Mediating Autophagy	Human primary airway basal stem cells	[63]
	Human breast cancer MCF-7 cells	[59]
	Human umbilical vein endothelial cells	[64]
	Human pancreatic tissue	[65]
	Human coronary artery endothelial cells	[29]

**Table 2:** Physiological function of *Osgin2*. Functional assays characterizing the physiological function of *Osgin2*. Studies selected to highlight the cell/tissue cultures or in vivo studies in which *Osgin2* physiological function was investigated. See Supplementary Materials for the complete list of functional assays.

Physiological Function	Cell or Tissue Type	Source
Countering Oxidative Stress	Human liver tissue biopsy sample	[66]
	Human soft tissue sarcoma sample	[67]
Regulating Cell Proliferation	NUGC3 and HGC27 gastric cancer cells	[68]
Regulating Osteogenesis	Osteoporotic jawbone bone marrow mesenchymal stem cells	[69]
Maintaining Mitochondrial Biogenesis	XTC.UC1 and B-CPAP human thyroid cell lines	[70]
Mediating Autophagy	Human coronary artery endothelial cells	[29]

## Data Availability

Not applicable.

## Supplementary Materials

The following supporting information can be downloaded at: https://www.mdpi.com/article/10.3390/biom15030409/s1, Table S1: Putative interacting partners of OSGIN-1 in *Homo sapiens*, *Rattus norvegicus*, and *Mus musculus*, identified by the STRING protein–protein interaction database [43,105]; Table S2: Putative interacting partners of OSGIN-2 in *Homo sapiens*, *Rattus norvegicus*, and *Mus musculus*, identified by the STRING protein–protein interaction database [43,105]; Table S3: Resulting phenotypes from *Osgin1* gene expression perturbations [19,20,23,28,29,39,45,52,53,54,58,60,61,62,63,64,65,77,78,91]; Table S4: Resulting phenotypes from *Osgin2* gene expression perturbations [23,29,67,68,69,70]; Table S5: RNA-seq data for *Osgin1* from gametogenesis through adulthood [79,81,82,83,84,96,106,107,108,109,110,111]; Table S6: RNA-seq data for *Osgin2* from gametogenesis through adulthood [79,81,82,83,84,96,106,107,108,109,110,111]; Table S7: Raw expression data of *Osgin* during development [84,86,97,106,107,108,109,111,112,113,114,115,116,117,118,119,120,121,122,123,124,125,126,127,128,129,130,131,132,133,134,135,136,137,138,139,140,141,142,143,144,145,146,147,148,149,150,151,152,153,154,155,156,157]; Table S8: Raw *Osgin* expression data of vertebrates across several species [41,84,97,110,111,113,114,117,123,156,158,159,160,161,162,163,164,165]; Table S9: Differential *Osgin* expression comparing pathological to nonpathological cells and tissues (unless otherwise specified) in cancers and precancerous conditions [97,98,99,100,101,166,167,168,169,170,171,172,173]; Table S10: Differential *Osgin* expression comparing pathological to nonpathological cells and tissues (unless otherwise specified) in diseases other than cancers [97,102,103,104,174,175,176,177,178,179,180,181,182,183,184,185,186].

## Abbreviations

The following abbreviations are used in this manuscript:

AMPK	Adenosine monophosphate-activated protein kinase
ARE	Antioxidant response element
ATM	Ataxia-telangiectasia mutated
BAC	Bacterial artificial chromosome
BDGI	Bone marrow stromal cell-derived growth inhibitor
BYA	Billion years ago
C8orf1	Chromosome 8 open reading frame 1
CDD	Conserved Domain Database
CG	Chorionic gonadotropin
COPD	Chronic obstructive pulmonary disease
DHA	Docosahexaenoic acid
DTNB	5,5′-dithiobis(2-nitrobenzoate)
ER	Endoplasmic reticulum
ETC	Electron transport chain
FAD	Flavin adenine dinucleotide
FMO	Flavin-containing monooxygenase
GCLM	Glutamate-cysteine ligase modifier subunit
GSH	Glutathione
HBEC	Human bronchial epithelial cell
hT41	Human testis 4.1-kb transcript
ICC	Immunocytochemistry
IHC	Immunohistochemistry
IK2	Ikaros factor 2
ISH	In situ hybridization
MDA	Malondialdehyde
MEGA	Molecular Evolutionary Genetics Analysis
MGI	Mouse Genome Informatics
ML	Maximum likelihood
MSL	Mesenchymal stem-like
NAD(P)H	Nicotinamide adenine dinucleotide phosphate hydrogen
NBS	Nijmegen breakage syndrome
NCBI	National Center for Biotechnology Information
NGF	Nerve growth factor
NMR	Nuclear magnetic resonance
NSCLC	Non-small cell lung cancer
OKL38	Ovary, kidney, and liver protein 38
ORF	Open reading frame
*Osgin*	Oxidative stress-induced growth inhibitor
*Osgin1*	Oxidative stress-induced growth inhibitor 1
*Osgin2*	Oxidative stress-induced growth inhibitor 2
OXEG	Oxidative stress-related gene
OxPAPC	Oxidized 1-palmitoyl-2-arachidonoyl-sn-glycero3-phosphorylcholine
P1	Promoter 1
P2	Promoter 2
PCOS	Polycystic ovary syndrome
PCR	Polymerase chain reaction
PDAC	Pancreatic ductal adenocarcinoma cells
PLOOH	Phospholipid peroxidases
PM_2.5_	Fine (diameter of 2.5 μm or less) particulate matter
qRT-PCR	Quantitative reverse transcription polymerase chain reaction
RACE	Rapid amplification of cDNA ends
RGD	Rat Genome Database
RNA-seq	RNA sequencing
ROS	Reactive oxygen species
RPKM	Reads per kilobase million
SAR	Stramenopiles, Alveolates, and Rhizaria
SLC2A3	Solute carrier family 2 member 3
TCGA-STAD	The Cancer Genome Atlas Stomach Adenocarcinoma
TNB	Nitro-5-thiobenzoate
TNBC	Triple-negative breast cancer
TPE	Transcripts per embryo
TPM	Transcripts per million
UTR	Untranslated region
