# Supplementary material for: The Osgin Gene Family: Underexplored Yet Essential Mediators of Oxidative Stress
Source: Biomolecules. 2025 Mar 13;15(3):409. doi: 10.3390/biom15030409 (PMC11940746; doi:10.3390/biom15030409)
Supplement: Supplementary file 1 [file biomolecules-15-00409-s001.zip › biomolecules-3482307-supplementary.pdf]

***Osgin* Supplementary Materials**

Supplementary Accession Numbers: Xenopus_tropicalis_osgin1, XP_002935177.1; Xenopus_tropicalis_osgin2 XP_012820265.2; Xenopus_laevis_osgin1.L XP_018113295.1; Xenopus_laevis_osgin2.L XP_018123603.1; Mus_musculus_Osgin2, ENSRNOP00060000818.1; Mus_musculus_Osgin1, ENSMUSP00215038011.1; Rattus norvegicus_Osgin2, ENSRNOP00060000818.1; Rattus_norvegicus_Osgin1, ENSRNOP00060034558.1; Papio_anubis_OSGIN2, XP_031525831.1; Papio_anubis_OSGIN1, XP_009195210.1; Macaca_mulatta_OSGIN2, NP_001247699.1; Macaca_mulatta_OSGIN1, XP_014982184.2; Chlorocebus_sabaeus_OSGIN2, XP_007999231.1; Chlorocebus_sabaeus_OSGIN1, XP_007992390.2; Homo_sapiens_OSGIN2, ENSP05155077337.1; Homo_sapiens_OSGIN1, ENSP05155068619.1; Equus_caballus_OSGIN1, XP_023493155.1; Equus_caballus_OSGIN2, XP_005613246.2; Sus_scrofa_OSGIN2, XP_001925962.1; Sus_scrofa_OSGIN1, XP_020949398.1; Bos_taurus_OSGIN2, XP_002692895.1; Bos_taurus_OSGIN1, XP_024834383.1; Varanus_komodoensis_OSGIN2, XP_044280435.1; Varanus_komodoensis_OSGIN1, XP_044290084; Gallus_gallus_OSGIN2, XP_040551741; Gallus_gallus_OSGIN1, XP_414073.2; Danio_rerio_osgn1, NP_956480.1; Danio_rerio_osgin2, XP_017206984.1; Thunnus_albacares_OSGIN2, XP_044215198.1; Thunnus_albacares_OSGIN1, XP_044206015.1; Salmo_salar_osgin2, XP_045574938.1 ; Salmo_salar_osgin1, XP_045547340.1; Caenorhabditis_elegans_osgn1, CDH93477.1; Caenorhabditis_briggsae_hypothetical_protein, UMM30818.1; Caenorhabditis_remanei_Oxidative_stress-induced_growth_inhibitor, EFO90357.1; Sturnus_vulgaris_OSGIN2, XP_014726253.1; Sturnus_vulgaris_OSGIN1, XP_014736308.1; Nematostella_vectensis_oxidative stress-induced growth inhibitor 1, XP_032238790.2; Acropora_digitifera_oxidative stress-induced growth inhibitor 1-like, XP_015778258.1; Aplysia_californica_oxidative stress-induced growth inhibitor 2, XP_005109786.1; Mercenaria_mercenaria_oxidative stress-induced growth inhibitor 2-like, XP_045192302.1; Haliotis_rufescens_oxidative stress-induced growth inhibitor 2-like, XP_046368151.1; Scophthalmus maximus_oxidative stress-induced growth inhibitor 2, XP_035481536.1; Daphnia_magna_oxidative stress-induced growth inhibitor 1-like, XP_045035634.1; Daphnia pulex_oxidative stress-induced growth inhibitor 1-like, XP_046442706.1; Pollicipes_pollicipes_oxidative stress-induced growth inhibitor 1-like, XP_037085440.1; Dermacentor_silvarum_oxidative stress-induced growth inhibitor 1-like isoform X1, XP_049525354.1; Apis laboriosa_oxidative stress-induced growth inhibitor 1-like, XP_043801825.1; Bombus_pyrosoma_oxidative stress-induced growth inhibitor 1-like isoform X1, XP_043580928.1; Vespa_mandarinia_oxidative stress-induced growth inhibitor 1-like isoform X1, XP_035725345.1; Ischnura_elegans_oxidative stress-induced growth inhibitor 1-like, XP_046386313.1; Patiria_miniata_oxidative stress-induced growth inhibitor 1-like, XP_038069512.1; Strongylocentrotus_purpuratus_oxidative stress-induced

growth inhibitor 1, XP_794627.3; Xiphophorus_hellerii_osgin2, XP_032410370.1; Xiphophorus_helleri_osgn1, XP_032406000.1; Poecilia_latipinna_osgn1, XP_014892131.1; Poecilia_latipinna_osgin2, XP_014911316.1; Aspergillus_bombycis_oxidative stress induced growth inhibitor family member 2, OGM49278.1; Wickerhamiella_sorbophila_Oxidative stress-induced growth inhibitor 1, PRT54095.1; Aspergillus_lentulus_oxidative stress-induced growth inhibitor 2, GFF78793.1; Aspergillus_udagawae_isoform 1 of Oxidative stress-induced growth inhibitor 1, GFF31452.1; Penicillium_chrysogenum_Oxidative stress-induced growth inhibitor, KZN89867.1; Penicillium_digitatum_Oxidative stress-induced growth inhibitor 1, XP_014538563.1; Penicillium subrubescens_Oxidative stress-induced growth inhibitor 1, OKO97845.1; Penicillium_rolfsii_Oxidative stress-induced growth inhibitor 1, GFF78793.1; Penicillium_arizonense_hypothetical protein, OGE47690.1; Symbiodinium_microadriaticum_Oxidative_stress-induced_growth_inhibitor, CAE7371548.1; Symbiodinium_microadriaticum_Oxidative_stress-induced_growth_inhibitor_1, OLQ09364.1; Symbiodinium_sp._KB8_Oxidative_stress-induced_growth_inhibitor, CAE7849348.1; Symbiodinium_sp._CCMP2592_Oxidative_stress-induced_growth_inhibitor, CAE7663915.1; Symbiodinium_sp._CCMP2456_Oxidative_stress-induced_growth_inhibitor, CAE7614348.1; Symbiodinium_sp._CCMP2456_Oxidative_stress-induced_growth_inhibitor, CAE7436152.1; Symbiodinium_necroappetens_Oxidative_stress-induced_growth_inhibitor, CAE7451409.1;

Supplementary Table S1. Putative interacting partners of OSGIN-1 in *Homo sapiens*, *Rattus norvegicus*, and *Mus musculus* identified by STRING protein-protein interaction database [43,105].

**Protein Name**	**Protein Function**	***Homo sapiens*?**	***Rattus norvegicus*?**	***Mus musculus*?**
NAD(P)H dehydrogenase [quinone] 1 (NQO1)	Quinone reductase involved in hydroquinone reactions, detoxification pathways, and prothrombin synthesis	✓	✓	✓
Thioredoxin reductase 1(TXNRD1)	Glutaredoxin and thioredoxin reductase activity; induces actin and tubulin polymerization	✓	✓	✓
Sulfiredoxin-1 (SRXN1)	Participates in oxidative stress resistance by reducing cysteine-sulfinic acid in presence of the peroxiredoxin oxidants PRDX1, PRDX2, PRDX3, and PRDX4	✓	✓	✓

Glutamate-cysteine ligase modifier subunit (GCLM)	Aldo/keto reductase family; involved in sulfur metabolism and glutathione biosynthesis	✓	✓	✓
Ribosomal protein S6 kinase alpha-6 (RPS6KA6)	Serine/threonine-protein kinase; may participate in p53/TP53-dependent signaling	✓		
Calpain-3 (CAPN3)	Calcium-regulated non-lysosomal thiol-protease in peptidase C2 family	✓		
Protein-arginine deiminase type-4 (PADI4)	Catalyzes citrullination and deimination of arginine residues on histones and other proteins; involved in histone regulation and stem cell maintenance	✓		
Leucine-rich repeat, immunoglobulin-like domain and transmembrane domain-containing protein 1 (LRIT1)	Photoreceptor synaptic protein; possible role in phototransduction and synapse formation of cone photoreceptor cells	✓		
Homeobox protein Meis2 (MEIS2)	Forms dimer with HOX or PBX proteins or binds DNA-bound HOX or PBX dimer; involved in transcriptional regulation	✓		
MANSC domain containing 4 (MANSC4)	Enables serine-type endopeptidase inhibitor activity	✓		
Glycolipid transfer protein domain-containing protein 2 (GLTPD2)	Predicted to enable ceramide 1-phosphate binding and transfer activity		✓	✓
StAR-related lipid transfer protein 6 (STARD6)	Involved in intracellular sterol and lipid transport			✓
Maestro heat-like repeat family member 1 (MROH1)	Predicted to be active in the cytoplasm		✓	

Supplementary Table S2. Putative interacting partners of OSGIN-2 in *Homo sapien*, *Rattus norvegicus*, and *Mus musculus* identified by STRING protein-protein interaction database [43,105].

**Protein Name**	**Protein Function**	***Homo sapiens*?**	***Rattus norvegicus*?**	***Mus musculus*?**
2,4-dienoyl-CoA reductase (DECR1)	Enzyme involved in beta-oxidation and unsaturated fatty enoyl-CoA ester metabolism; catalyzes the NADP-dependent reduction of 2.4-dienoyl-CoA to trans-3-enoyl-CoA	✓	✓	✓
Calbindin (CALB1)	Buffers cellular entry of calcium upon stimulation of glutamate receptors	✓	✓	✓
Carbonic anhydrase 7 (CA7)	Alpha-carbonic anhydrase family; catalyzes reversible hydration of carbon dioxide	✓		
Calretinin (CALB2)	Calbinin family; intracellular calcium-binding protein abundant in auditory neurons	✓		
Nibrin (NBN)	Component of the MRN complex which is involved in DNA double-strand break repair, DNA recombination, maintenance of telomere integrity, cell cycle checkpoint control, meiosis and maintenance of chromosome integrity; the complex has single-strand endonuclease activity and double-strand-specific 3’-5’ exonuclease activity	✓		
FERM and PDZ domain containing 3 (FRMPD3)	Signal transduction protein; FERM domain is associated with cytoskeletal proteins; PDZ domain associated with protein-protein interactions	✓	✓	✓

FERM and PDZ domain containing 4 (FRMPD4)	Involved in postsynaptic actin cytoskeletal organization; predicted to enable phosphatidylinositol-4,5-bisphosphate binding activity		✓	
S100 calcium binding protein G (S100G)	Calcium-binding protein associated with buffering of intracellular calcium	✓		
Male-specific lethal 1 homolog (MSL1)	Component of histone acetyltransferase complex involved in acetylation of histone H4 at Lys-16; enhances MSL2 ubiquitin ligase activity	✓		
G-protein coupled receptor 107 (GPR107)	Putative receptor for neuronostatin; induces glucagon in low glucose conditions to regulate blood sugar		✓	✓
G-protein coupled receptor 146 (GPR146)	Regulates cholesterol levels in plasma membrane; associated with adenylate cyclase-inhibiting G protein-coupled receptor signaling pathway	✓	✓	✓
SH3 and cysteine-rich domain-containing protein (STAC)	Promotes expression of the ion channel CACNA1H at the cell membrane		✓	✓
Protein phosphatase 1H (PPM1H)	Dephosphorylates CDKN1B at 'Thr-187' to remove signal for proteasomal degradation		✓	✓
Cytochrome c oxidase assembly factor 3 homolog (COA3)	Component of MITRAC complex which regulates cytochrome c oxidase assembly			✓
Protein lin-52 homolog (LIN52)	Predicted to be involved in regulation of DNA-templated transcription		✓	✓

SH3 domain-containing protein 19 (SH3D19)	Regulates cell morphology and cytoskeletal organization; may be involved in regulating A disintegrin and metalloproteases in signaling of EGFR-ligand shedding; putative involvement in suppression of Ras-induced cellular transformation and Ras-mediated activation of ELK1			✓
RAD18 E3 ubiquitin protein ligase (RAD18)	Predicted to enable Y-form DNA binding activity, identical protein binding activity, and ubiquitin protein ligase binding activity; predicted to be involved in positive regulation of chromosome segregation, post-replication repair, and protein monoubiquitination		✓	

Supplementary Table S3. Resulting phenotypes from *Osgin1* gene expression perturbations [19,20,23,28,29,39,45,52–54,58,60–65,77,78,91]

Perturbation	Cell/Tissue Type	Phenotype	Method	Source
Knockdown of OSGN-1	*C. elegans* primordial germ cells	Decrease in GFP::AHPH fluorescence throughout cell division	RNAi-mediated depletion; time-lapse confocal microscopy	Goupil et al., 2024
RNAi-mediated depletion of OSGN-1	*C. elegans* primordial germ cells	Binucleated PGCs	Confocal microscopy	Goupil et al., 2024
Osgn-1 point mutation ($P^{21}L$)	*C. elegans* primordial germ cells	Binucleated PGCs with regression of PGC intercellular bridge	Confocal microscopy	Goupil et al., 2024

Osgn-1 point mutation ($A^{99}T$)	*C. elegans* primordial germ cells	Regression of PGC intercellular bridge	Confocal microscopy	Goupil et al., 2024
Osgn-1 point mutation ($S^{126}N$)	*C. elegans* primordial germ cells	Binucleated PGCs with regression of PGC intercellular bridge	Confocal microscopy	Goupil et al., 2024<br>Goupil et al., 2024
Osgn-1 point mutation (W^{201}Stop)	*C. elegans* primordial germ cells	Binucleated PGCs with regression of PGC intercellular bridge	Confocal microscopy	Goupil et al., 2024
Osgn-1 point mutation ($T^{275}A$)	*C. elegans* primordial germ cells	Binucleated PGCs with regression of PGC intercellular bridge	Confocal microscopy	Goupil et al., 2024
Osgn-1 point mutation ($P^{303}S$)	*C. elegans* primordial germ cells	Binucleated PGCs with regression of PGC intercellular bridge	Confocal microscopy	Goupil et al., 2024
CRISPR-Cas9-mediated deletion of *Osgin1*	HeLa cells	Increase in multinucleated PGCs	Indirect immunofluorescence	Goupil et al., 2024
Expression of *C. elegans* OSGN-1 in *Osgin1*-depleted cells	HeLa cells	Restoration of mononucleated phenotype	Indirect immunofluorescence	Goupil et al., 2024
Treatment with OxPAPC	Human aortic endothelial cells	Upregulation of *Osgin1*	RT-PCR	Li et al., 2007

Treatment with OxPAPC and N-acetyl cysteine	Human aortic endothelial cells	No change in *Osgin1* expression	RT-PCR	Li et al., 2007
Treatment with tert-Butylhydroquinone	Human aortic endothelial cells	Upregulation of *Osgin1*	RT-PCR	Li et al., 2007
Treatment with MG115 proteasome inhibitor	Human aortic endothelial cells	Upregulation of *Osgin1*	RT-PCR	Li et al., 2007
Treatment with oxLDL-IC	Human U937 cells	Upregulation of *Osgin1*	DNA microarray transcriptome profiling	Hammad et al., 2009
Knockdown of *Osgin1*	HAEC cultures from *H. sapien* heart transplant donors	Upregulation of HMOX1, a regulator of inflammation	siRNA knockdown, RT-qPCR	Romanoski et al., 2011
Knockdown of *Osgin1*	HAEC cultures from *H. sapien* heart transplant donors	Upregulation of IL-8, ATF4, and KLF4, associated with endothelial cell inflammatory response	siRNA knockdown, RT-qPCR	Romanoski et al., 2011
Overexpression of *Osgin1*	A498 Cells	Increased in growth inhibition and cell death	Cell culture and transfection	Ong et al., 2004a
Overexpression of *Osgin1*	Buffalo rat liver cells	Increased cell death	Cell culture and transfection	Ong et al., 2004b
Overexpression of *Osgin1*	Chang liver cells	Increased cell death	Cell culture and transfection	Ong et al., 2007
Deletion of *Osgin1* 5’ UTR	Chang liver cells	Significant increase in translation of *Osgin1*	PCR, cell transfection, Western blot analysis	Ong et al., 2007

Overexpression of *Osgin1*	U2OS cells	Increase in apoptotic cells	Annexin V staining	Yao et al., 2008
Treatment with doxorubicin	MCF-7 cells	Upregulation of *Osgin1*	qRT-PCR	Yao et al., 2008
Treatment with doxorubicin	MCF-7 cells	Increased amount of p53 present at *Osgin1* promoter 2 and 6 hours after treatment	ChIP assay	Yao et al., 2008
siRNA Knockdown of PADI4	U2OS cells	~2.5 fold increase in *Osgin1* expression	qRT-PCR	Yao et al., 2008
Treatment with Cl-amidine	MCF-7 cells	Upregulation of *Osgin1*	qRT-PCR	Yao et al., 2008
Treatment with Cl-amidine or doxorubicin	U2OS cells	Localization of OSGIN-1 to mitochondria	Immunostaining	Yao et al., 2008
Overexpression of *Osgin1*	U2OS cells	Change in mitochondrial morphology and cytochrome c localization	Immunostaining and flow cytometry	Yao et al., 2008
Knockdown of OSGIN-1	LO2 immortalized liver cell line	Increase in cisplatin G150 values from 1.73 µg/mL to 2.81-2.82 µg/mL	shRNA knockdown	Liu et al., 2014
Knockdown of OSGIN-1	Miha immortalized liver cell line	Increase in cisplatin G150 values from 2.0 µg/mL to 3.5-4.32 µg/mL	shRNA knockdown	Liu et al., 2014
Treatment with 2 µg/mL cisplatin in	LO2 immortalized liver cell line	Significant decrease in apoptotic index	Flow cytometry	Liu et al., 2014

OSGIN-1-transfected cells		compared to shRNA-transfected controls		
Treatment with $PM_{2.5}$	Human bronchial epithelial (HBE) cells	Increased mRNA expression of *Osgin1*	RT-qPCR	Yuan et al., 2021
Knockdown of METTL3	Human bronchial epithelial (HBE) cells	Shorter *Osgin1* mRNA half-life	Me-RIP-qPCR	Yuan et al., 2021
Knockdown of *Osgin1*	Human bronchial epithelial (HBE) cells	$PM_{2.5}$-mediated apoptosis and cell cycle arrest partially attenuated	siRNA knockdown; Me-RIP-qPCR	Yuan et al., 2021
Knockdown of *Osgin1*	Human bronchial epithelial (HBE) cells	Decreased conversion of LC3A/B-I to LC3A/B-II; decreased expression of P62 mRNA and protein	siRNA knockdown; Me-RIP-qPCR	Yuan et al., 2021
Treatment with cigarette smoke extract	Human airway epithelial cells	Upregulation of *Osgin1* mRNA and OSGIN-1 protein	TaqMan PCR	Wang et al., 2017
Overexpression of *Osgin1*	Human primary airway basal cells	Upregulation of MAP1LC3B and SQSTM1	TaqMan PCR	Wang et al., 2017
Overexpression of *Osgin1* and treatment with BafA1	Human primary airway basal cells	Enhanced autophagic flux	Lentivirus (Lenti-*Osgin1* plasmid) upregulation; Western blot analysis	Wang et al., 2017

Knockdown of *Osgin1* and treatment with BafA1	Human primary airway basal cells	No consistent effect on autophagic flux	siRNA downregulation; Western blot analysis	Wang et al., 2017
Overexpression of *Osgin1*	Human Breast Cancer MCF-7 Cells	Increased expression of GFP-LC3, an autophagic marker	Immunoblotting	Zhang et al., 2020
Knockdown of *Osgin1*	Human Breast Cancer MCF-7 Cells	Decreased expression of GFP-LC3, an autophagic marker	siRNA-mediated knockdown, Western blot assay	Zhang et al., 2020
Overexpression of *Osgin1*	Human coronary artery epithelial cells	Upregulation of HSP70 and BAG3	RNAseq analysis	Satta et al., 2023
Overexpression of *Osgin1*	Human coronary artery epithelial cells	Reduced cell proliferation; increase in senescence markers	Adenovirus OSGIN-1 overexpression transfection; cell viability assay; Senescence-associated beta-galactosidase assay	Satta et al., 2023
Knockdown of *Osgin1*	Human coronary artery epithelial cells	Inhibition of Nrf2-induced cell detachment	shRNA knockdown; shear stress assay	Satta et al., 2023
Treatment with cigarette smoke	Mouse aorta	Significantly higher OSGIN-1 expression	Immunohistochemical analysis	Satta et al., 2023

Overexpression of *Osgin1*	Human MCF-7 breast cancer cells	Decreased cell proliferation	Western Blot assay	Huynh et al., 2001
Knockdown of *Osgin1*	Primary human umbilical vein endothelial cells	Decreased in LC3 B mRNA expression; decrease in p-eNOS and LC3-II protein expression	siRNA knockdown; qRT-PCR	Khoi et al., 2022
Knockdown of *Osgin1* with PA treatment	Primary human umbilical vein endothelial cells	Suppression of cell migration	siRNA knockdown; wound healing migration assay	Khoi et al., 2022
Knockdown of *Osgin1* with palmitic acid treatment	Primary human umbilical vein endothelial cells	No significant change in ROS generation	siRNA knockdown; intracellular reactive oxygen species assay	Khoi et al., 2022
Treatment with GW4064	Pancreatic exocrine organoid	~13-fold increase in *Osgin1* mRNA levels	RNAseq	Zheng et al., 2022
Treatment with GW4064 with knockdown of FXR	Pancreatic exocrine organoid	No change in *Osgin1* mRNA expression	RNAseq	Zheng et al., 2022
Knockdown of *Osgin1*	Pancreatic exocrine organoid	Increase in serum lipase level and pancreatic tissue edema	shRNA-knockdown	Zheng et al., 2022
Overexpression of *Osgin1* and treatment with GW4064	Pancreatic exocrine organoid	Reduction of autophagic vesicle accumulation compared to GW4064-treated controls	Transmission electron microscopy	Zheng et al., 2022

Treatment with doxorubicin	U2OS cells	Increase in OSGIN-1 expression	Western blot assay	Hu et al., 2012
Treatment with doxorubicin	U2OS cells	Colocalization of OSGIN-1 and p53 to mitochondria	Immunostaining	Hu et al., 2012
Forced OSGIN-1 expression	U2OS cells	Enlarged mitochondria and alteration of cristae structures	Transmission electron microscopy	Hu et al., 2012
Treatment with docosahexaenoic acid	MCF-7 breast cancer cells	Upregulation of *Osgin1*, LC3-II, and SqSTM1/p62	Total RNA isolation and RT-PCR	Tsai et al., 2021
Overexpression of *Osgin1* and treatment with docosahexaenoic acid	MCF-7 breast cancer cells	Increased Bax/Bcl-2 ratio, mitochondrial accumulation of OSGIN-1 and p53, and cytochrome c release	Plasmid vector overexpression; mitochondrial fractionation assay; Western blot analysis	Tsai et al., 2017
Knockdown of Nrf2 with treatment with docosahexaenoic acid	MCF-7 breast cancer cells	No change in *Osgin1* expression	Total RNA isolation and RT-PCR	Tsai et al., 2017
Overexpression of *Osgin1* with docosahexaenoic acid treatment	MCF-7 breast cancer cells	Dose-dependent increase of OSGIN-1 accumulation in mitochondria; increased mitochondrial ROS level	Transfection with OSGIN-1 overexpression plasmid; MitoSOX-based assay	Tsai et al., 2021
Knockdown of *Osgin1* with	MCF-7 breast cancer cells	No change in OSGIN-1 accumulation in	siRNA knockdown;	Tsai et al., 2021

docosahexaenoic acid treatment		mitochondria; no change in mitochondrial ROS level	MitoSOX-based assay	
Treatment with Epoxyisoprostane E2	Human aortic endothelial cells	Dose-dependent increase in OSGIN-1 and HO-1 expression	qRT-PCR	Yan et al., 2014
Treatment with Epoxyisoprostane E2 and Apocynin	Human aortic endothelial cells	No change in OSGIN-1 expression	qRT-PCR	Yan et al., 2014
Treatment with monomethyl fumarate	Human spinal cord astrocytes	Upregulation of *Osgin1*	qRT-PCR	Brennan et al., 2017
Treatment with monomethyl fumarate with siRNA knockdown of Nrf2	Human spinal cord astrocytes	~50% decrease in *Osgin1* expression compared to active Nrf2 control	qRT-PCR	Brennan et al., 2017
Treatment with monomethyl fumarate with siRNA knockdown of *Osgin1*	Human spinal cord astrocytes	No change in Nrf2 expression	qRT-PCR	Brennan et al., 2017
Knockdown of p53	Human spinal cord astrocytes	No change in *Osgin1* expression	siRNA-mediated knockdown, qRT-PCR	Brennan et al., 2017
Knockdown of *Osgin1*	Human spinal cord astrocytes	No change in p53 expression	siRNA-mediated knockdown, qRT-PCR	Brennan et al., 2017
Treatment with methylmercury	Mouse C17.2 neural stem cells	Upregulation of *Osgin1*	qPCR	Yamashita et al., 2024

Treatment with methylmercury and actinomycin D	Mouse C17.2 neural stem cells	No change in *Osgin1* expression	qPCR	Yamashita et al., 2024
Knockdown of Nrf2 following treatment with methylmercury	Mouse C17.2 neural stem cells	No change in *Osgin1* expression	siRNA-mediated knockdown; qPCR	Yamashita et al., 2024
Overexpression of *Osgin1*	Mouse C17.2 neural stem cells	Upregulation of cleaved caspase-3	Transfection with *Osgin1* overexpression plasmid; qPCR	Yamashita et al., 2024

Supplementary Table S4. Resulting phenotypes from *Osgin2* gene expression perturbations [23,29,67–70].

Perturbation	Cell/Tissue Type	Phenotype	Method	Source
Treatment with miR-199a-5p in hypoxic conditions	Human soft tissue sarcoma	Downregulation of *Osgin2*	Western blot analysis	Keßler et al., 2016
Treatment with miR-199a-5p in normoxic conditions	Human soft tissue sarcoma	No change in *Osgin2* expression	Western blot analysis	Keßler et al., 2016
Knockdown of *Osgin2*	Gastric carcinoma cells	Inhibition of tumor cell proliferation; cell cycle arrest	siRNA knockdown; CCK8 experiment	P. Wang et al., 2023
Knockdown of *Osgin2*	Sprague Dawley rat bone marrow stem cell	Increase in jawbone mass of osteoporotic rats; improved new bone deposition	siRNA knockdown; calcein labeling assay	Shuai et al., 2022
Overexpression of *Osgin2*	Human coronary artery epithelial cells	Upregulation of HSP70 and BAG3	RNAseq analysis	Satta et al., 2023

Overexpression of *Osgin2*	Human coronary artery epithelial cells	Reduced cell proliferation; increase in senescence markers	Adenovirus OSGIN-2 overexpression transfection; cell viability assay; Senescence-associated beta-galactosidase assay	Satta et al., 2023
Knockdown of *Osgin2*	Human coronary artery epithelial cells	Inhibition of Nrf2-induced cell detachment	shRNA knockdown; shear stress assay	Satta et al., 2023
Treatment with cigarette smoke	Mouse aorta	Significantly higher OSGIN-2 expression	Immunohistochemical analysis	Satta et al., 2023
Knockdown of PGC-1 related coactivator	Human thyroid cell lines	Upregulation of *Osgin2*	siRNA knockdown; microarray; RT-PCR	Raharijaona et al., 2009
CRISPR-Cas9-mediated deletion of *Osgin2*	HeLa cells	Increase in multinucleated PGCs	Indirect immunofluorescence	Goupil et al., 2024
Expression of *C. elegans* OSGN-1 in *Osgin2*-depleted cells	HeLa cells	Increase in multinucleated PGCs	Indirect immunofluorescence	Goupil et al., 2024

Supplementary Table S5. RNA-Seq data for *Osgin1* from gametogenesis through adulthood. - indicates expression is undetected (<0.5 TPM or <1300 TPE only for *X. tropicalis*). + indicates expression is detected at low levels (0.5 to 10 TPM). ++ indicates expression is detected at moderate levels (11 to 1000 TPM). +++ indicates expression is detected at high levels (>1000 TPM). N/A indicates that expression data is unavailable for the stage [9,81–84,96,106–111]

	Pre-fertilization	Cleavage	Blastula	Gastrula	Early organogenesis	Mid organogenesis	Late organogenesis	Postnatal period	Adults
A. carolinensis	N/A	N/A	N/A	N/A	N/A	N/A	N/A	N/A	++(heart) +(lung, kidney, muscle,

									liver, adrenal tissue, brain, dewlap)
B. taurus	N/A	N/A	N/A	N/A	N/A	N/A	N/A	N/A	++ (kidney, liver, testes)
C. elegans	N/A	++	++	+	+ (developing nervous system)	+	+	++	++
C. lupus familiaris	N/A	N/A	N/A	N/A	N/A	N/A	N/A	N/A	++ (liver, kidney, skin, testis, CNS, pituitary, intestine stomach, adrenal gland, nose, pancreas, retina, granulocyte, olfactory segment of nasal mucosa, thyroid) + (heart, ovary, muscle, lung, spleen, tongue, lymph node,

									urinary bladder, adipose)
C. quinquefasciatus	N/A	N/A	N/A	N/A	N/A	N/A	N/A	++	++ (antennae, hindlegs)
D. rerio	N/A	+	+	++	++	++	++	++ (heart)	++ (kidney, liver, testis, intestine, lung, granulocytes, heart, brain, spinal cord, pharyngeal gill)<br>+ (spleen, stomach, muscle)
H. sapiens	N/A	N/A	N/A	N/A	+ (CNS)	-	+ (lung)	-	++ (liver, testes, kidney, intestine, muscle, stomach, adrenal gland, brain, spinal cord)<br>+(urinary bladder)
M. gallopavo	N/A	N/A	N/A	N/A	N/A	N/A	N/A	++(thymus, gizzard,	++(thymus, gizzard)

								testis, proventriculus, spleen, pancreas, ovary, intestine, heart. cecal tonsil, bursa of fabricius, liver,) +(muscle, brain)	
M. mulatta	N/A	N/A	N/A	N/A	N/A	N/A	N/A	N/A	++ (kidney, heart, liver)
M. musculus	++	++	+	+ (ectoplacental cone)	++ (liver, metanephros, ovary, testes, yolk sac, forelimb and hindlimb buds)	++ (liver, heart, hindbrain, forebrain, limb, neural tube)	++ (kidney, liver, limb, heart, midbrain, stomach, intestine, lung, brain)	++ (kidney, ovary, testes, liver) + (muscular system, digestive system)	++ (kidney, testes, liver, intestine, granulocytes, esophagus, adrenal gland, urinary bladder) + (bronchus, lung, trachea, muscle, seminal vesicle, bone

									marrow, prostate)
O. cuniculus	N/A	N/A	N/A	N/A	+ (ovary, testes)	+ (ovary, testes)	+ (liver, ovary, kidney)	+ (liver, ovary, kidney)	+ (liver, ovary, kidney)
P. anubis	N/A	N/A	N/A	N/A	N/A	N/A	N/A	N/A	++ (kidney, liver)
R. norvegicus	N/A	N/A	N/A	N/A	N/A	N/A	N/A	N/A	++ (kidney, liver, testes, muscle, stomach, esophagus, pancreas)<br>+ (heart, adrenal gland, thyroid gland, adipose tissue, frontal cortex)
S. purpuratus	N/A	++	+	+	+	+	+	+	N/A
S. scrofa	N/A	N/A	N/A	+ (forelimb bud, hindlimb bud)	N/A	N/A	N/A	N/A	++ (muscle, testis, liver, kidney, uterus)

									+ (intestine, heart, granulosa cell, occipital cortex, lung, prefrontal cortex, spleen, hypothalamus, adipose tissue)
X. laevis	-	-	-	-	+	+	+	N/A	++ (intestine, spleen)<br>+ (kidney, liver, stomach, muscle, lung, pancreas, ascending aorta)
X. tropicalis	-	-	-	-	+	+	+	N/A	++(mesonephros)<br>+(liver, testes, stomach

Supplementary Table S6. RNA-Seq Data for *Osgin2* from gametogenesis through adulthood. - indicates expression is undetected (<0.5 TPM or <1300 TPE only for *X. tropicalis*). + indicates expression is detected at low levels (0.5 to 10 TPM). ++ indicates expression is detected at moderate levels (11 to 1000 TPM). +++ indicates expression is detected at high levels (>1000 TPM). N/A indicates that expression data is unavailable for the stage [79,81–84,96,106–111].

	Pre-fertilization	Cleavage	Blastula	Gastrula	Early organogenesis	Mid organogenesis	[68, 70, 71, 72, 73, 86, 100, 101, 102, 103, 104, 105]	Postnatal period	Adults
A. arabiensis<br>*A. albiminus*	N/A	N/A	N/A	N/A	N/A	N/A	N/A	N/A	++ (ovary, oviduct, testis, accessory gland, body segments)
A. stephensi	N/A	++	+	+	+	+	+	+	++ (ovary, midgut, malpighian tubule, fat body)
A. aegypti	N/A	++	++	+	+	+	+	+ (malpighian tubule)	+ (ovary, testes, antennae, legs, brain, malpighian tubule, gut, spermatheca, body segments)
A. carolinensis	N/A	N/A	N/A	N/A	++ (forelimb bud, hindlimb bud, tail, hemipenis)	+	+	+	++ (ovary, dewlap) + (heart, adrenal gland)

B. glabrata	N/A	+	+	+	+	+	+	N/A	++ (kidney, heart, stomach, albumen gland)
C. lupus familiaris	N/A	N/A	N/A	N/A	N/A	N/A	N/A	++ (skin, heart)<br>+ (lung, kidney, liver, lymph node, head)	++ (heart, spinal cord, thyroid, pituitary gland, brain, placenta, hair follicle, testis, adrenal cortex, urinary bladder, spleen)<br>+ (salivary gland, liver, keratinocyte, stomach, thymus, granulocyte, retina, head, kidney intestine, blood, skin, bone marrow, adipose, muscle)

D. rerio	-	-	-	-	+	-	-	+	++ (brain)<br>+ (head)
H. sapiens	N/A	N/A	N/A	N/A	++ (spinal cord, cerebellum, medulla oblongata)	++ (spinal cord, cerebellum, medulla oblongata)	++ (spinal cord, cerebellum, medulla oblongata)	++ (spinal cord, cerebellum, medulla oblongata)	++ (CNS, PNS, leukocytes, bone marrow, pancreas, muscle, uterus, testis, adipose, gallbladder, esophagogastric junction muscularis propria, artery, colon)<br>+ (blood, amygdala, caudate nucleus, anterior cingulate cortex)
L. pallidum	N/A	N/A	N/A	N/A	N/A	N/A	N/A	++	++
M. gallopavo	N/A	N/A	N/A	N/A	N/A	N/A	N/A	++ (testis, brain, ovary, spleen, thymus, pancreas, proventriculus,	++(gonad)<br>+ (spleen)

								intestine) + (liver, cecal tonsil, heart, gizzard, hindlimb stylopod)	
M. mulatta	N/A	N/A	N/A	N/A	N/A	N/A	N/A	N/A	+ (brain, digestive system, heart)
M. musculus	-	+	N/A	++ (epiblast, ectoplacental cone)	++ (heart, tail, forelimb bud, hindlimb bud, brain) + (yolk-sac)	++ (limbs, large intestine, brain, forelimb and hindlimb bud, heart, liver)	++ (CNS, digestive system, kidney, retina, limbs, midbrain, hindbrain, neural tube, stomach, lung, intestine, heart) + (liver)	++ (CNS and retina)	++ (primary visual cortex, testis, thymus, esophagus, retina, brain, lung, sperm, skin, lens of camera-type eye, ovary, hippocampus) + (cerebellum, muscle, granulocytes, pineal body, pancreas, kidney, liver, stomach, intestine,

									heart, spleen)
O. cuniculus	N/A	N/A	N/A	N/A	+ (hindbrain, heart)	+ (hindbrain, heart)	+ (hindbrain, forebrain, testes)	+ (hindbrain, forebrain, testes)	+ (hindbrain, forebrain, testes)
P. anubis	N/A	N/A	N/A	N/A	N/A	N/A	N/A	N/A	+ (brain)
R. norvegicus	N/A	N/A	N/A	N/A	N/A	N/A	N/A	N/A	++(thymus, cerebellum, lung, intestine, muscle, ovary, frontal cortex)<br>+ (esophagus, kidney, stomach, spleen, liver, testis, pancreas, heart, brain, adrenal gland)
S. scrofa	+	N/A	N/A	N/A	+ (cerebral cortex, ovary, forelimb bud,	+ (cerebral cortex, ovary, forelimb bud,	+ (cerebral cortex, ovary, forelimb bud,	++ (testis, omentum, ovary, brain)	++ (brain, kidney, lung)

					hindlimb bud)	hindlimb bud)	hindlimb bud)	+ (muscle, heart, pituitary gland, tonsil, lymph node, stomach, kidney, granulosa cell, kidney, epididymis, liver, spleen, penis, blood, intestine, uterus, adipose)	+ (blood, adipose, uterus)
X. laevis	+	+	+	+	+	+	+	N/A	L: + (brain, eyes, skin, spleen, ovary, liver, kidney, brain, testis, heart)<br>S: + (testis, kidney, skin, liver, ovary, spleen stomach, eye, heart, pancreas, lung)

X. tropicalis	-	-	+	+	+	+	+	N/A	++ (brain, testis)<br>+ (liver, ovary, heart, muscle, mesonephros)

Supplementary Table S7. Raw Expression Data of *Osgin* during Development. RGD – Rat Genome Database, [111,156]; MGI – Mouse Genome Informatics, [86,106,112, 115-157]; Developing Mouse Brain Atlas – [113]; Exp. Atlas – Expression Atlas, [97,107,109,115]; Xenbase – [84,108,114]; ISH – In situ hybridization; TPM – Transcripts Per Million; TPE – Transcripts Per Embryo; RPKM – Reads Per Kilobase Million; Low Expression Values – ≤ 10 TPM; Medium Expression Values – 11-1000 TPM; Note: range endpoints are rounded to encapsulate highest and lowest values. The paper following "Data from" provided raw data from which the proceeding database generated expression profiles/datasets. The databases list these papers as being sources for original data, so we have included them for complete transparency.

**Stage**	**Organism**	**Expression**	**Cell/Tissue Type**	**Method**	**Source**
Pre-fertilization	*X. laevis*	*Osgin1.l* (<10 TPM)<br>*Osgin2.l* (<10 TPM)<br>*Osgin2.s* (<10 TPM)	Oocyte	RNA-seq	Xenbase; Data from Session et al., 2016
Zygote/1 cell stage	*X. laevis*	*Osgin1.l* (0 TPM)<br>*Osgin2.l* (0.39 TPM)<br>*Osgin2.s* (0.15 TPM)	Zygote	RNA-seq	Xenbase; Data from Session et al., 2016
Zygote/1 cell stage	*X. tropicalis*	*Osgin1* (0 TPE)<br>*Osgin2* (6348 TPE)	Fertilized Oocyte	RNA-seq	Xenbase; Data from Owens et al., 2016

Blastula stage (stage 9)	*X. laevis*	*Osgin1.l* (0 TPM) *Osgin2.l* (2.16 TPM) *Osgin2.s* (0.39 TPM)	N/A	RNA-seq	Xenbase; Data from Session et al., 2016
Blastula stage (stage 8)	*X. tropicalis*	*Osgin1* (453 TPE) *Osgin2* (15752 TPE)	N/A	RNA-seq	Xenbase; Data from Owens et al., 2016
Gastrulation (Stage 12)	*X. laevis*	*Osgin1.l* (0 TPM) *Osgin2.l* (0.96 TPM) *Osgin2.s* (0.14 TPM)	N/A	RNA-seq	Xenbase; Data from Session et al., 2016
Gastrulation (Stage 10, 12)	*X. tropicalis*	*Osgin1* (606 TPE, 0 TPE) *Osgin2* (19217 TPE, 14685 TPE)	N/A	RNA-seq	Xenbase; Data from Owens et al., 2016
Hatching Stage (Stage 22, 39)	*X. tropicalis*	*Osgin1* (2860 TPE, 37657 TPE) *Osgin2* (449969 TPE, 1487980 TPE)	N/A	RNA-seq	Xenbase; Data from Owens et al., 2016
Organogenesis (from stage 10.5) to post natal period	*M. musculus*	*Osgin1*	Moderate signal in the liver from stage 11.5, in metanephros from TS26, ovary from TS27, testis at TS28	RNA-seq	Expression Atlas; Data from Cardoso-Moreira et al., 2019
Organogenesis (Stage 10.5)	*M. musculus*	*Osgin2*	Strong signal in the limb	RNA-seq	MGI; Data from Mirzamohammadi et al.,

					2018
Organogenesis (Stage 13.5)	*M. musculus*	*Osgin2*	Strong signal in the large intestine, ileum, and cecum	RNA-seq	MGI; Data from Delpretti et al., 2013
Organogenesis (stage 14.5)	*M. musculus*	*Osgin1* and *Osgin2*	Strong signal across the CNS	ISH	MGI; Data from Diez-Roux et al., 2011
Organogenesis (stage 14.5)	*M. musculus*	*Osgin1*	Strong signal in the liver	RNA-Seq	MGI; Data from Tallack et al., 2012
Organogenesis (stage 14.5)	*M. musculus*	*Osgin2*	Strong signal in the retina	RNA-seq	MGI; Data from Dupacova et al., 2021
Neurula stages (Stage 15, 20)	*X. laevis*	*Osgin1.l* (0.54 TPM, 7 TPM) *Osgin2.l* (1.32 TPM, 1.42 TPM) *Osgin2.s* (0.67 TPM, 1.23 TPM)	N/A	RNA-seq	Xenbase; Data from Session et al., 2016
Tailbud stage (stage 25, 29/30, 40)	*X. laevis*	*Osgin1.l* (5.83 TPM, 2.87 TPM, 0.86 TPM) *Osgin2.l* (1.33 TPM, 1.46 TPM, 1.27 TPM) *Osgin2.s* (1.72 TPM, 1.87 TPM, 1.62 TPM)	N/A	RNA-seq	Xenbase; Data from Session et al., 2016
Organogenesis (E12-E24)	*O. cuniculus*	*Osgin1*	Strongest signal in	RNA-seq	Exp. Atlas; Data from

		Osgin2	ovary and testis, Low to no signal in the CNS<br><br>Strongest signal in the hindbrain and heart		Cardoso-Moreira et al., 2019
Organogenesis (Carnegie Stage 13-Stage 23)	*H. sapiens*	*Osgin1*<br><br>*Osgin2* (<10-22 TPM)	Low to no signal in the CNS Comparatively higher values in spinal cord and cerebellum compared to other brain regions	RNA-seq	Exp. Atlas; Data from Lindsay et al., 2016
Fetal period	*H. sapiens*	*Osgin1* (7 TPM) *Osgin2* (13 TPM)	Signal in the proximal gut	RNA-seq	Expression Atlas; Data from Kraiczy et al., 2019
Fetal period (8 weeks - 20 weeks post conception)	*H. sapiens*	*Osgin1*<br><br>*Osgin2* (<10 - 32 TPM)	Low to no signal in the CNS Highest in cerebellum, spinal cord, and medulla oblongata	RNA-seq	Exp. Atlas; Data from Lindsay et al., 2016
Post-natal period	*M. musculus*	*Osgin1* and *Osgin2*	Strong signal in the mammary gland	RNA-seq	MGI; Data from Yamaji et al., 2013
Post-natal period	*M. musculus*	*Osgin1*	Signal in testis in RFX2 WT	RNA-seq	MGI; Data from Kistler et al., 2015

			but not KO mice		
Post-natal period	*M. musculus*	*Osgin1*	Strong signal in quadriceps femoris muscle, spleen, liver, right lung middle lobe, metanephros, testis, ascending colon but not visual cortex and heart left ventricle	RNA-seq	MGI; Data from Merkin et al., 2012
Post-natal period	*M. musculus*	*Osgin1*	Strong signal in adrenal medulla and low signal in carotid body (chemoreceptor that senses oxygen levels)	RNA-seq	MGI; Data from Chang et al., 2015
Post-natal period	*M. musculus*	*Osgin1*	Strong signal in Cnot complex deficient livers	RNA-seq	MGI; Data from Suzuki et al., 2019
Post-natal period	*M. musculus*	*Osgin1*	Strong signal in renal tubules of Aatf KO mice	RNA-seq	MGI; Data from Jain et al., 2019
Post-natal period	*M. musculus*	*Osgin1*	Strong signal in sciatic nerve of Miz1 POZ domain KO mice	RNA-seq	MGI; Data from Fuhrmann et al., 2018
Post-natal period	*M. musculus*	*Osgin1*	Moderate signal in	RNA-seq	MGI; Data from Bao et

			testis and low signal in spermatid and spermatocyte of WT and KO prospermatogonia-specific Upf2 mice		al., 2016
Post-natal period	*M. musculus*	*Osgin1*	Strong signal in Islet of Langerhans cells in Cnot3 KO	RNA-seq	MGI; Data from Mostafa et al., 2020
Post-natal period	*M. musculus*	*Osgin1*	Strong signal in colon in Gucy2c KO and WT	RNA-seq	MGI; Data from Mishra et al., 2021
Post-natal period	*M. musculus*	*Osgin1*	Strong signal in spermatids in H2A.B3 KO and WT	RNA-seq	MGI; Data from Anuar et al., 2019
Post-natal period	*M. musculus*	*Osgin1*	Strong signal in intestine smooth muscle and epithelium in MMP17 KO and WT cells	RNA-seq	MGI; Data from Martin-Alonso et al., 2021
Post-natal period	*M. musculus*	*Osgin1*	Moderate signal in white fat but low signal in brown fat	RNA-seq	MGI; Data from Takahashi et al., 2019
Post-natal period	*M. musculus*	*Osgin1*	Strong signal in islets of Langerhans	RNA-seq	MGI; Data from Kim et al., 2014
Post-natal period	*M. musculus*	*Osgin1* and *Osgin2*	Strong signal in the spleen	RNA-seq	MGI; Data from Patial et al., 2016

Post-natal period	*M. musculus*	*Osgin1*	Strong signal in olfactory epithelium	RNA-seq	MGI; Data from Haering et al., 2015
Post-natal period	*M. musculus*	*Osgin1*	Strong signal in olfactory epithelium	RNA-seq	MGI; Data from Haering et al., 2015
Post-natal period	*M. musculus*	*Osgin1*	Strong signal in abdomen musculature, gonadal fat pad, spleen, liver, lung, metanephros	RNA-seq	MGI; Data from West et al., 2016
Post-natal period	*M. musculus*	*Osgin1*	Strong signal in liver	RNA-seq	MGI; Data from Muller et al., 2018
Post-natal period	*M. musculus*	*Osgin1*	Moderate signal in prostate gland in Pten loss mice but low signal in WT mice	RNA-seq	MGI; Data from Guccini et al., 2021
Post-natal period	*M. musculus*	*Osgin1*	Strong signal in the liver	RNA-seq	MGI; Data from Munger et al., 2014
Post-natal period	*M. musculus*	*Osgin1* and *Osgin2*	Moderate signal in white fat tissue	RNA-seq	MGI; Data from Bond et al., 2021
Post-natal period	*M. musculus*	*Osgin1*	Strong signal in gastrocnemiu s muscle	RNA-seq	MGI; Data from Kim et al., 2014
Post-natal period	*M. musculus*	*Osgin2*	Strong signal in the spinal cord	RNA-seq	MGI; Data from O'Rourke et al., 2015

Post-natal period	*M. musculus*	*Osgin2*	Strong signal in the cerebellum	RNA-seq	MGI; Data from Ishimura et al., 2016
Post-natal period	*M. musculus*	*Osgin2*	Strong signal in the retina	RNA-seq	MGI; Data from Brooks et al., 2011
Post-natal period	*M. musculus*	*Osgin2*	Strong signal in the cerebral cortex	RNA-seq	MGI; Data from Narayanan et al., 2014
Post-natal period	*M. musculus*	*Osgin2*	Strong signal in the neural retina	RNA-seq	MGI; Data from Aldunate et al., 2019
Post-natal period	*M. musculus*	*Osgin2*	Strong signal in femur diaphysis of WT but not Dlx3Oc-cKO mice	RNA-seq	MGI; Data from Isaac et al., 2014
Post-natal period	*M. musculus*	*Osgin2*	Strong signal in the cochlea	RNA-seq	MGI; Data from Rousset et al., 2020
Post-natal period	*M. musculus*	*Osgin2*	Strong signal in the brain	RNA-seq	MGI; Data from Jena et al., 2020
Post-natal period	*M. musculus*	*Osgin2*	Strong signal in the brain, thymus, and testis	RNA-seq	MGI; Data from Huntley et al., 2016
Post-natal period	*M. musculus*	*Osgin2*	Strong signal in the corpus striatum	RNA-seq	MGI; Data from Zheng et al., 2015
Post-natal period	*M. musculus*	*Osgin2*	Strong signal in the cerebral cortex,	RNA-seq	MGI; Data from Kao et al., 2020

			cerebellum and spinal cord		
Post-natal period	*M. musculus*	*Osgin2*	Strong signal in the thalamus	RNA-seq	MGI; Data from Lipiec et al., 2020
Post-natal period	*M. musculus*	*Osgin2*	Strong signal in white and brown fat	RNA-seq	MGI; Data from Takahashi et al., 2019
Post-natal period	*M. musculus*	*Osgin2*	Strong signal in the brain	RNA-seq	MGI; Data from Klaus et al., 2020
Post-natal period	*M. musculus*	*Osgin2*	Strong signal in the hypothalamus	RNA-seq	MGI; Data from Alen et al., 2019
Post-natal period	*M. musculus*	*Osgin2*	Strong signal in the corpus callosum, brainstem, diencephalon, hippocampus, cerebellum, white fat, and skin	RNA-seq	MGI; Data from Langfelder et al., 2016
Post-natal period	*O. cuniculus*	*Osgin1*<br><br><br>*Osgin2*	Strongest signal in the liver, ovary, and kidney<br>Strongest signal in the forebrain, hindbrain, and testis	RNA-seq	Exp. Atlas; Data from Cardoso-Moreira et al., 2019
14 days (juvenile)	*R. norvegicus*	*Osgin1* (<10-82 TPM)	Medium in adrenal gland, brain, heart, kidney, lung, liver, gastrocnemiu	RNA-seq	RGD; Data from Yu et al., 2014

		Osgin2 (<10-30 TPM)	s, spleen, testes, and uterus; low in the thymus<br><br>Medium in adrenal gland, brain, heart, kidney, lung, liver, gastrocnemius, spleen, testes, uterus, and thymus		
1 month (adolescence)	*R. norvegicus*	*Osgin1* (<10-139 TPM)<br><br>*Osgin2* (<10-15 TPM)	Low in adrenal gland, brain, heart, lung, gastrocnemius, spleen, and uterus<br><br>Medium in kidney, liver, and testis<br><br>Low in adrenal gland, heart, kidney, liver, testis<br><br>Medium in brain, lung, gastrocnemius, spleen, thymus, and uterus	RNA-seq	RGD; Data from Yu et al., 2014

Supplementary Table S8. Raw *Osgin* expression data of vertebrates across several species. RGD – Rat Genome Database, [111,123,156,158,160]; Allen Mouse Brain – [113]; Exp. Atlas – Expression Atlas, [97,110,111,117,123,161-164]; Xenbase – [84,114]; NCBI – [41,165]; ProteomicsDB – [159]; ISH – In situ hybridization; Mass Spec. – Mass spectrometry; TPM – Transcripts Per Million; RPKM – Reads Per Kilobase Million; sFPKM – Significant Fragments

Per Kilobase Million; Note: specific values are provided for RNA-Seq data, RNA-Seq data range endpoints are rounded to include lowest and highest values). The paper following “Data from” provided raw data from which the proceeding database generated expression profiles/datasets. The databases list these papers as being sources for original data, so we have included them for complete transparency.

**Species**	**Tissue/ Organ/ Cell Type**	**Gene**	**Expression Notes/Values**	**Method**	**Source**
R. norvegicus, F344 strain	Visual cortex	*Osgin1*<br>*Osgin2*	<10 TPM<br>14 TPM	RNA-Seq	RGD; Data from Merkin et al., 2012
R. norvegicus, BN/SsNHsd strain	Visual cortex	*Osgin1*<br>*Osgin2*	<10 TPM<br>18 TPM	RNA-Seq	RGD; Data from Merkin et al., 2012
R. norvegicus, SD strain	Visual cortex	*Osgin1*<br>*Osgin2*	<10 TPM<br>12 TPM	RNA-Seq	RGD; Data from Merkin et al., 2012
R. norvegicus, F344 strain	Colon	*Osgin1*<br>*Osgin2*	<10 TPM<br><10 TPM	RNA-Seq	RGD; Data from Merkin et al., 2012
R. norvegicus, BN/SsNHsd strain	Colon	*Osgin1*<br>*Osgin2*	<10 TPM<br><10 TPM	RNA-Seq	RGD; Data from Merkin et al., 2012
R. norvegicus, SD strain	Colon	*Osgin1*<br>*Osgin2*	14 TPM<br><10 TPM	RNA-Seq	RGD; Data from Merkin et al., 2012
R. norvegicus, F344 strain	Heart Left Ventricle	*Osgin1*<br>*Osgin2*	<10 TPM<br><10 TPM	RNA-Seq	RGD; Data from Merkin et al., 2012

R. norvegicus, BN/SsNHsd strain	Heart Left Ventricle	*Osgin1*<br>*Osgin2*	<10 TPM<br><10 TPM	RNA-Seq	RGD; Data from Merkin et al., 2012
R. norvegicus, SD strain	Heart Left Ventricle	*Osgin1*<br>*Osgin2*	<10 TPM<br><10 TPM	RNA-Seq	RGD; Data from Merkin et al., 2012
R. norvegicus, F344 strain	Kidney	*Osgin1*<br>*Osgin2*	329 TPM<br><10 TPM	RNA-Seq	RGD; Data from Merkin et al., 2012
R. norvegicus, BN/SsNHsd strain	Kidney	*Osgin1*<br>*Osgin2*	284 TPM<br><10 TPM	RNA-Seq	RGD; Data from Merkin et al., 2012
R. norvegicus, SD strain	Kidney	*Osgin1*<br>*Osgin2*	492 TPM<br><10 TPM	RNA-Seq	RGD; Data from Merkin et al., 2012
R. norvegicus, F344 strain	Liver	*Osgin1*<br>*Osgin2*	138 TPM<br><10 TPM	RNA-Seq	RGD; Data from Merkin et al., 2012
R. norvegicus, BN/SsNHsd strain	Liver	*Osgin1*<br>*Osgin2*	136 TPM<br><10 TPM	RNA-Seq	RGD; Data from Merkin et al., 2012
R. norvegicus, SD strain	Liver	*Osgin1*<br>*Osgin2*	46 TPM<br><10 TPM	RNA-Seq	RGD; Data from Merkin et al., 2012
R. norvegicus, F344 strain	Lung	*Osgin1*<br>*Osgin2*	12 TPM<br>12 TPM	RNA-Seq	RGD; Data from Merkin et al., 2012

R. norvegicus, BN/SsNHsd strain	Lung	*Osgin1* *Osgin2*	11 TPM 17 TPM	RNA-Seq	RGD; Data from Merkin et al., 2012
R. norvegicus, SD strain	Lung	*Osgin1* *Osgin2*	16 TPM 15 TPM	RNA-Seq	RGD; Data from Merkin et al., 2012
R. norvegicus, F344 strain	skeletal muscle tissue of quadriceps femoris	*Osgin1* *Osgin2*	<10 TPM <10 TPM	RNA-Seq	RGD; Data from Merkin et al., 2012
R. norvegicus, BN/SsNHsd strain	skeletal muscle tissue of quadriceps femoris	*Osgin1* *Osgin2*	<10 TPM <10 TPM	RNA-Seq	RGD; Data from Merkin et al., 2012
R. norvegicus, SD strain	skeletal muscle tissue of quadriceps femoris	*Osgin1* *Osgin2*	<10 TPM <10 TPM	RNA-Seq	RGD; Data from Merkin et al., 2012
R. norvegicus, F344 strain	spleen	*Osgin1* *Osgin2*	<10 TPM 13 TPM	RNA-Seq	RGD; Data from Merkin et al., 2012
R. norvegicus, BN/SsNHsd strain	spleen	*Osgin1* *Osgin2*	<10 TPM <10 TPM	RNA-Seq	RGD; Data from Merkin et al., 2012
R. norvegicus, SD strain	spleen	*Osgin1* *Osgin2*	<10 TPM 11 TPM	RNA-Seq	RGD; Data from Merkin et al., 2012

R. norvegicus, F344 strain	testis	*Osgin1* *Osgin2*	149 TPM <10 TPM	RNA-Seq	RGD; Data from Merkin et al., 2012
R. norvegicus, BN/SsNHsd strain	testis	*Osgin1* *Osgin2*	152 TPM <10 TPM	RNA-Seq	RGD; Data from Merkin et al., 2012
R. norvegicus, SD strain	testis	*Osgin1* *Osgin2*	189 TPM <10 TPM	RNA-Seq	RGD; Data from Merkin et al., 2012
R. norvegicus	Adrenal gland	*Osgin1* *Osgin2*	29-128 TPM 11-25 TPM	RNA-Seq	RGD; Data from Naqvi et al., 2019
R. norvegicus	Brain	*Osgin1* *Osgin2*	<10 TPM 8-14 TPM	RNA-Seq	RGD; Data from Naqvi et al., 2019
R. norvegicus	Transverse colon	*Osgin1* *Osgin2*	5-15 TPM <10 TPM	RNA-Seq	RGD; Data from Naqvi et al., 2019
R. norvegicus	Heart	*Osgin1* *Osgin2*	<10 TPM <10 TPM	RNA-Seq	RGD; Data from Naqvi et al., 2019
R. norvegicus	Liver	*Osgin1* *Osgin2*	76-346 TPM <10 TPM	RNA-Seq	RGD; Data from Naqvi et al., 2019
R. norvegicus	Lung	*Osgin1* *Osgin2*	12-29 TPM 7-12 TPM	RNA-Seq	RGD; Data from Naqvi et al., 2019

R. norvegicus	Skeletal muscle tissue	*Osgin1*<br>*Osgin2*	11-16 TPM<br><10 TPM	RNA-Seq	RGD; Data from Naqvi et al., 2019
R. norvegicus	Zone of skin	*Osgin1*<br>*Osgin2*	8-17 TPM<br><10 TPM	RNA-Seq	RGD; Data from Naqvi et al., 2019
R. norvegicus	Spleen	*Osgin1*<br>*Osgin2*	<10 TPM<br><10 TPM	RNA-Seq	RGD; Data from Naqvi et al., 2019
R. norvegicus	Thyroid gland	*Osgin1*<br>*Osgin2*	28-62 TPM<br><10 TPM	RNA-Seq	RGD; Data from Naqvi et al., 2019
R. norvegicus	Pituitary gland	*Osgin1*<br>*Osgin2*	<10 TPM<br>7-11 TPM	RNA-Seq	RGD; Data from Naqvi et al., 2019
R. norvegicus	Adipose tissue	*Osgin1*<br>*Osgin2*	4-145 TPM<br>5-19 TPM	RNA-Seq	RGD; Data from Naqvi et al., 2019
R. norvegicus	Testis	*Osgin1*<br>*Osgin2*	83-94 TPM<br><10 TPM	RNA-Seq	RGD; Data from Naqvi et al., 2019
R. norvegicus 1-2 months, 1 year, 2 years	Cerebral cortex	*Osgin1*<br>*Osgin2*	Below baseline<br><10 TPM	RNA-Seq	RGD; Data from Darbellay et al., 2020
R. norvegicus 1-2 months	Kidney	*Osgin1*<br>*Osgin2*	254-767 TPM<br><10 TPM	RNA-Seq	RGD; Data from Darbellay et al., 2020

R. norvegicus 1 year	Kidney	*Osgin1* *Osgin2*	243-257 TPM <10 TPM	RNA-Seq	RGD; Data from Darbellay et al., 2020
R. norvegicus 1-2 months	Liver	*Osgin1* *Osgin2*	158-459 TPM <10 TPM	RNA-Seq	RGD; Data from Darbellay et al., 2020
R. norvegicus 1 year	Liver	*Osgin1* *Osgin2*	136-217 TPM <10 TPM	RNA-Seq	RGD; Data from Darbellay et al., 2020
R. norvegicus 2 years	Liver	*Osgin1* *Osgin2*	220-267 TPM <10 TPM	RNA-Seq	RGD; Data from Darbellay et al., 2020
R. norvegicus 1-2 months	Testis	*Osgin1* *Osgin2*	110-152 TPM <10 TPM	RNA-Seq	RGD; Data from Darbellay et al., 2020
R. norvegicus 2 years	Testis	*Osgin1* *Osgin2*	96-128 TPM <10 TPM	RNA-Seq	RGD; Data from Darbellay et al., 2020
H. sapiens	Nervous system	*Osgin1* *Osgin2*	2-6 TPM 9-11 TPM	RNA-Seq	Exp. Atlas; Data from GTEx Consortium
H. sapiens	Testis	*Osgin1* *Osgin2*	21 TPM 27 TPM	RNA-Seq	Exp. Atlas; Data from GTEx Consortium
H. sapiens	Prostate	*Osgin1* *Osgin2*	7 TPM 11 TPM	RNA-Seq	Exp. Atlas; Data from GTEx Consortium

H. sapiens	Ovary	*Osgin1*<br>*Osgin2*	5 TPM<br>20 TPM	RNA-Seq	Exp. Atlas; Data from GTEx Consortium
H. sapiens	Fallopian tube	*Osgin1*<br>*Osgin2*	10 TPM<br>13 TPM	RNA-Seq	Exp. Atlas; Data from GTEx Consortium
H. sapiens	Uterus	*Osgin1*<br>*Osgin2*	4 TPM<br>19 TPM	RNA-Seq	Exp. Atlas; Data from GTEx Consortium
H. sapiens	Vagina	*Osgin1*<br>*Osgin2*	6 TPM<br>9 TPM	RNA-Seq	Exp. Atlas; Data from GTEx Consortium
H. sapiens	Spleen	*Osgin1*<br>*Osgin2*	13 TPM<br>10 TPM	RNA-Seq	Exp. Atlas; Data from GTEx Consortium
H. sapiens	Digestive System	*Osgin1*<br>*Osgin2*	7-8 TPM<br>9-19 TPM	RNA-Seq	Exp. Atlas; Data from GTEx Consortium
H. sapiens	Adrenal gland	*Osgin1*<br>*Osgin2*	19 TPM<br>7 TPM	RNA-Seq	Exp. Atlas; Data from GTEx Consortium
H. sapiens	Regions of the heart	*Osgin1*<br>*Osgin2*	3 TPM<br>4 TPM	RNA-Seq	Exp. Atlas; Data from GTEx Consortium
H. sapiens	Aorta	*Osgin1*<br>*Osgin2*	12 TPM<br>20 TPM	RNA-Seq	Exp. Atlas; Data from GTEx Consortium

H. sapiens	Coronary artery	*Osgin1*<br>*Osgin2*	10 TPM<br>15 TPM	RNA-Seq	Exp. Atlas; Data from GTEx Consortium
H. sapiens	Tibial artery	*Osgin1*<br>*Osgin2*	11 TPM<br>20 TPM	RNA-Seq	Exp. Atlas; Data from GTEx Consortium
H. sapiens	Blood	*Osgin1*<br>*Osgin2*	3 TPM<br>14 TPM	RNA-Seq	Exp. Atlas; Data from GTEx Consortium
H. sapiens	Liver	*Osgin1*<br>*Osgin2*	44 TPM<br>6 TPM	RNA-Seq	Exp. Atlas; Data from GTEx Consortium
H. sapiens	Lung	*Osgin1*<br>*Osgin2*	11 TPM<br>21 TPM	RNA-Seq	Exp. Atlas; Data from GTEx Consortium
H. sapiens	Pancreas	*Osgin1*<br>*Osgin2*	4 TPM<br>3 TPM	RNA-Seq	Exp. Atlas; Data from GTEx Consortium
H. sapiens	Transformed skin fibroblast	*Osgin1*<br>*Osgin2*	4 TPM<br>15 TPM	RNA-Seq	Exp. Atlas; Data from GTEx Consortium
H. sapiens	Thyroid gland	*Osgin1*<br>*Osgin2*	10 TPM<br>14 TPM	RNA-Seq	Exp. Atlas; Data from GTEx Consortium
H. sapiens	Various tissues/organs from the nervous system	*Osgin1*<br>*Osgin2*	2-6 TPM<br>9-11 TPM	RNA-Seq	Exp. Atlas; Data from GTEx Consortium

H. sapiens	Adipose tissue	*Osgin1*<br>*Osgin2*	<10 TPM<br>47 TPM	RNA-Seq	Exp. Atlas; Data from Fagerberg et al., 2014
H. sapiens	Adrenal gland	*Osgin1*<br>*Osgin2*	11 TPM<br>19 TPM	RNA-Seq	Exp. Atlas; Data from Fagerberg et al., 2014
H. sapiens	Ovary	*Osgin1*<br>*Osgin2*	<10 TPM<br>44 TPM	RNA-Seq	Exp. Atlas; Data from Fagerberg et al., 2014
H. sapiens	Appendix	*Osgin1*<br>*Osgin2*	<10 TPM<br>24 TPM	RNA-Seq	Exp. Atlas; Data from Fagerberg et al., 2014
H. sapiens	Bladder	*Osgin1*<br>*Osgin2*	<10 TPM<br>29 TPM	RNA-Seq	Exp. Atlas; Data from Fagerberg et al., 2014
H. sapiens	Bone Marrow	*Osgin1*<br>*Osgin2*	<10 TPM<br>34 TPM	RNA-Seq	Exp. Atlas; Data from Fagerberg et al., 2014
H. sapiens	Cerebral Cortex	*Osgin1*<br>*Osgin2*	<10 TPM<br>48 TPM	RNA-Seq	Exp. Atlas; Data from Fagerberg et al., 2014
H. sapiens	Colon	*Osgin1*<br>*Osgin2*	<10 TPM<br>17 TPM	RNA-Seq	Exp. Atlas; Data from Fagerberg et al., 2014
H. sapiens	Duodenum	*Osgin1*<br>*Osgin2*	31 TPM<br>15 TPM	RNA-Seq	Exp. Atlas; Data from Fagerberg et al., 2014

H. sapiens	Endometrium	*Osgin1*<br>*Osgin2*	<10 TPM<br>65 TPM	RNA-Seq	Exp. Atlas; Data from Fagerberg et al., 2014
H. sapiens	Esophagus	*Osgin1*<br>*Osgin2*	<10 TPM<br>18 TPM	RNA-Seq	Exp. Atlas; Data from Fagerberg et al., 2014
H. sapiens	Gallbladder	*Osgin1*<br>*Osgin2*	12 TPM<br>24 TPM	RNA-Seq	Exp. Atlas; Data from Fagerberg et al., 2014
H. sapiens	Heart	*Osgin1*<br>*Osgin2*	<10 TPM<br>12 TPM	RNA-Seq	Exp. Atlas; Data from Fagerberg et al., 2014
H. sapiens	Kidney	*Osgin1*<br>*Osgin2*	<10 TPM<br>21 TPM	RNA-Seq	Exp. Atlas; Data from Fagerberg et al., 2014
H. sapiens	Liver	*Osgin1*<br>*Osgin2*	44 TPM<br>16 TPM	RNA-Seq	Exp. Atlas; Data from Fagerberg et al., 2014
H. sapiens	Lung	*Osgin1*<br>*Osgin2*	<10 TPM<br>43 TPM	RNA-Seq	Exp. Atlas; Data from Fagerberg et al., 2014
H. sapiens	Lymph Node	*Osgin1*<br>*Osgin2*	<10 TPM<br>26 TPM	RNA-Seq	Exp. Atlas; Data from Fagerberg et al., 2014
H. sapiens	Pancreas	*Osgin1*<br>*Osgin2*	<10 TPM<br><10 TPM	RNA-Seq	Exp. Atlas; Data from Fagerberg et al., 2014

H. sapiens	Placenta	*Osgin1*<br>*Osgin2*	<10 TPM<br>22 TPM	RNA-Seq	Exp. Atlas; Data from Fagerberg et al., 2014
H. sapiens	Prostate	*Osgin1*<br>*Osgin2*	<10 TPM<br>27 TPM	RNA-Seq	Exp. Atlas; Data from Fagerberg et al., 2014
H. sapiens	Salivary Gland	*Osgin1*<br>*Osgin2*	<10 TPM<br>10 TPM	RNA-Seq	Exp. Atlas; Data from Fagerberg et al., 2014
H. sapiens	Skin	*Osgin1*<br>*Osgin2*	<10 TPM<br>10 TPM	RNA-Seq	Exp. Atlas; Data from Fagerberg et al., 2014
H. sapiens	Small intestine	*Osgin1*<br>*Osgin2*	21 TPM<br>14 TPM	RNA-Seq	Exp. Atlas; Data from Fagerberg et al., 2014
H. sapiens	Spleen	*Osgin1*<br>*Osgin2*	<10 TPM<br>23 TPM	RNA-Seq	Exp. Atlas; Data from Fagerberg et al., 2014
H. sapiens	Stomach	*Osgin1*<br>*Osgin2*	<10 TPM<br>11 TPM	RNA-Seq	Exp. Atlas; Data from Fagerberg et al., 2014
H. sapiens	Testis	*Osgin1*<br>*Osgin2*	12 TPM<br>52 TPM	RNA-Seq	Exp. Atlas; Data from Fagerberg et al., 2014
H. sapiens	Thyroid	*Osgin1*<br>*Osgin2*	<10 TPM<br>48 TPM	RNA-Seq	Exp. Atlas; Data from Fagerberg et al., 2014

B. taurus	Brain	*Osgin1*<br>*Osgin2*	9 TPM<br>58 TPM	RNA-Seq	Exp. Atlas; Data from Merkin et al., 2012
B. taurus	Colon	*Osgin1*<br>*Osgin2*	<10 TPM<br>14 TPM	RNA-Seq	Exp. Atlas; Data from Merkin et al., 2012
B. taurus	Heart	*Osgin1*<br>*Osgin2*	<10 TPM<br>13 TPM	RNA-Seq	Exp. Atlas; Data from Merkin et al., 2012
B. taurus	Kidney	*Osgin1*<br>*Osgin2*	55 TPM<br>27 TPM	RNA-Seq	Exp. Atlas; Data from Merkin et al., 2012
B. taurus	Liver	*Osgin1*<br>*Osgin2*	86 TPM<br>35 TPM	RNA-Seq	Exp. Atlas; Data from Merkin et al., 2012
B. taurus	Lung	*Osgin1*<br>*Osgin2*	12 TPM<br>33 TPM	RNA-Seq	Exp. Atlas; Data from Merkin et al., 2012
B. taurus	Skeletal Muscle Tissue	*Osgin1*<br>*Osgin2*	<10 TPM<br>22 TPM	RNA-Seq	Exp. Atlas; Data from Merkin et al., 2012
B. taurus	Spleen	*Osgin1*<br>*Osgin2*	<10 TPM<br>56 TPM	RNA-Seq	Exp. Atlas; Data from Merkin et al., 2012
B. taurus	Testis	*Osgin1*<br>*Osgin2*	73 TPM<br>24 TPM	RNA-Seq	Exp. Atlas; Data from Merkin et al., 2012

M. mulatta	Brain	*Osgin1*<br>*Osgin2*	15 TPM<br>59 TPM	RNA-Seq	Exp. Atlas; Data from Merkin et al., 2012
M. mulatta	Colon	*Osgin1*<br>*Osgin2*	17 TPM<br>58 TPM	RNA-Seq	Exp. Atlas; Data from Merkin et al., 2012
M. mulatta	Heart	*Osgin1*<br>*Osgin2*	53 TPM<br>64 TPM	RNA-Seq	Exp. Atlas; Data from Merkin et al., 2012
M. mulatta	Kidney	*Osgin1*<br>*Osgin2*	131 TPM<br>59 TPM	RNA-Seq	Exp. Atlas; Data from Merkin et al., 2012
M. mulatta	Liver	*Osgin1*<br>*Osgin2*	65 TPM<br>23 TPM	RNA-Seq	Exp. Atlas; Data from Merkin et al., 2012
M. mulatta	Lung	*Osgin1*<br>*Osgin2*	15 TPM<br>99 TPM	RNA-Seq	Exp. Atlas; Data from Merkin et al., 2012
M. mulatta	Skeletal Muscle Tissue	*Osgin1*<br>*Osgin2*	18 TPM<br>52 TPM	RNA-Seq	Exp. Atlas; Data from Merkin et al., 2012
M. mulatta	Spleen	*Osgin1*<br>*Osgin2*	14 TPM<br>53 TPM	RNA-Seq	Exp. Atlas; Data from Merkin et al., 2012
M. mulatta	Testis	*Osgin1*<br>*Osgin2*	22 TPM<br>65 TPM	RNA-Seq	Exp. Atlas; Data from Merkin et al., 2012

M. musculus	Brain	*Osgin1*<br>*Osgin2*	<10 TPM<br>18 TPM	RNA-Seq	Exp. Atlas; Data from Huntley et al., 2016
M. musculus	Heart	*Osgin1*<br>*Osgin2*	<10 TPM<br><10 TPM	RNA-Seq	Exp. Atlas; Data from Huntley et al., 2016
M. musculus	Kidney	*Osgin1*<br>*Osgin2*	59 TPM<br><10 TPM	RNA-Seq	Exp. Atlas; Data from Huntley et al., 2016
M. musculus	Liver	*Osgin1*<br>*Osgin2*	313 TPM<br><10 TPM	RNA-Seq	Exp. Atlas; Data from Huntley et al., 2016
M. musculus	Lung	*Osgin1*<br>*Osgin2*	28 TPM<br>10 TPM	RNA-Seq	Exp. Atlas; Data from Huntley et al., 2016
M. musculus	Spleen	*Osgin1*<br>*Osgin2*	8 TPM<br>10 TPM	RNA-Seq	Exp. Atlas; Data from Huntley et al., 2016
M. musculus	Testis	*Osgin1*<br>*Osgin2*	272 TPM<br>11 TPM	RNA-Seq	Exp. Atlas; Data from Huntley et al., 2016
M. musculus	Thymus	*Osgin1*<br>*Osgin2*	3 TPM<br>11 TPM	RNA-Seq	Exp. Atlas; Data from Huntley et al., 2016
M. musculus	Zone of skin	*Osgin1*<br>*Osgin2*	20 TPM<br>9 TPM	RNA-Seq	Exp. Atlas; Data from Huntley et al., 2016

M. musculus	Brain	*Osgin1*<br>*Osgin2*	<10 TPM<br>17 TPM	RNA-Seq	Exp. Atlas; Data from Merkin et al., 2012
M. musculus	Colon	*Osgin1*<br>*Osgin2*	44 TPM<br><10 TPM	RNA-Seq	Exp. Atlas; Data from Merkin et al., 2012
M. musculus	Heart	*Osgin1*<br>*Osgin2*	<10 TPM<br><10 TPM	RNA-Seq	Exp. Atlas; Data from Merkin et al., 2012
M. musculus	Kidney	*Osgin1*<br>*Osgin2*	60 TPM<br><10 TPM	RNA-Seq	Exp. Atlas; Data from Merkin et al., 2012
M. musculus	Liver	*Osgin1*<br>*Osgin2*	250 TPM<br><10 TPM	RNA-Seq	Exp. Atlas; Data from Merkin et al., 2012
M. musculus	Lung	*Osgin1*<br>*Osgin2*	68 TPM<br>11 TPM	RNA-Seq	Exp. Atlas; Data from Merkin et al., 2012
M. musculus	Skeletal Muscle Tissue	*Osgin1*<br>*Osgin2*	19 TPM<br><10 TPM	RNA-Seq	Exp. Atlas; Data from Merkin et al., 2012
M. musculus	Spleen	*Osgin1*<br>*Osgin2*	13 TPM<br>12 TPM	RNA-Seq	Exp. Atlas; Data from Merkin et al., 2012
M. musculus	Testis	*Osgin1*<br>*Osgin2*	228 TPM<br>10 TPM	RNA-Seq	Exp. Atlas; Data from Merkin et al., 2012

M. musculus	Brain	*Osgin1*<br>*Osgin2*	<10 TPM<br><10 TPM	RNA-Seq	Exp. Atlas; Data from Barbosa-Morais et al., 2012
M. musculus	Heart	*Osgin1*<br>*Osgin2*	<10 TPM<br><10 TPM	RNA-Seq	Exp. Atlas; Data from Barbosa-Morais et al., 2012
M. musculus	Kidney	*Osgin1*<br>*Osgin2*	73 TPM<br><10 TPM	RNA-Seq	Exp. Atlas; Data from Barbosa-Morais et al., 2012
M. musculus	Liver	*Osgin1*<br>*Osgin2*	191 TPM<br><10 TPM	RNA-Seq	Exp. Atlas; Data from Barbosa-Morais et al., 2012
M. musculus	Skeletal Muscle Tissue	*Osgin1*<br>*Osgin2*	22 TPM<br><10 TPM	RNA-Seq	Exp. Atlas; Data from Barbosa-Morais et al., 2012
M. musculus	Brain	*Osgin1*<br>*Osgin2*	<10 TPM<br>12 TPM	RNA-Seq	Exp. Atlas; Data from Brawand et al., 2011
M. musculus	Cerebellum	*Osgin1*<br>*Osgin2*	<10 TPM<br>12 TPM	RNA-Seq	Exp. Atlas; Data from Brawand et al., 2011
M. musculus	Heart	*Osgin1*<br>*Osgin2*	<10 TPM<br><10 TPM	RNA-Seq	Exp. Atlas; Data from Brawand et al., 2011

M. musculus	Kidney	*Osgin1*<br>*Osgin2*	69 TPM<br><10 TPM	RNA-Seq	Exp. Atlas; Data from Brawand et al., 2011
M. musculus	Liver	*Osgin1*<br>*Osgin2*	95 TPM<br><10 TPM	RNA-Seq	Exp. Atlas; Data from Brawand et al., 2011
M. musculus	Testis	*Osgin1*<br>*Osgin2*	231 TPM<br><10 TPM	RNA-Seq	Exp. Atlas; Data from Brawand et al., 2011
M. musculus	Caecum	*Osgin1*<br>*Osgin2*	<10 TPM<br>14 TPM	RNA-Seq	Exp. Atlas; Data from Delpretti et al., 2013
M. musculus	Colon	*Osgin1*<br>*Osgin2*	<10 TPM<br>13 TPM	RNA-Seq	Exp. Atlas; Data from Delpretti et al., 2013
M. musculus	Ileum	*Osgin1*<br>*Osgin2*	<10 TPM<br>14 TPM	RNA-Seq	Exp. Atlas; Data from Delpretti et al., 2013
M. musculus	Brain	*Osgin1*<br>*Osgin2*	N/A<br>10 TPM	RNA-Seq	Exp. Atlas; Data from Soumillon et al., 2013
M. musculus	Liver	*Osgin1*<br>*Osgin2*	31 TPM<br><10 TPM	RNA-Seq	Exp. Atlas; Data from Soumillon et al., 2013
M. musculus	Testis	*Osgin1*<br>*Osgin2*	58 TPM<br><10 TPM	RNA-Seq	Exp. Atlas; Data from Soumillon et al., 2013

P. anubis	Bone marrow	*Osgin1*<br>*Osgin2*	<10 TPM<br><10 TPM	RNA-Seq	Exp. Atlas; Data from Pipes et al., 2013
P. anubis	Cerebellum	*Osgin1*<br>*Osgin2*	<10 TPM<br>25 TPM	RNA-Seq	Exp. Atlas; Data from Pipes et al., 2013
P. anubis	Colon	*Osgin1*<br>*Osgin2*	<10 TPM<br>14 TPM	RNA-Seq	Exp. Atlas; Data from Pipes et al., 2013
P. anubis	Frontal Cortex	*Osgin1*<br>*Osgin2*	<10 TPM<br>26 TPM	RNA-Seq	Exp. Atlas; Data from Pipes et al., 2013
P. anubis	Heart	*Osgin1*<br>*Osgin2*	<10 TPM<br>11 TPM	RNA-Seq	Exp. Atlas; Data from Pipes et al., 2013
P. anubis	Kidney	*Osgin1*<br>*Osgin2*	56 TPM<br>17 TPM	RNA-Seq	Exp. Atlas; Data from Pipes et al., 2013
P. anubis	Liver	*Osgin1*<br>*Osgin2*	49 TPM<br>11 TPM	RNA-Seq	Exp. Atlas; Data from Pipes et al., 2013
P. anubis	Lung	*Osgin1*<br>*Osgin2*	<10 TPM<br>34 TPM	RNA-Seq	Exp. Atlas; Data from Pipes et al., 2013
P. anubis	Lymph Node	*Osgin1*<br>*Osgin2*	<10 TPM<br>18 TPM	RNA-Seq	Exp. Atlas; Data from Pipes et al., 2013

P. anubis	Pituitary Gland	*Osgin1*<br>*Osgin2*	<10 TPM<br>34 TPM	RNA-Seq	Exp. Atlas; Data from Pipes et al., 2013
P. anubis	Skeletal Muscle Tissue	*Osgin1*<br>*Osgin2*	11 TPM<br>27 TPM	RNA-Seq	Exp. Atlas; Data from Pipes et al., 2013
P. anubis	Spleen	*Osgin1*<br>*Osgin2*	<10 TPM<br>21 TPM	RNA-Seq	Exp. Atlas; Data from Pipes et al., 2013
P. anubis	Temporal Lobe	*Osgin1*<br>*Osgin2*	<10 TPM<br>28 TPM	RNA-Seq	Exp. Atlas; Data from Pipes et al., 2013
M. musculus	Midbrain	*Osgin1*<br><br>*Osgin2*	Strongest in the olfactory cortex<br><br>Strongest in the hippocampus and isocortex	ISH	Allen Developing Mouse Brain Atlas
H. sapiens	Liver	*Osgin1*<br>*Osgin2*	11 RPKM<br><1 RPKM	RNA-Seq	NCBI; Data from Duff et al., 2015
H. sapiens	Kidney	*Osgin1*<br>*Osgin2*	1.7 RPKM<br>1.4 RPKM	RNA-Seq	NCBI; Data from Duff et al., 2015
H. sapiens	Skeletal muscle	*Osgin1*<br>*Osgin2*	1.6 RPKM<br><1 RPKM	RNA-Seq	NCBI; Data from Duff et al., 2015
H. sapiens	Cerebellum	*Osgin1*<br>*Osgin2*	<1 RPKM<br>2 RPKM	RNA-Seq	NCBI; Data from Duff et al., 2015

H. sapiens	Adrenal gland	*Osgin1*<br>*Osgin2*	1.5 RPKM<br><1 RPKM	RNA-Seq	NCBI; Data from Duff et al., 2015
H. sapiens	Lung, small intestine, duodenum	*Osgin1* (Q9UJX0)<br>*Osgin1* (Q9UJX0-2)	Highest expression in lung	Mass spectrometry	Soumillon et al., 2013
H. sapiens	Skin, lung	*Osgin1* (H3BTF9)	Highest expression in skin	Mass spectrometry	Soumillon et al., 2013
H. sapiens	Circulatory system	*Osgin2* (Q9Y236)	Highest expression in circulatory system	Mass spectrometry	Soumillon et al., 2013
H. sapiens	Circulatory system	*Osgin2* (Q9Y236)<br><br>*Osgin2*(Q9Y236-2)	Highest expression in circulatory system	Mass spectrometry	Soumillon et al., 2013
X. laevis	Brain	*Osgin1.L*<br>*Osgin2.L*<br>*Osgin2.S*	1.24 TPM<br>6.86 TPM<br>9.04 TPM	RNA-Seq	Xenbase; Data from Session et al., 2016
X. laevis	Eyes	*Osgin1.L*<br>*Osgin2.L*<br>*Osgin2.S*	2.35 TPM<br>10.77 TPM<br>10.96 TPM	RNA-Seq	Xenbase; Data from Session et al., 2016
X. laevis	Heart	*Osgin1.L*<br>*Osgin2.L*<br>*Osgin2.S*	0.79 TPM<br>1.55 TPM<br>2.88 TPM	RNA-Seq	Xenbase; Data from Session et al., 2016
X. laevis	Intestine	*Osgin1.L*<br>*Osgin2.L*<br>*Osgin2.S*	43.71 TPM<br>0.83 TPM<br>0.82 TPM	RNA-Seq	Xenbase; Data from Session et al., 2016

X. laevis	Kidney	*Osgin1.L*<br>*Osgin2.L*<br>*Osgin2.S*	8.1 TPM<br>5.73 TPM<br>10.15 TPM	RNA-Seq	Xenbase; Data from Session et al., 2016
X. laevis	Liver	*Osgin1.L*<br>*Osgin2.L*<br>*Osgin2.S*	4.74 TPM<br>1.14 TPM<br>1.71 TPM	RNA-Seq	Xenbase; Data from Session et al., 2016
X. laevis	Lung	*Osgin1.L*<br>*Osgin2.L*<br>*Osgin2.S*	1.37 TPM<br>0.92 TPM<br>2.77 TPM	RNA-Seq	Xenbase; Data from Session et al., 2016
X. laevis	Muscle	*Osgin1.L*<br>*Osgin2.L*<br>*Osgin2.S*	2.26 TPM<br>0.74 TPM<br>0.16 TPM	RNA-Seq	Xenbase; Data from Session et al., 2016
X. laevis	Ovary	*Osgin1.L*<br>*Osgin2.L*<br>*Osgin2.S*	0.02 TPM<br>1.44 TPM<br>0.82 TPM	RNA-Seq	Xenbase; Data from Session et al., 2016
X. laevis	Pancreas	*Osgin1.L*<br>*Osgin2.L*<br>*Osgin2.S*	0.68 TPM<br>0.13 TPM<br>0.51 TPM	RNA-Seq	Xenbase; Data from Session et al., 2016
X. laevis	Skin	*Osgin1.L*<br>*Osgin2.L*<br>*Osgin2.S*	0.21 TPM<br>8.93 TPM<br>4.16 TPM	RNA-Seq	Xenbase; Data from Session et al., 2016
X. laevis	Stomach	*Osgin1.L*<br>*Osgin2.L*<br>*Osgin2.S*	6.15 TPM<br>0.67 TPM<br>1.7 TPM	RNA-Seq	Xenbase; Data from Session et al., 2016
X. laevis	Spleen	*Osgin1.L*<br>*Osgin2.L*<br>*Osgin2.S*	1.06 TPM<br>5 TPM<br>4.11 TPM	RNA-Seq	Xenbase; Data from Session et al., 2016

X. laevis	Testis	*Osgin1.L*<br>*Osgin2.L*<br>*Osgin2.S*	2.01 TPM<br>2.68 TPM<br>3.78 TPM	RNA-Seq	Xenbase; Data from Session et al., 2016
R. norvegicus, 4 months	Various organs	*Osgin1* (<10-99 TPM)<br><br>*Osgin2* (<10-13 TPM)	Low in brain, heart, lung, gastrocnemius, spleen, and uterus<br><br>Medium in adrenal gland, kidney, liver, and testis<br><br>Low in heart, kidney, liver, and testis<br><br>Medium in adrenal gland, brain, lung, gastrocnemius, spleen, thymus, and uterus	RNA-seq	RGD; Data from Yu et al., 2014
R. norvegicus, 1 year	Various organs	*Osgin1* (<10-90 TPM)<br><br>*Osgin2* (<10-15 TPM)	Low in brain, heart, lung, gastrocnemius, spleen, thymus, and uterus<br><br>Medium in adrenal gland, kidney, liver, and testis<br><br>Low in heart, kidney, liver, thymus, testis, and uterus<br><br>Medium in adrenal gland, brain, lung, gastrocnemius, and spleen	RNA-seq	RGD; Data from Yu et al., 2014

Supplementary Table S9. Differential *Osgin* expression comparing pathological to nonpathological cells and tissues (unless otherwise specified) in cancers and precancerous

conditions. Exp. Atlas – Expression Atlas, [97-101,166-173]; The Pan-Cancer Analysis of Whole Genomes Project – [98]. The paper following "Data from" provided raw data from which the proceeding database generated expression profiles/datasets. The databases list these papers as being sources for original data, so we have included them for complete transparency.

**Pathology**	**Cell/Tissue Type**	***Osgin* Expression**	**Method**	**Source**
Astrocytoma	Human brain tumor samples	*Osgin2* upregulation	Microarray	Exp. Atlas; Madhavan et al., 2009
Atypical teratoid/ rhabdoid tumors	Human atypical teratoid/rhabdoid tumors	*Osgin1* downregulation	Microarray	Exp. Atlas; Data from Birks et al., 2013
B-cell non-Hodgkin lymphoma	Human blood cells	*Osgin1* downregulation	RNA-Seq	Exp. Atlas; Data from Pan-Cancer Analysis of Whole Genomes Project
B-cell non-Hodgkin lymphoma	Human blood cells	*Osgin2* upregulation	RNA-Seq	Exp. Atlas; Data from Pan-Cancer Analysis of Whole Genomes Project
Bladder transitional cell carcinoma	Human urinary bladder cells	*Osgin1* downregulation	RNA-Seq	Exp. Atlas; Data from Pan-Cancer Analysis of Whole Genomes Project
Breast adenocarcinoma	Human breast tumor samples	*Osgin1* downregulation	RNA-Seq	Exp. Atlas; Data from Pan-Cancer Analysis of Whole Genomes Project
Breast adenocarcinoma	Human breast tumor samples	*Osgin2* upregulation	RNA-Seq	Exp. Atlas; Data from Pan-Cancer Analysis of Whole Genomes Project

Breast Cancer	Human non-triple-negative breast cancer tissue	*Osgin1* downregulation	RNA-Seq	Exp. Atlas
Breast Cancer	Human non-triple-negative breast cancer tissue	*Osgin2* upregulation	RNA-Seq	Exp. Atlas
Breast Cancer	Human triple-negative breast cancer tissue	*Osgin1* downregulation	RNA-Seq	Exp. Atlas
Breast Cancer	Human triple-negative breast cancer tissue	*Osgin2* upregulation	RNA-Seq	Exp. Atlas
Breast cancer	Human HER2-positive breast cancer tissue	*Osgin1* downregulation	RNA-Seq	Exp. Atlas
Breast cancer	Human HER2-positive breast cancer tissue	*Osgin2* upregulation	RNA-Seq	Exp. Atlas
Cervical Adenocarcinoma	Human uterine cervix samples	*Osgin1* downregulation	RNA-Seq	Exp. Atlas; Data from Pan-Cancer Analysis of Whole Genomes Project
Cervical Adenocarcinoma	Human uterine cervix samples	*Osgin2* upregulation	RNA-Seq	Exp. Atlas; Data from Pan-Cancer Analysis of Whole Genomes Project
Cervical Squamous Cell Carcinoma	Human uterine cervix samples	*Osgin1* downregulation	RNA-Seq	Exp. Atlas; Data from Pan-Cancer Analysis of Whole Genomes Project
Cholangiocarcinoma	Human liver cells	*Osgin1* downregulation	RNA-Seq	Exp. Atlas; Data from Pan-

				Cancer Analysis of Whole Genomes Project
Cholangiocarcinoma	Human liver cells	*Osgin2* upregulation	RNA-Seq	Exp. Atlas; Data from Pan-Cancer Analysis of Whole Genomes Project
Chromophobe renal cell carcinoma	Human kidney cells	*Osgin2* upregulation	RNA-Seq	Exp. Atlas; Data from Pan-Cancer Analysis of Whole Genomes Project
Chronic lymphocytic leukemia	Human blood cells	*Osgin1* downregulation	RNA-Seq	Exp. Atlas; Data from Pan-Cancer Analysis of Whole Genomes Project
Chronic lymphocytic leukemia	Human blood cells	*Osgin2* upregulation	RNA-Seq	Exp. Atlas; Data from Pan-Cancer Analysis of Whole Genomes Project
Clear Cell Sarcoma	*Mus musculus* clear cell sarcoma model tumor induced by TAT-cre	*Osgin1* downregulation	RNA-Seq	Exp. Atlas; Data from Straessler et al., 2013
Clear Cell Sarcoma	*Mus musculus* clear cell carcinoma model tumor induced by *Rosa26CreER*	*Osgin1* downregulation	RNA-Seq	Exp. Atlas; Data from Straessler et al., 2013
Colon Adenocarcinoma	Human colon samples	*Osgin1* downregulation	RNA-Seq	Exp. Atlas; Data from Kanth et al., 2016
Colon Hyperplastic Polyps	Human sessile serrated colon adenoma/polyps	*Osgin1* upregulation	RNA-Seq	Exp. Atlas; Data from Kanth et al., 2016

Colorectal Adenocarcinoma	Human large intestine tissue samples	*Osgin1* downregulation	RNA-Seq	Exp. Atlas; Data from Pan-Cancer Analysis of Whole Genomes Project
Colorectal Adenocarcinoma	Human large intestine tissue samples	*Osgin2* downregulation	RNA-Seq	Exp. Atlas; Data from Pan-Cancer Analysis of Whole Genomes Project
Dysplasia	*Rattus norvegicus* tongue epithelial cells	*Osgin1* upregulation	Microarray	Exp. Atlas; Data from Wu et al., 2016
Endometrial adenocarcinoma	Human uterine tissue samples	*Osgin2* downregulation	RNA-Seq	Exp. Atlas; Data from Pan-Cancer Analysis of Whole Genomes Project
Ependymoma	Adult human brain tumors	*Osgin2* upregulation	Microarray	Exp. Atlas; Data from Griesinger et al., 2015
Erythroleukemia	Human bone marrow samples	*Osgin1* upregulation	RNA-Seq	Exp. Atlas; Data from Madan et al., 2015
Esophageal Adenocarcinoma	Human esophageal tumor samples	*Osgin1* upregulation	RNA-Seq	Exp. Atlas; Data from Pan-Cancer Analysis of Whole Genomes Project
Esophageal Adenocarcinoma	Human esophageal junction tumor samples	*Osgin2* downregulation	RNA-Seq	Exp. Atlas; Data from Pan-Cancer Analysis of Whole Genomes Project
Esophageal Adenocarcinoma	Human esophageal mucosal cells	*Osgin2* upregulation	RNA-Seq	Exp. Atlas; Data from Pan-Cancer Analysis of Whole

				Genomes Project
Esophageal Adenocarcinoma	Human esophageal muscularis mucosal cells	*Osgin2* downregulation	RNA-Seq	Exp. Atlas; Data from Pan-Cancer Analysis of Whole Genomes Project
Follicular Thyroid carcinoma	Human thyroid gland cells	*Osgin1* downregulation	RNA-Seq	Exp. Atlas; Data from Pan-Cancer Analysis of Whole Genomes Project
Follicular Thyroid carcinoma	Human thyroid gland cells	*Osgin2* downregulation	RNA-Seq	Exp. Atlas; Data from Pan-Cancer Analysis of Whole Genomes Project
Gastric Adenocarcinoma	Human stomach cells	*Osgin1* downregulation	RNA-Seq	Exp. Atlas; Data from Pan-Cancer Analysis of Whole Genomes Project
Gastric Adenocarcinoma	Human stomach cells	*Osgin2* upregulation	RNA-Seq	Exp. Atlas; Data from Pan-Cancer Analysis of Whole Genomes Project
Glioblastoma multiforme	Human glioblastoma multiforme tumors - amygdala	*Osgin1* upregulation	RNA-Seq	Exp. Atlas; Data from Pan-Cancer Analysis of Whole Genomes Project
Glioblastoma multiforme	Human glioblastoma multiforme tumors - caudate nucleus	*Osgin2* upregulation	RNA-Seq	Exp. Atlas; Data from Pan-Cancer Analysis of Whole Genomes Project
Glioblastoma multiforme	Human glioblastoma multiforme	*Osgin1* upregulation	RNA-Seq	Exp. Atlas; Data from Pan-Cancer Analysis

	tumors - cerebellar hemisphere			of Whole Genomes Project
Glioblastoma multiforme	Human glioblastoma multiforme tumors - cerebellar hemisphere	*Osgin2* upregulation	RNA-Seq	Exp. Atlas; Data from Pan-Cancer Analysis of Whole Genomes Project
Glioblastoma multiforme	Human glioblastoma multiforme tumors - cerebellum	*Osgin1* upregulation	RNA-Seq	Exp. Atlas; Data from Pan-Cancer Analysis of Whole Genomes Project
Glioblastoma multiforme	Human glioblastoma multiforme tumors - cerebellum	*Osgin2* upregulation	RNA-Seq	Exp. Atlas; Data from Pan-Cancer Analysis of Whole Genomes Project
Glioblastoma multiforme	Human glioblastoma multiforme tumors - cerebral cortex	*Osgin2* upregulation	RNA-Seq	Exp. Atlas; Data from Pan-Cancer Analysis of Whole Genomes Project
Glioblastoma multiforme	Human glioblastoma multiforme tumors - hippocampus proper	*Osgin1* upregulation	RNA-Seq	Exp. Atlas; Data from Pan-Cancer Analysis of Whole Genomes Project
Glioblastoma multiforme	Human glioblastoma multiforme tumors - hippocampus proper	*Osgin2* upregulation	RNA-Seq	Exp. Atlas; Data from Pan-Cancer Analysis of Whole Genomes Project
Glioblastoma multiforme	Human glioblastoma multiforme tumors -	*Osgin2* upregulation	RNA-Seq	Exp. Atlas; Data from Pan-Cancer Analysis of Whole

	hypothalamus			Genomes Project
Glioblastoma multiforme	Human glioblastoma multiforme tumors - nucleus accumbens	*Osgin1* downregulation	RNA-Seq	Exp. Atlas; Data from Pan-Cancer Analysis of Whole Genomes Project
Glioblastoma multiforme	Human glioblastoma multiforme tumors - amygdala	*Osgin2* upregulation	RNA-Seq	Exp. Atlas; Data from Pan-Cancer Analysis of Whole Genomes Project
Glioblastoma multiforme	Human glioblastoma multiforme tumors - nucleus accumbens	*Osgin2* upregulation	RNA-Seq	Exp. Atlas; Data from Pan-Cancer Analysis of Whole Genomes Project
Glioblastoma multiforme	Human glioblastoma multiforme tumors - putamen	*Osgin2* upregulation	RNA-Seq	Exp. Atlas; Data from Pan-Cancer Analysis of Whole Genomes Project
Glioblastoma multiforme	Human glioblastoma multiforme tumors - substantia nigra	*Osgin1* downregulation	RNA-Seq	Exp. Atlas; Data from Pan-Cancer Analysis of Whole Genomes Project
Glioblastoma multiforme	Human glioblastoma multiforme tumors - substantia nigra	*Osgin2* upregulation	RNA-Seq	Exp. Atlas; Data from Pan-Cancer Analysis of Whole Genomes Project
Glioblastoma multiforme	Human glioblastoma multiforme tumors - Brodmann area 24	*Osgin1* upregulation	RNA-Seq	Exp. Atlas; Data from Pan-Cancer Analysis of Whole Genomes Project
Glioblastoma multiforme	Human glioblastoma	*Osgin2* upregulation	RNA-Seq	Exp. Atlas; Data from Pan-

	multiforme tumors - Brodmann area 24			Cancer Analysis of Whole Genomes Project
Glioblastoma multiforme	Human glioblastoma multiforme tumors - Brodmann area 9	*Osgin1* upregulation	RNA-Seq	Exp. Atlas; Data from Pan-Cancer Analysis of Whole Genomes Project
Glioblastoma multiforme	Human glioblastoma multiforme tumors - Brodmann area 9	*Osgin2* upregulation	RNA-Seq	Exp. Atlas; Data from Pan-Cancer Analysis of Whole Genomes Project
Glioblastoma multiforme	Human glioblastoma multiforme tumors - C1 segments of cervical spinal cord	*Osgin1* downregulation	RNA-Seq	Exp. Atlas; Data from Pan-Cancer Analysis of Whole Genomes Project
Glioblastoma multiforme	Human glioblastoma multiforme tumors - C1 segments of cervical spinal cord	*Osgin2* upregulation	RNA-Seq	Exp. Atlas; Data from Pan-Cancer Analysis of Whole Genomes Project
Glioblastoma multiforme	Human glioblastoma multiforme tumors - caudate nucleus	*Osgin1* downregulation	RNA-Seq	Exp. Atlas; Data from Pan-Cancer Analysis of Whole Genomes Project
Glioblastoma	Human brain tissue with glioblastoma tumors	*Osgin2* upregulation	Microarray	Exp. Atlas; Birks et al., 2013
Glioma	Adult human high grade glioma tumors	*Osgin1* downregulation	Microarray	Exp. Atlas; Data from Griesinger et al., 2015

Glioma	Human glioma tumors - amygdala	*Osgin1* upregulation	RNA-Seq	Exp. Atlas; Data from Pan-Cancer Analysis of Whole Genomes Project
Glioma	Human glioma tumors - amygdala	*Osgin2* upregulation	RNA-Seq	Exp. Atlas; Data from Pan-Cancer Analysis of Whole Genomes Project
Glioma	Human glioma tumors - Brodmann area 24	*Osgin1* upregulation	RNA-Seq	Exp. Atlas; Data from Pan-Cancer Analysis of Whole Genomes Project
Glioma	Human glioma tumors - Brodmann area 24	*Osgin2* upregulation	RNA-Seq	Exp. Atlas; Data from Pan-Cancer Analysis of Whole Genomes Project
Glioma	Human glioma tumors - Brodmann area 9	*Osgin1* upregulation	RNA-Seq	Exp. Atlas; Data from Pan-Cancer Analysis of Whole Genomes Project
Glioma	Human glioma tumors - Brodmann area 9	*Osgin2* upregulation	RNA-Seq	Exp. Atlas; Data from Pan-Cancer Analysis of Whole Genomes Project
Glioma	Human glioma tumors - C1 segments of cervical spinal cord	*Osgin1* upregulation	RNA-Seq	Exp. Atlas; Data from Pan-Cancer Analysis of Whole Genomes Project
Glioma	Human glioma tumors - C1 segments of cervical spinal cord	*Osgin2* upregulation	RNA-Seq	Exp. Atlas; Data from Pan-Cancer Analysis of Whole Genomes Project

Glioma	Human glioma tumors - caudate nucleus	*Osgin1* upregulation	RNA-Seq	Exp. Atlas; Data from Pan-Cancer Analysis of Whole Genomes Project
Glioma	Human glioma tumors - caudate nucleus	*Osgin2* upregulation	RNA-Seq	Exp. Atlas; Data from Pan-Cancer Analysis of Whole Genomes Project
Glioma	Human glioma tumors - cerebellar hemisphere	*Osgin1* upregulation	RNA-Seq	Exp. Atlas; Data from Pan-Cancer Analysis of Whole Genomes Project
Glioma	Human glioma tumors - cerebellar hemisphere	*Osgin2* upregulation	RNA-Seq	Exp. Atlas; Data from Pan-Cancer Analysis of Whole Genomes Project
Glioma	Human glioma tumors - cerebellum	*Osgin1* upregulation	RNA-Seq	Exp. Atlas; Data from Pan-Cancer Analysis of Whole Genomes Project
Glioma	Human glioma tumors - cerebellum	*Osgin2* upregulation	RNA-Seq	Exp. Atlas; Data from Pan-Cancer Analysis of Whole Genomes Project
Glioma	Human glioma tumors - cerebral cortex	*Osgin1* upregulation	RNA-Seq	Exp. Atlas; Data from Pan-Cancer Analysis of Whole Genomes Project
Glioma	Human glioma tumors - cerebral cortex	*Osgin2* upregulation	RNA-Seq	Exp. Atlas; Data from Pan-Cancer Analysis of Whole Genomes Project

Glioma	Human glioma tumors - hippocampus proper	*Osgin1* upregulation	RNA-Seq	Exp. Atlas; Data from Pan-Cancer Analysis of Whole Genomes Project
Glioma	Human glioma tumors - hippocampus proper	*Osgin2* upregulation	RNA-Seq	Exp. Atlas; Data from Pan-Cancer Analysis of Whole Genomes Project
Glioma	Human glioma tumors - hypothalamus	*Osgin1* upregulation	RNA-Seq	Exp. Atlas; Data from Pan-Cancer Analysis of Whole Genomes Project
Glioma	Human glioma tumors - hypothalamus	*Osgin2* upregulation	RNA-Seq	Exp. Atlas; Data from Pan-Cancer Analysis of Whole Genomes Project
Glioma	Human glioma tumors - nucleus accumbens	*Osgin1* upregulation	RNA-Seq	Exp. Atlas; Data from Pan-Cancer Analysis of Whole Genomes Project
Glioma	Human glioma tumors - nucleus accumbens	*Osgin2* upregulation	RNA-Seq	Exp. Atlas; Data from Pan-Cancer Analysis of Whole Genomes Project
Glioma	Human glioma tumors - putamen	*Osgin1* upregulation	RNA-Seq	Exp. Atlas; Data from Pan-Cancer Analysis of Whole Genomes Project
Glioma	Human glioma tumors - putamen	*Osgin2* upregulation	RNA-Seq	Exp. Atlas; Data from Pan-Cancer Analysis of Whole Genomes Project

Glioma	Human glioma tumors - substantia nigra	*Osgin1* upregulation	RNA-Seq	Exp. Atlas; Data from Pan-Cancer Analysis of Whole Genomes Project
Glioma	Human glioma tumors - substantia nigra	*Osgin2* upregulation	RNA-Seq	Exp. Atlas; Data from Pan-Cancer Analysis of Whole Genomes Project
Head and Neck Squamous Cell Carcinoma	Human mouth mucosa cells	*Osgin2* upregulation	RNA-Seq	Exp. Atlas; Data from Pan-Cancer Analysis of Whole Genomes Project
Hepatocellular carcinoma	Human liver cells	*Osgin1* upregulation	RNA-Seq	Exp. Atlas; Data from Pan-Cancer Analysis of Whole Genomes Project
Hepatocellular carcinoma	Human liver cells	*Osgin2* upregulation	RNA-Seq	Exp. Atlas; Data from Pan-Cancer Analysis of Whole Genomes Project
Invasive lobular carcinoma	Human breast tumor samples	*Osgin1* downregulation	RNA-Seq	Exp. Atlas; Data from Pan-Cancer Analysis of Whole Genomes Project
Invasive lobular carcinoma	Human breast tumor samples	*Osgin2* upregulation	RNA-Seq	Exp. Atlas; Data from Pan-Cancer Analysis of Whole Genomes Project
Lung Adenocarcinoma	Human lung cells	*Osgin1* downregulation	RNA-Seq	Exp. Atlas; Data from Pan-Cancer Analysis of Whole Genomes Project

Lung Adenocarcinoma	Human lung cells	*Osgin2* downregulation	RNA-Seq	Exp. Atlas; Data from Pan-Cancer Analysis of Whole Genomes Project
Lung Carcinoma	*Mus musculus* CD11b positive, Ly6G positive lung neutrophils	*Osgin1* downregulation	RNA-Seq	Exp. Atlas; Data from Choi et al., 2015
Lung Carcinoma	*Mus musculus* CD11b positive, Ly6G positive lung neutrophils	*Osgin2* downregulation	RNA-Seq	Exp. Atlas; Data from Choi et al., 2015
Lung Carcinoma	*Mus musculus* lung cells	*Osgin1* upregulation	RNA-Seq	Exp. Atlas; Data from Choi et al., 2015
Lymphoma	Human blood cells	*Osgin1* downregulation	RNA-Seq	Exp. Atlas; Data from Pan-Cancer Analysis of Whole Genomes Project
Lymphoma	Human blood cells	*Osgin2* upregulation	RNA-Seq	Exp. Atlas; Data from Pan-Cancer Analysis of Whole Genomes Project
Medulloblastom a	Human pediatric medulloblastoma tumors	*Osgin1* downregulation	Microarray	Exp. Atlas; Data from Birks et al., 2013
Medulloblastom a	Human group 4 medulloblastoma tumor samples	*Osgin1* downregulation	Microarray	Exp. Atlas; Data from Griesinger et al., 2015
Melanoma	Human skin tissue samples	*Osgin1* upregulation	RNA-Seq	Exp. Atlas; Data from Pan-Cancer Analysis of Whole Genomes Project

Melanoma	Human skin tissue samples	*Osgin2* upregulation	RNA-Seq	Exp. Atlas; Data from Pan-Cancer Analysis of Whole Genomes Project
Non-inflammatory Breast cancer	Human breast cancer tumors	*Osgin2* upregulation	Microarray	Exp. Atlas; Data from Woodward et al., 2013
Osteosarcoma	*Mus musculus* osteosarcoma samples	*Osgin1* downregulation	RNA-Seq	Exp. Atlas; Data from Straessler et al., 2013
Osteosarcoma	Human bone tissue samples with osteosarcoma tumors	*Osgin2* upregulation	Microarray	Exp. Atlas; Data from Jones et al., 2012
Ovarian Adenocarcinoma	Human ovarian tissue samples	*Osgin1* downregulation	RNA-Seq	Exp. Atlas; Data from Pan-Cancer Analysis of Whole Genomes Project
Ovarian Adenocarcinoma	Human ovarian tissue samples	*Osgin2* upregulation	RNA-Seq	Exp. Atlas; Data from Pan-Cancer Analysis of Whole Genomes Project
Pancreatic Adenocarcinoma	Human pancreatic cells	*Osgin1* upregulation	RNA-Seq	Exp. Atlas; Data from Pan-Cancer Analysis of Whole Genomes Project
Pancreatic Adenocarcinoma	Human pancreatic cells	*Osgin2* upregulation	RNA-Seq	Exp. Atlas; Data from Pan-Cancer Analysis of Whole Genomes Project

Pediatric High Grade Glioma	Human glioma tumors	*Osgin2* upregulation	Microarray	Exp. Atlas; Griesinger et al., 2015
Posterior Fossa Group A Ependymoma	Human brain/spinal cord tumors	*Osgin2* upregulation	Microarray	Exp. Atlas; Data from Griesinger et al., 2015
Posterior Fossa Group B Ependymoma	Human brain/spinal cord tumors	*Osgin2* upregulation	Microarray	Exp. Atlas; Data from Griesinger et al., 2015
Primitive Neuroectodermal Tumors	Human brain tissue samples	*Osgin1* downregulation	Microarray	Exp. Atlas; Data from Birks et al., 2013
Prostate Adenocarcinoma	Human prostate gland cells	*Osgin1* upregulation	RNA-Seq	Exp. Atlas; Data from Pan-Cancer Analysis of Whole Genomes Project
Prostate Adenocarcinoma	Human prostate gland cells	*Osgin2* upregulation	RNA-Seq	Exp. Atlas; Data from Pan-Cancer Analysis of Whole Genomes Project
Renal cell carcinoma	Human kidney cells	*Osgin1* upregulation	RNA-Seq	Exp. Atlas; Data from Pan-Cancer Analysis of Whole Genomes Project
Renal cell carcinoma	Human kidney cells	*Osgin2* upregulation	RNA-Seq	Exp. Atlas; Data from Pan-Cancer Analysis of Whole Genomes Project
Skeletal Muscle Sarcoma	Human skeletal muscle tissue samples	*Osgin1* downregulation	RNA-Seq	Exp. Atlas; Data from Pan-Cancer Analysis of Whole Genomes Project
Skeletal Muscle	Human skeletal	*Osgin2*	RNA-Seq	Exp. Atlas;

Sarcoma	muscle tissue samples	upregulation		Data from Pan-Cancer Analysis of Whole Genomes Project
Squamous Cell Carcinoma	Human squamous cell carcinoma skin samples	*Osgin1* upregulation	RNA-Seq	Exp. Atlas
Squamous Cell Carcinoma	*Rattus norvegicus* tongue epithelial and submucosal fibroblasts	*Osgin1* upregulation	Microarray	Exp. Atlas; Data from Wu et al., 2016
Squamous Cell Lung Carcinoma	Human lung cells	*Osgin1* upregulation	RNA-Seq	Exp. Atlas; Data from Pan-Cancer Analysis of Whole Genomes Project
Squamous Cell Lung Carcinoma	Human lung cells	*Osgin2* downregulation	RNA-Seq	Exp. Atlas; Data from Pan-Cancer Analysis of Whole Genomes Project
Squamous Cell Lung Carcinoma	*Rattus norvegicus* tongue epithelial cells	*Osgin2* downregulation	Microarray	Exp. Atlas; Data from Wu et al., 2016
Synovial Sarcoma	*Mus musculus* synovial sarcoma samples	*Osgin1* downregulation	RNA-Seq	Exp. Atlas; Data from Straessler et al., 2013
Tongue squamous cell carcinoma	Human tongue squamous cell carcinoma cells	*Osgin1* downregulation	RNA-Seq	Exp. Atlas; Data from Marcinkiewicz et al., 2014
Transgenic induced cancer	Cancerous Skeletal Muscle Tissue of *Sus scrofa*	*Osgin1* downregulation	RNA-Seq	Exp. Atlas

Transgenic induced cancer	Cancerous Skeletal Muscle Tissue of *Sus scrofa*	*Osgin2* downregulation	RNA-Seq	Exp. Atlas

Supplementary Table S10. Differential *Osgin* expression comparing pathological to nonpathological cells and tissues (unless otherwise specified) in diseases other than cancers. *Exp. Atlas* – Expression Atlas, [97,102-104,174-178,179-186]. The paper following "Data from" provided raw data from which the proceeding database generated expression profiles/datasets. The databases list these papers as being sources for original data, so we have included them for complete transparency.

**Pathology**	**Cell/Tissue Type**	***Osgin* Expression**	**Method**	**Source**
Actinic Keratosis	Human actinic keratosis skin sample	*Osgin1* upregulation	RNA-Seq	Exp. Atlas
Axial Spondyloarthropathy	Human blood cells	*Osgin2* upregulation	Microarray	Exp. Atlas; Sharma et al., 2009
Azoospermia	Human testis tissue samples	*Osgin2* downregulation	Microarray	Exp. Atlas; Spiess et al., 2007
Chronic Pancreatitis	*Mus musculus* pancreatic tissue samples	*Osgin2* upregulation	Microarray	Exp. Atlas; Data from Ulmasov et al., 2013
Colitis	*Mus musculus* colon tissue samples	*Osgin2* upregulation	RNA-Seq	Exp. Atlas
COVID-19	Human COVID-19 patient colon samples	*Osgin2* upregulation	RNA-Seq	Exp. Atlas; Wu et al., 2020
Crohn's Disease	Human ileal tissue samples	*Osgin1* downregulation	RNA-Seq	Exp. Atlas; Data from Haberman et al., 2014

Cystic Fibrosis	Human airway cell lines	*Osgin1* upregulation	Microarray	Exp. Atlas; Data from Voisin et al., 2014
Duchenne Muscular Dystrophy	Immortalized myoblasts of dystrophic *Mus musculus*	*Osgin1* downregulation	RNA-Seq	Exp. Atlas
Graft vs. Host Disease	*Mus musculus* lymph node fibroblastic reticular cells isolated from bone marrow transplant	*Osgin2* upregulation	RNA-Seq	Exp. Atlas
Graft vs. Host Disease	*Mus musculus* lymph node fibroblastic reticular cells isolated from bone marrow transplant	*Osgin1* downregulation	RNA-Seq	Exp. Atlas
Juvenile Dermatomyositis	Human muscle cells	*Osgin1* downregulation	Microarray	Exp. Atlas; Data from Chen et al., 2008
Kidney Transplant Rejection	Human kidney transplant samples	*Osgin1* downregulation	Microarray	Exp. Atlas; Data from Roedder et al., 2013
Klinefelter's Syndrome	Human induced pluripotent stem cells from fibroblasts of dermis of patient with Klinefelter's syndrome	*Osgin1* downregulation	RNA-Seq	Exp. Atlas
Nevus sebaceous of Jadassohn	Human nevus sebaceous of	*Osgin1* upregulation	RNA-Seq	Exp. Atlas

	Jadassohn skin sample			
Nevus sebaceous of Jadassohn	Human nevus sebaceous of Jadassohn skin sample	*Osgin2* downregulation	RNA-Seq	Exp. Atlas
NLRC4-Macrophage Activating Syndrome	Human blood cells	*Osgin1* upregulation	RNA-Seq	Exp. Atlas; Data from Canna et al., 2014
Non-alcoholic Steatohepatitis	Human liver tissue samples	*Osgin2* downregulation	Microarray	Exp. Atlas; Data from Frades et al., 2015
NSAID-exacerbated respiratory disease (N-ERD)	Human nasal poylp brushings	*Osgin1* downregulation	RNA-Seq	Exp. Atlas
Post-traumatic Osteoarthritis	*Sus scofa* synovial fluid samples of unilateral anterior cruciate ligament transections	*Osgin1* upregulation	RNA-Seq	Exp. Atlas
Progressive Supranuclear Palsy	Human medial temporal lobe tissue samples	*Osgin2* downregulation	Microarray	Exp. Atlas; Data from Bronner et al., 2009
Relapsing-Remitting Multiple Sclerosis	Human peripheral blood cells	*Osgin1* upregulation	RNA-Seq	Exp. Atlas; Data from Spurlock et al., 2015
Sepsis	Human whole blood cells	*Osgin1* upregulation	RNA-Seq	Exp. Atlas; Data from Linsley et al., 2014

Sepsis	Human whole blood cells	*Osgin2* downregulation	RNA-Seq	Exp. Atlas; Data from Linsley et al., 2014
Staphylococcus aureus cutaneous infection	*Mus musculus* skin cells	*Osgin2* downregulation	Microarray	Exp. Atlas; Data from Cho et al., 2012
Systemic-Onset Juvenile Idiopathic Arthritis	Human mononuclear blood cells at ages 3, 4, 5, 10, 15, and 16 years	*Osgin1* upregulation	RNA-Seq	Exp. Atlas
Teratozoospermi a	Human sperm cells	*Osgin2* downregulation	Microarray	Exp. Atlas; Data from Platts et al., 2007
Tetralogy of Fallot	Human heart right ventricle samples with Tetralogy of Fallot	*Osgin1* downregulation	RNA-Seq	Exp. Atlas; Data from Grunert et al., 2014
